# Disruption of estrogen receptor beta’s DNA binding domain impairs its tumor suppressive effects in triple negative breast cancer

**DOI:** 10.3389/fmed.2023.1047166

**Published:** 2023-02-28

**Authors:** Kirsten G. M. Aspros, Michael J. Emch, Xiyin Wang, Malayannan Subramaniam, Megan L. Hinkle, Esther P. B. Rodman, Matthew P. Goetz, John R. Hawse

**Affiliations:** ^1^Department of Biochemistry and Molecular Biology, Mayo Clinic, Rochester, MN, United States; ^2^Department of Oncology, Mayo Clinic, Rochester, MN, United States

**Keywords:** estrogen receptor beta, breast cancer, triple negative breast cancer, NFκB, EZH2, DNA binding domain mutation

## Abstract

Triple negative breast cancer (TNBC) is an aggressive sub-type of the disease which accounts for a disproportionately high percentage of breast cancer morbidities and mortalities. For these reasons, a better understanding of TNBC biology is required and the development of novel therapeutic approaches are critically needed. Estrogen receptor beta (ERβ) is a reported tumor suppressor that is expressed in approximately 20% of primary TNBC tumors, where it is associated with favorable prognostic features and patient outcomes. Previous studies have shown that ERβ mediates the assembly of co-repressor complexes on DNA to inhibit the expression of multiple growth promoting genes and to suppress the ability of oncogenic transcription factors to drive cancer progression. To further elucidate the molecular mechanisms by which ERβ elicits its anti-cancer effects, we developed MDA-MB-231 cells that inducibly express a mutant form of ERβ incapable of directly binding DNA. We demonstrate that disruption of ERβ’s direct interaction with DNA abolishes its ability to regulate the expression of well characterized immediate response genes and renders it unable to suppress TNBC cell proliferation. Loss of DNA binding also diminishes the ability of ERβ to suppress oncogenic NFκB signaling even though it still physically associates with NFκB and other critical co-factors. These findings enhance our understanding of how ERβ functions in this disease and provide a model system that can be utilized to further investigate the mechanistic processes by which ERβ elicits its anti-cancer effects.

## Introduction

Breast cancer is the most common malignancy and remains the second leading cause of cancer-related death among women worldwide (1) despite multiple advances in its clinical management over the past two decades (2). Breast cancer is a highly heterogeneous disease that is sub-typed based on molecular profiles which inform treatment plans. The primary subtypes of breast cancer are determined by protein expression levels of estrogen receptor alpha (ERα), progesterone receptor (PR), and human epidermal growth factor receptor 2 (HER2). Tumors lacking expression of these biomarkers are categorized together and referred to as basal-like or triple negative breast cancer (TNBC). TNBC accounts for 15–20% of all breast cancer cases but is responsible for a disproportionately higher number of breast cancer mortalities due to its aggressive nature, the lack of highly effective adjuvant therapies, and the high rates of resistance to standard-of-care chemotherapy regimens (3–5).

Numerous studies have reported the expression of a second form of the estrogen receptor, estrogen receptor beta (ERβ), in TNBC (6). Using a highly validated monoclonal antibody (7, 8), we and others have demonstrated that ERβ1, the full length form of this receptor, is expressed in approximately 20% of primary TN breast tumors (6, 8–10). Tumoral expression of ERβ is generally associated with better prognostic factors such as lymph node negativity, enhanced responses to therapy, and improved patient outcomes such as improved disease-free, metastasis-free, and overall survival (6, 10–13). Ligand-mediated activation of ERβ has been shown to decrease proliferation, invasion, and migration of TNBC cells *in vitro* as well as tumor formation, growth, and metastatic spread *in vivo* (10, 14–16). Given these anti-cancer properties of ERβ in TNBC, it is critical to better understand the mechanisms by which it elicits these effects and the clinical situations in which ERβ targeted therapies are most likely to be effective.

Estrogen receptors (ERs) utilize multiple mechanisms to modify target gene expression. Following binding by estradiol (E2), ERs dimerize, translocate to the nucleus, and canonically bind to estrogen response elements (EREs) through their zinc finger domain to regulate gene transcription (17, 18). However, ERs can also alter gene transcription through non-canonical mechanisms that do not require direct association with an ERE (17, 18). In these scenarios, ERs have been shown to associate with, or tether to, other transcription factors bound to their own DNA response elements through protein/protein interactions including nuclear factor kappa b (NFκB), signal transducer and activator of transcription (STATs), and activator protein-1 (AP1) factors among others (17–19). Through these types of mechanisms, ERs do not necessarily require direct DNA binding to affect target gene expression (17–19). Further, ERs can function outside of the nucleus to regulate the activity of phosphorylation-mediated signaling cascades that ultimately impact gene expression profiles (17–19).

We have shown that ERβ induces the formation of a co-repressor complex involving ERβ, the p65 component of NFκB, and enhancer of zeste homolog 2 (EZH2) to suppress oncogenic p65 signaling in TNBC (10). Mechanistically, we found that in response to E2, ERβ associated with chromatin near many well-known p65 target genes and demonstrated that ERβ binding to these loci resulted in decreased expression of these nearby genes (10). ChIP-seq and ChIP-PCR studies demonstrated that NFκB/p65 was also localized to many of these sites (10). Motif analysis of ERβ ChIP-seq data identified ERβ association with both EREs and NFκB/p65 response elements (NREs) at enhancer and promoter regions of NFκB/p65 target genes (10). Interestingly, multiple ERβ target genes did not contain a nearby ERE; rather they contained NREs, suggesting that ERβ was tethered to these sites through its interaction with p65. From a biological standpoint, we demonstrated that disruptions in the ability of ERβ to interact with p65 and suppress p65 target gene expression impaired its capacity to elicit anti-cancer effects (10).

Given the potential of ERβ as an important prognostic and predictive biomarker and therapeutic target in TNBC, we sought to further investigate its molecular mechanisms of action and biological functions in this form of the disease. More specifically, we aimed to determine the necessity of direct DNA binding by ERβ in suppressing NFκB transcriptional activity and eliciting anti-cancer effects. Towards this goal, we generated a TNBC cell line inducibly expressing a mutant form of ERβ in which two amino acids (E167A/G168A) critical for proper folding of one of ERβ’s zinc fingers were mutated generating a form of the receptor that is incapable of directly binding DNA (ERβ^DBD-Mut^) (20). Using this model, we demonstrated that this mutant form of ERβ was unable to perform known functions of wildtype ERβ (Erβ^WT^) in response to E2 treatment including the induction of cell cycle arrest and suppression of proliferation. RNA-seq studies revealed that estrogen treatment of ERβ^DBD-Mut^ expressing cells had very little impact on the regulation of gene expression and failed to suppress NFκB signaling. Interestingly, the inability of ERβ^DBD-Mut^ to suppress NFκB signaling occurred even though ERβ^DBD-Mut^ maintained its ability to interact with NFκB. Additionally, despite being able to interact with EZH2, ERβ^DBD-Mut^ no increases in deposition of the repressive histone 3 lysine 27 trimethylation mark (H3K27Me3) were observed. Taken together, we conclude that the anti-cancer effects of ERβ in TNBC are largely dependent on its ability to directly interact with DNA, and that failure to do so renders ERβ incapable of suppressing oncogenic NFκB signaling and functioning as a tumor suppressor.

## Methods

### Materials and reagents

Doxycycline (dox), 17β-estradiol (E2), and TNFα were purchased from Sigma-Aldrich (St. Louis, MO). The synthetic ERβ agonist LY500307 (LY) was provided by Eli Lilly & Co. (Indianapolis, IN). Fulvestrant (ICI182,780) was purchased from Tocris Bioscience (Bristol, United Kingdom).

### Cell culture

MDA-MB-231, Hs578T and MDA-MB-468 cells were obtained from ATCC and maintained in phenol red-free DMEM/F12 medium containing 10% fetal bovine serum (FBS) and 1% antibiotic-antimycotic (AA). Dox-inducible MDA-MB-231-ERβ^WT^ cells were developed as previously described (9). ERβ^DBD-Mut^ expressing cells were generated in an identical manner following QuikChange® PCR-mediated mutation of 2 critical amino acid residues in the first ERβ zinc finger (E167A/G168A). WT ERβ and ERβ^DBD-Mut^ cell lines were maintained in the same medium supplemented with 500 mg/L zeocin and 5 mg/L blasticidin S. ERβ^WT^ and ERβ^DBD-Mut^ expressing cells were also infected at an MOI of 5 with the IncuCyte® Cell Cycle Green/Red Lentivirus Reagent (#4779, Essen Bioscience Inc., Ann Arbor, MI), and pooled stably expressing cell lines were selected with and maintained in medium further supplemented with 500 μg/L puromycin. All experiments utilizing ERβ ligands were conducted in medium supplemented with 10% triple charcoal/dextran-stripped FBS (HyClone™, GE Healthcare Life Sciences, Pittsburg, PA).

### Real-time qPCR

Cells were plated in 12-well plates in dox-containing medium and treated with ethanol vehicle (veh) or 1 nM E2 for 5 days, with a media and treatment refreshment on day 3. If applicable, 20 ng/ml TNFα was added for 24 h after the initial 5 day ERβ ligand treatment. RNA was isolated using TRIzol™ Reagent. cDNA was generated from 1 μg of total RNA *via* an iScript™ cDNA Synthesis Kit (Bio Rad, Hercules, CA), and subsequently used for real-time quantitative PCR (RT-qPCR) using a PerfeCTa™ SYBR Green Fast Mix™ (Quanta Biosciences, Gaithersburg, MD) and a Bio-Rad CFX Real-Time PCR detection system. Expression of all genes was normalized to HPRT1 as a control. Primer sequences are listed in Supplementary Table 1.

### Western blotting

Cells were seeded in 6-well plates and treated for 24 h with or without dox. Cell lysates were harvested, and protein concentrations were determined using a Bradford assay. Equal amounts of protein were separated on 7.5% SDS polyacrylamide gels and transferred to PVDF membranes. Membranes were blocked for 1 h at room temperature using 5% milk in TBST and subjected to overnight incubation with primary antibody at 4°C. Following washing with 1X TBST, secondary antibody was added at room temperature for 1 h, followed by another set of washing. Blots were imaged on the Odessy Fc (LI-COR, Licoln, NE) system using the chemiluminescent (10 min) and 700 nm channels (30 s) for detection of protein and ladder, respectively. Antibody information can be found in Supplementary Table 2.

### Luciferase assays

Cells were seeded in 24-well plates in replicates of six. Twenty-four hours after seeding, cells were transfected with 100 ng/well of a pGL3 luciferase reporter construct containing either estrogen response elements (ERE) or NFκB response elements (NREs) using FuGENE 6 (Promega, Madison, WI). One day after transfection, cells were treated with ethanol vehicle, 1 nM E2, 20 ng/ml TNFα, or E2 + TNFα for 24 h. Cells were washed once with PBS and subsequently lysed using 1X Passive Lysis Buffer (Promega). Equal amounts of protein lysate were assayed for luciferase activity after addition of Luciferase Assay Reagent using a Glomax-Dual Luminometer (Promega).

### Chromatin immunoprecipitation followed by PCR

Cells were seeded in 10 cm dishes and treated for 5 days in triplicate with vehicle control or 1 nM E2, with one media change and treatment refresh on day 3. Cells were fixed for 10 min at room temperature with 1% paraformaldehyde, followed by quenching with 125 mM Glycine (Sigma) for 5 min. Nuclear extracts were isolated and immunoprecipitations were performed as previously described (21, 22). ChIP-PCR was performed in the same manner as RT-qPCR using ChIP-specific primers (Supplementary Table 3) and chromatin solution.

### Proliferation assays

MDA-MB-231- ERβ^WT^ and -ERβ^DBD-Mut^ cells were plated at a density of 1,000 cells per well in 96-well plates in replicates of 8 and treated with dox at the time of seeding. Following 24 h of attachment, cells were treated as indicated (ethanol vehicle, 1 nM E2, 1 μM ICI) and allowed to proliferate. After 7 days of treatment, cells were fixed with 25% (v/v) glutaraldehyde (Sigma) for 10 min, washed 5 times with water, stained with Crystal Violet (Sigma), and washed again. Crystal violet was solubilized using 100 μl of a 100 nM sodium citrate solution in 50% ethanol and quantified at 550 nm excitation with a plate reader.

### Proliferation assays following transient transfection

MDA-MB-231, Hs578T and MDA-MB-468 cells were plated at a density of 1,500 cells per well in 96-well plates in replicates of 8. At the time of plating, cells were transfected with 10 ng per well of both a YFP expression vector (pcDNA6.2 N-YFP-DEST) and either the ERβ^WT^ or ERβ^DBD-Mut^ expression vector (pcDNA4.0) using FuGENE 6 transfection reagent (Promega). Cells were allowed to adhere overnight and subsequently treated with vehicle control or 1 nM E2. Proliferation of YFP positive cells was monitored over a period of 24 h in an IncuCyte® S3 instrument (Essen Bioscience Inc.). Growth rates were determined by calculating the relative YFP area per confluence area following normalization to time zero (start of treatment).

### Cell cycle analysis

MDA-MB-231-ERβ^WT^ and -ERβ^DBD-Mut^ cells expressing the Cell Cycle Red/Green marker (described above) were seeded in 96-well plates and treated with dox 24 h prior to indicated treatments. Following addition of treatments, plates were placed in the IncuCyte® S3 instrument (Essen Bioscience Inc.) and imaged once every 2 h for 36 h on the phase, red fluorescent, and green fluorescent channels. The percent of cells in each phase were calculated according to the manufacturer’s recommendations.

### RNAseq

MDA-MB-231-ERβ^WT^ and -ERβ^DBD-Mut^ cells were plated in triplicate in 10 cm dishes and treated with dox plus ethanol vehicle or 1 nM E2 for a total of 5 days with a media change and treatment refresh on day 3. Total RNA was isolated using TRIzol™ Reagent (Thermo Fischer Scientific, Waltham, MA) and a miRNeasy Mini kit (Qiagen, Hilden, Germany) following the manufacturer’s instructions. Total RNA was submitted to the Mayo Clinic Genome Analysis Core (Rochester, MN) for sequencing and 200 ng was used for library preparation using the TruSeq Stranded mRNA Sample Prep Kit (Illumina, San Diego, CA) according to the manufacturer’s protocol. The Illumina HiSeq 4,000 sequencer and software (HD 3.4.0.38) was used to prepare 50 base-pair paired-end reads with approximately 50 million fragment reads per sample. Base-calling was conducted using RTA version 2.7.7 from Illumina. The Mayo Clinic Medical Genome Facility Genome Analysis Core performed library preparation and primary analysis. Mapped reads were assigned using featureCounts and an RPKM<1.0 cutoff was applied to remove lowly expressed genes. Differential analysis was completed using edgeR and significance was measured using |log_2_(fold change)| ≥1.0 and FDR ≤0.1.

### Cell fractionation and co-immunoprecipitation

HEK293T cells were plated in replicates of 3 in 10 cm dishes and allowed to adhere for 24 h, at which time they were transfected with 5 μg empty vector (pcDNA4), ERβ^WT^, or ERβ^DBD-Mut^ plasmids and 15 μg of a myc-tagged EZH2 expression vector. After 24 h, medium was removed, cells were washed with cold PBS, and pellets were collected in 1X PBS for nuclear, cytoplasmic, or whole cell lysate preparation as previously described. After protein concentrations were determined, 40 μg of protein were used as inputs for western blotting, and 500 μg were used for overnight immunoprecipitation at 4°C with rotation. Following immunoprecipitation, protein complexes were captured using 40 μl Protein G Dynabeads™ (Thermo Fisher) for 2 h at 4°C with rotation. Beads were washed 3 times and protein was eluted from beads by boiling with 2X Laemmli Sample Buffer (Bio-Rad) containing β-mercaptoethanol for 5 min. All immunoprecipitated samples were subjected to western blotting. Antibody information can be found in Supplementary Table 2.

### Statistical analyses

All experiments were conducted in biological replicates of at least 3 and with 3–8 technical replicates per assay. Representative data sets have been selected for presentation in the figures. Student’s *t*-test and one-way ANOVAs were used to determine significant differences between treatments and *p*-values<0.05 were considered statistically significant. Graphs and analyses were conducted using GraphPad Prism 8 (GraphPad Software, San Diego, CA).

## Results

### Development and characterization of ERβ^DBD^ expressing MDA-MB-231 cell lines

**Figure 1:**
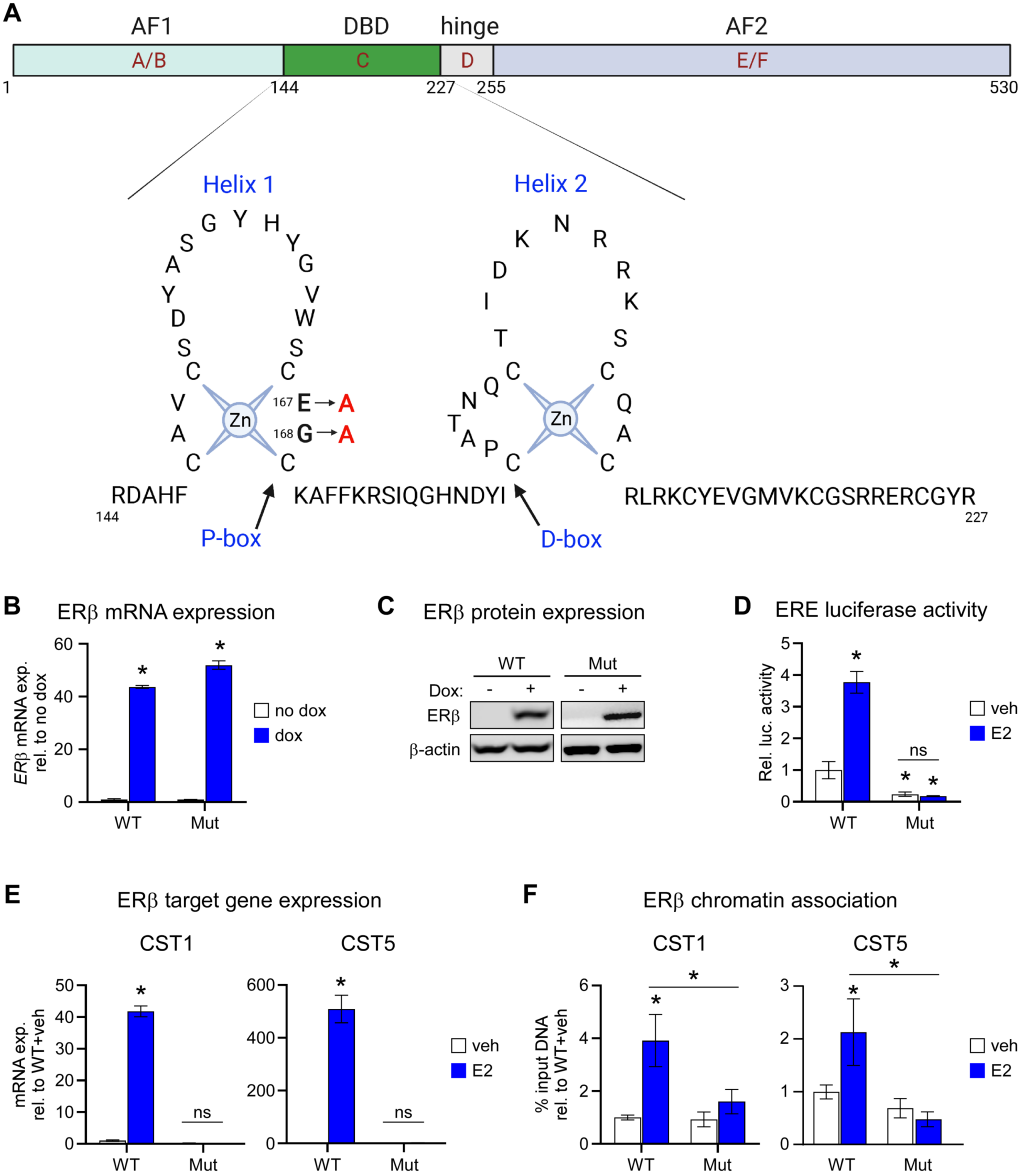
Characterization of the ERβ^DBD-Mut^ cell line. **(A)** Schematic depicting two dimensional domain structure of the ERβ protein with detailed resolution of amino acids 144–227 consisting of the helix 1 and helix 2 zinc fingers. The two amino acid mutations (amino acids 167 and 168) used to generate the ERβ^DBD-Mut^ utilized throughout this manuscript are highlighted. **(B)** mRNA and **(C)** protein expression of wild type ERβ (WT) and ERβ^DBD-Mut^ (Mut) in MDA-MB-231 cell lines following treatment with dox. β-actin shown as protein loading control. **(D)** Activity of an estrogen response element (ERE) luciferase reporter construct in WT and Mut MDA-MB-231 cells following veh or E2 treatment. **(E)** mRNA expression levels of CST1 and CST (known ERβ target genes) in WT and Mut MDA-MB-231 cells following veh or E2 treatment. **(F)** ChIP-PCR for ERβ at known ERβ binding sites located in the promoter of *CST1* and *CST5* in WT and Mut MDA-MB-231 cells following veh or E2 treatment. Graphs depict % input DNA relative to WT + veh. For all analyses, data is normalized to WT + veh levels and * indicates *p* < 0.05 relative to either no dox/veh treatment or between specific treatment conditions as indicated by horizontal lines following assessment by ANOVA.

We developed MDA-MB-231 cells with dox-inducible expression of a mutant form of flag-tagged ERβ that disrupts DNA binding (ERβ^DBD-Mut^; Figure 1A). MDA-MB-231- ERβ^DBD-Mut^ cells were shown to express similar levels of both ERβ mRNA and protein relative to the WT cell line (Figures 1B,C). As expected, ERβ^DBD-Mut^ did not induce the activity of an ERE luciferase reporter construct (Figure 1D) nor did it induce the expression of CST1 or CST5 which are known to be robustly induced by WT ERβ in response to E2 (15) (Figure 1E). Chromatin immunoprecipitation followed by polymerase chain reaction (ChIP-PCR) studies indicated that ERβ^DBD-Mut^ was not recruited to known ERβ binding sites (EREs) (15) nearby the CST1 and CST5 genes as was the case with WT ERβ (15) (Figure 1F).

### ERβ^DBD-Mut^ is incapable of suppressing proliferation and inducing cell cycle arrest of TNBC cells

**Figure 2:**
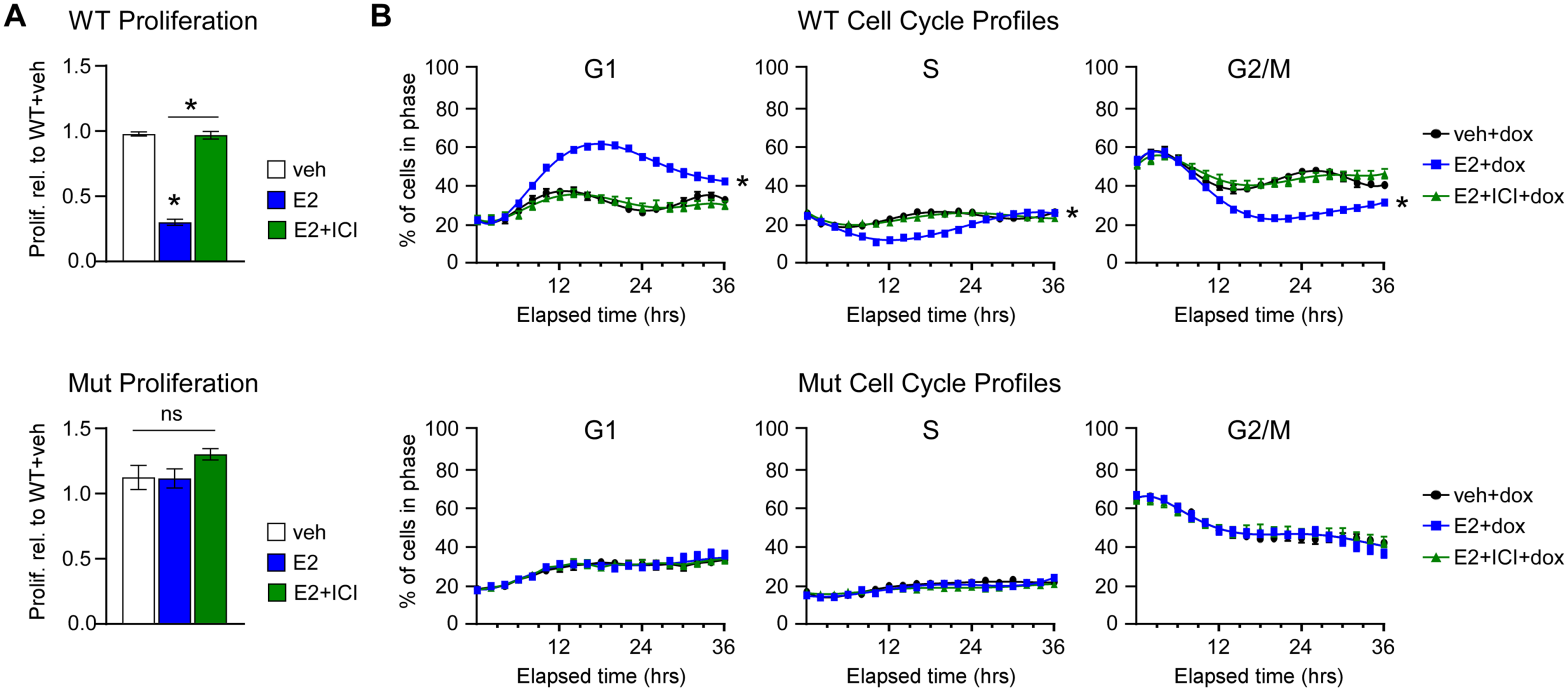
Effects of WT and ERβ^DBD-Mut^ on proliferation and cell cycle progression of MDA-MB-231 cells. **(A)** Proliferation of WT ERβ and ERβ^DBD-Mut^ (Mut) MDA-MB-231 cells in the presence of dox and veh, E2, or E2 + ICI treatment. * Indicates *p* < 0.05 relative to WT + veh or between indicated treatments (Student’s *t*-test). **(B)** Real-time assessment of ERβ WT and Mut MDA-MB-231 cell cycle progression in the presence of dox and veh, E2, or E2 + ICI. * Indicates *p* < 0.05 relative to veh treat cells (ANOVA).

Next, we assessed the effects of E2 on the proliferation rates and cell cycle profiles of MDA-MB-231-ERβ^DBD-Mut^ cells. Following E2 treatment, cells expressing WT ERβ exhibited significant decreases in cell proliferation at 7 days, effects that were blocked by the selective estrogen receptor degrader, ICI (Figure 2A). However, ERβ^DBD-Mut^ expressing cells were completely insensitive to E2 and ICI (Figure 2A). The effects of ERβ^DBD-Mut^ on cel proliferation were validated in MDA-BM-231 cells and additional TNBC cell lines (Hs578T and MDA-MB-468) following transient transfection of a YFP expression vector and either a ERβ^WT^ or ERβ^DBD-Mut^ expression vector. As with the dox-inducible MDA-MB-231 models, E2 treatment significantly inhibited the growth of ERβ^WT^ expressing cells, but no ERβ^DBD-Mut^ expressing cells following transfection (Supplementary Figure 1). Identical effects were also observed in Hs578T and MDA-MB-468 cell line models with the magnitude of the E2 effect being even stronger (Supplementary Figure 1).

We have previously shown that ERβ-mediated suppression of TNBC cell proliferation is due to induction of a G1 cell cycle arrest (14). We recapitulated these findings using the Cell Cycle Red/Green reporter in a real-time IncuCyte® assay which revealed accumulation WT ERβ cells in G1 following 8–12 h of E2 treatment with a concomitant decrease in the number of cells in S and G2/M phases of the cell cycle (Figure 2B). The effects of E2 on G1 cell cycle arrest in WT ERβ expressing cells were completely blocked by the addition of ICI (Figure 2B). As with the proliferation assays, E2 and ICI had no impact on cell cycle progression of MDA-MB-231-ERβ^DBD-Mut^ cells (Figure 2B). In the absence of ERβ expression, both cell lines progressed through the cell cycle in a similar manner regardless of treatment (Supplementary Figure 2).

### Mutation of ERβ’s DNA binding domain nearly abolishes its E2-mediated transcriptional activity

**Figure 3:**
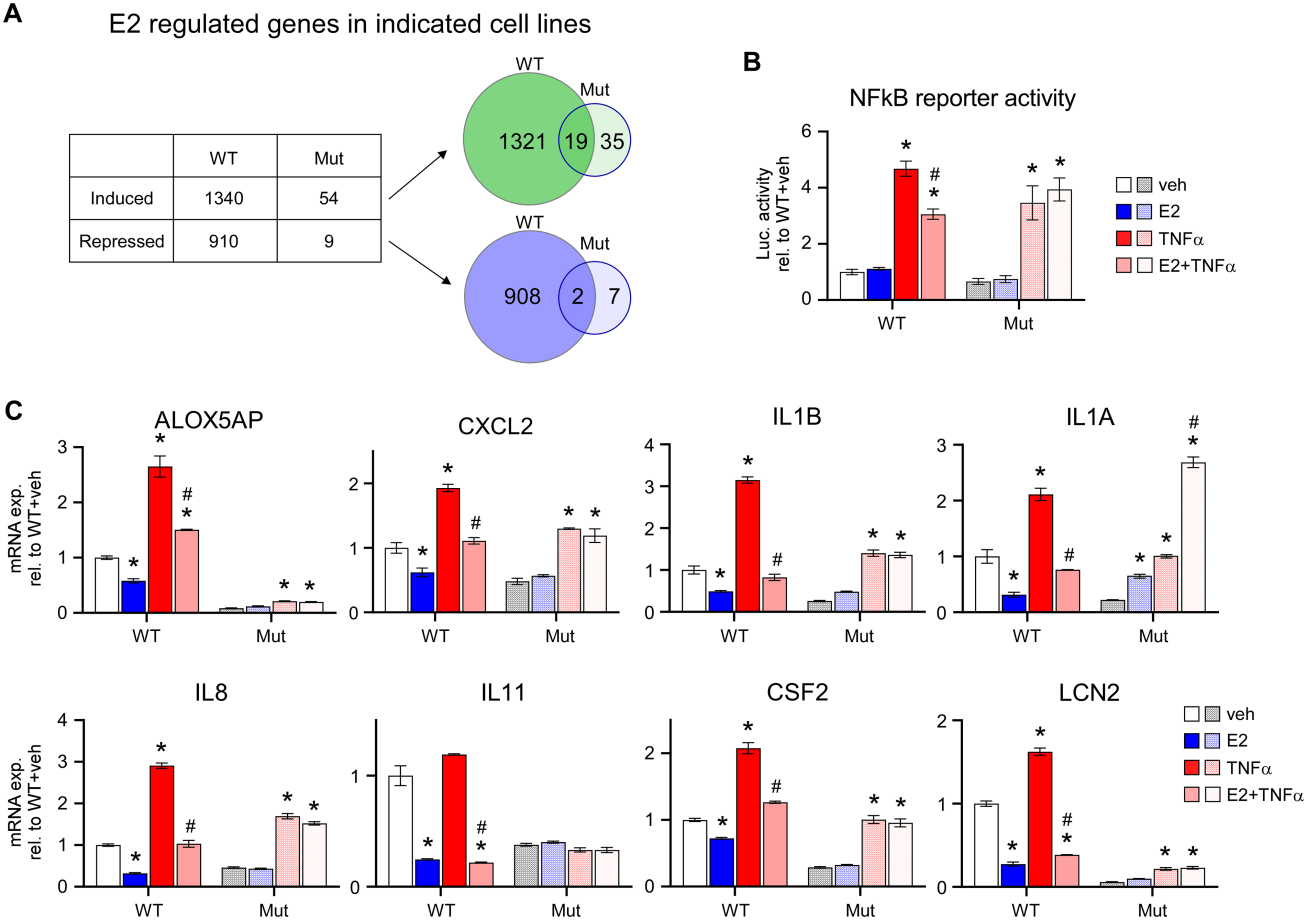
E2-mediated transcriptional activity of ERβ^DBD-Mut^. **(A)** Number of genes induced or repressed by WT ERβ or ERβ^DBD-Mut^ (Mut) following 5 days of E2 treatment as identified *via* RNAseq. Venn diagrams show overlap of genes induced (upper) and repressed (lower) by WT and Mut ERβ. **(B)** Activity of a NFκB luciferase reporter construct in WT and Mut ERβ expressing MDA-MB-231 cells following dox exposure and indicated treatments. **(C)** Expression of NFκB target genes in dox-induced WT and Mut MDA-MB-231-ERβ cells following indicated treatments. All data is normalized to WT ERβ + veh treated cells. * Indicates *p* < 0.05 relative to veh treatment and ^#^ indicates *p* < 0.05 relative to TNFα treatment for each cell line (ANOVA).

Given the inability of ERβ^DBD-Mut^ to induce well-known ERβ target genes, suppress proliferation and elicit cell cycle arrest in TNBC cells, we performed an unbiased assessment of its transcriptional activity using RNA-seq. These studies revealed that WT ERβ significantly regulated the expression of 2,250 genes, 1,340 of which were induced and 910 which were repressed, following 5 days of E2 treatment (Figure 3A; Supplementary Table 4). Somewhat surprisingly, only 63 genes were significantly regulated in ERβ^DBD-Mut^ cells (54 induced and 9 repressed; Figure 3A; Supplementary Table 5). Of these, 19 and 2 of the induced and repressed genes were similarly regulated by WT ERβ, respectively (Figure 3A; Supplementary Table 6). One of the mechanisms by which ERβ elicits its anti-cancer effects in TNBC cells is through suppression of oncogenic NFκB/p65 signaling (10). WT ERβ has been shown to inhibit NFκB target gene expression following E2 treatment in TNBC cells and to suppress TNFα-mediated activation of these same genes (10) (Figures 3B,C). Unlike WT ERβ, ERβ^DBD-Mut^ did not alter TNFα-induced activity of an NFκB luciferase reporter or expression of genes known to be regulated by NFκB following estrogen treatment (Figures 3B,C).

### ERβ^DBD-Mut^ interacts with NFκB and EZH2 but fails to enhance their recruit to chromatin in response to E2

**Figure 4:**
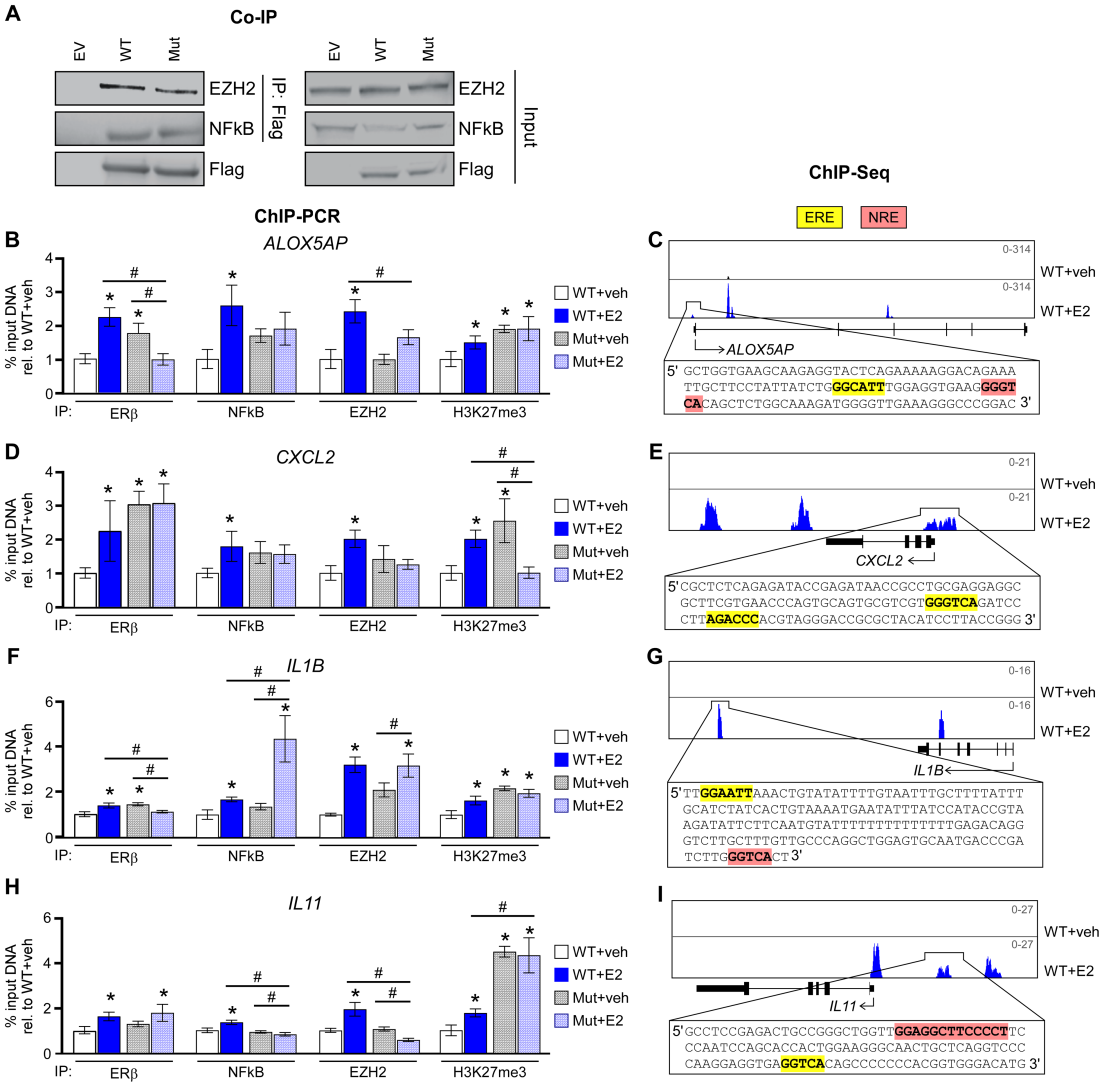
ERβ^DBD-Mut^ interacts with NFκB and EZH2 but fails to repress NFκB target gene expression. **(A)** Co-immunoprecipitation for WT ERβ and Mut ERβ followed by western blotting for EZH2 and NFκB in HEK293T transfected cells. Inputs, and transfections with empty expression vector, are shown as controls. **(B,D,F,H)** ChIP-PCR for ERβ, NFκB, EZH2, and H3K27me3 enrichment at known ERβ/NFκB binding sites in close proximity to NFκB target genes following dox exposure and indicated treatments of WT ERβ and ERβ^DBD-Mut^ (Mut) expressing MDA-MB-231 cells. Scale shown as % input DNA relative to WT + veh. * Indicates *p* < 0.05 relative to WT + veh cells and ^#^ represents *p* < 0.05 between indicated cell lines/treatments (ANOVA). **(C,E,G,I)** Genome browser tracks from ERβ ChIPseq experiments in WT ERβ expressing MDA-MB-231 cells treated with veh or E2. Insets display genomic sequences of depicted ERβ binding sites with estrogen response elements (EREs) and NFκB response elements (NREs) highlighted in yellow and pink, respectively.

We have previously reported the formation of a novel co-repressor complex driven by ERβ that involves NFκB and EZH2 in TNBC cells and have provided evidence that this complex plays an important role in mediating the anti-cancer effects of ERβ in TNBC (10). We thus next determined if the ERβ^DBD-Mut^ was capable of interacting with NFκB and EZH2. Indeed, co-immunoprecipitation (co-IP) assays confirmed an interaction between ERβ^DBD-Mut^, NFκB, and EZH2 that was very similar to that of WT ERβ (Figure 4A). Interestingly, the levels of ERβ^DBD-Mut^ found to be associated with chromatin were greater than that of ERβ^WT^ at four different loci of chromatin located in the proximity of known NFκB target genes (Figures 4B,D,F,H). With the exception of one of these loci, both consensus estrogen response elements (EREs) and NFκB response elements (NREs) are encoded within the DNA sequence (Figures 4C,E,G,I). Following E2 treatment, the levels of ERβ, NFκB, EZH2 and the transcriptionally repressive mark H3K27me3 were universally increased at these loci in cells expressing ERβ^WT^ (Figures 4B–I). With the exception of NFκB and EZH2 at the IL1B loci, E2 treatment did not enhance the levels of NFκB, EZH2 or H3K27me3 in ERβ^DBD-Mut^ expressing cells (Figures 4B–I).

### Clinically relevant mutations in the *ESR2* gene

**Figure 5:**
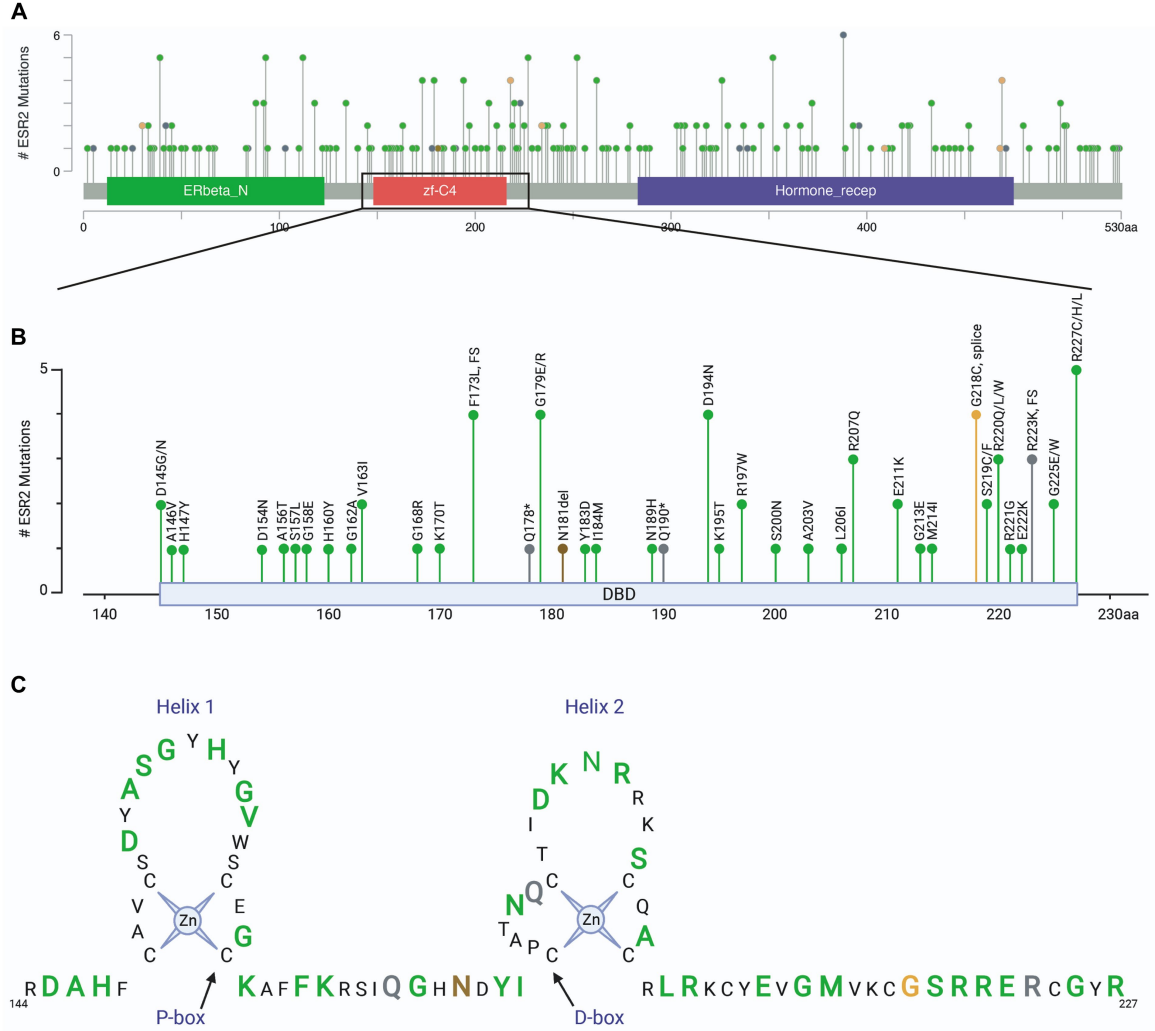
ESR2 mutations identified in clinical specimens. **(A)** Screen shot of all ESR2 mutations identified in 342 cBioPortal.org studies representing 165,871 patients. A total of 417 mutations were identified across 254 independent patient samples. **(B)** Inset highlighting 110 mutations identified specifically in the DNA binding domain. **(C)** Schematic highlighting DNA binding domain mutations where green represents missense mutations, grey represents truncations, brown represents inframe deletions/insertions and orange represents splice mutations. Amino acids depicted by smaller black text were not mutated in any of the samples represented in the 165,871 patients included in this cohort.

To relate these findings to clinically relevant situations, we assessed mutations in the *ESR2* gene using patient samples found in cBioPortal for Cancer Genomics (23, 24). Analysis included a total of 342 cBioPortal.org studies representing 165,871 patients. A total of 417 mutations were identified in *ESR2* across 254 independent patient samples (Figure 5A). Of these mutation calls, 110 were located in the DNA binding domain (Figure 5B). A schematic highlighting these DNA binding domain mutations is shown in Figure 5C by colored amino acids. These findings highlight the diversity of variants of uncertain significance that have thus far been identified in humans.

## Discussion

In this study, we aimed to further understand the importance of direct DNA binding by ERβ with regard to its anti-cancer effects in TNBC. We report the development of a novel TNBC cell line expressing a mutant form of ERβ in which one of its zinc fingers has been mutated to prevent direct binding to DNA. Although these cells express robust levels of the mutant ERβ mRNA and protein, this receptor was shown to be essentially transcriptionally dead, at least with regard to transcriptomic analyses following 5 days of E2 treatment. This mutant form of ERβ was also unable to induce cell cycle arrest and did not inhibit TNBC cell proliferation as was the case for WT ERβ. Mechanistically, ERβ^DBD-Mut^ did not exhibit increased association with chromatin in response to E2 at known sites containing an ERE. However, in some cases, the basal and/or E2 induced abundance of ERβ at chromatin sites encoding other transcription factor response elements, such as NREs, was observed. These associations likely occurred through tethering of ERβ to NFκB given the known protein interactions that occur between these two transcription factors in these cells (10). Indeed, ERβ^DBD-Mut^ remained capable of interacting with both NFκB and EZH2, members of a previously reported co-repressor complex involved in suppressing NFκB signaling in TNBC (10). However, the ERβ^DBD-Mut^-containing complex failed to result in H3K27me3 and subsequent chromatin compaction near NFκB target genes, ultimately rendering it incapable of suppressing NFκB target gene expression.

In humans, multiple ESR2 SNPs and polymorphisms have been reported (Figure 5), many of which are considered to be non-pathogenic or variants of uncertain significance. These VUS will need to be systematically analyzed in order to identify the precise effects on the function of this receptor. However, there are examples of a few of these alterations resulting in clinically relevant manifestations. These include lack of puberty and complete ovarian failure in a woman with a lysine to arginine mutation in residue 314 (25), primary amenorrhea in an individual with an alanine to asparagine change at amino acid 432 (26), contributions to increased risk of osteoporosis in women with alterations in amino acid 39 (27), and disorders of sex development in individuals with deletions of asparagine 181 or missense mutations in glycine 84 and leucine 426 (28). Given that these protein coding alterations are found throughout the ERβ protein sequence, and are not clustered specifically within the DNA binding domain, highlight the diverse functions of this receptor and suggest that disruption of DNA binding alone will not recapitulate all disorders associated with ERβ functions.

However, to get to the core functions of estrogen receptors as a transcription factors, multiple approaches have been employed to dissect the consequences of preventing direct DNA binding (19, 20, 29). Mutation of the equivalent amino acid residues of ERα (E207A/G208A), as were assessed for ERβ in the present report, resulted in infertile females with hyperplastic uteri in murine models (19). Interestingly, female mice completely lacking ERα instead developed hypoplastic uteri and incomplete mammary gland development (29). These discoveries revealed gain-of-function activities for ERα resulting from DNA binding domain mutations (ERα^DBD-Mut^) that are opposite of phenotypes reported in complete ERα knockout models. These contrasting findings are likely explained by the maintenance or development of novel transcriptional complexes orchestrated by mutant forms of ERα that cannot occur in the setting of complete ablation of ERα expression (19, 29). In these studies, ERα^DBD-Mut^ mice were more responsive to E2 with regard to ERα’s ability to induce expression of target genes including IGF-1 (19). Other studies examining the effects of disrupting ERβ association with chromatin have found that ERβ requires DNA binding to facilitate cross-talk with STAT5b and AP1 in cell line models (20). To date, no mouse models have been developed to explore the consequences of preventing DNA binding by ERβ, and therefore phenotypes resulting from such mutant forms of ERβ remain a mystery.

Here, we demonstrate that ERβ requires an intact DNA binding domain to suppress oncogenic NFκB signaling in TNBC. Our previous studies have indicated that suppression of oncogenic NFκB signaling in TNBC is an essential component of ERβ’s tumor suppressive properties in this disease context (10). We demonstrated that suppression of NFκB signaling occurs through formation of a novel co-repressor complex involving NFκB, EZH2, and other members of the PRC2 complex (10). Our findings reported here demonstrate that ERβ^DBD-Mut^ is still capable of interacting with NFκB and EZH2, and that basal levels of ERβ^DBD-Mut^ localized to chromatin nearby NFκB target genes is equivalent to or greater than that of WT ERβ. However, with the exception of NFκB and EZH2 at the IL1B locus, E2 treatment failed to enhance the levels of NFκB and EZH2 in ERβ^DBD-Mut^ expressing cells. Consequently, enhanced H3K27me3 was also not observed following E2 exposure, likely explaining the inability of ERβ^DBD-Mut^ to suppress NFκB target gene expression. While the basis for these observations is not completely clear, these results suggest that other critical co-factors are necessary for proper assembly of this co-repressor complex and that association/recruitment of these co-factors with ERβ is disrupted by mutations in its DNA binding domain. Further, it is clear that other transcriptional complexes that do not involve ERβ are at play in regulating the expression of NFκB target genes in TNBC cells. Identification of such transcription factor and co-regulators, as well as clarification of the mechanistic basis for these observations, require further study.

More broadly, our results indicate that ERβ^DBD-Mut^ is essentially transcriptionally dead relative to WT ERβ in response to E2 treatment. This was surprising given our hypothesis that ERβ^DBD-Mut^ would remain tethered to DNA through interactions with other transcription factors and co-regulators, and/or to alter gene expression through other non-classical mechanisms. It is important to acknowledge that these findings may not be generalizable to other cell lines or *in vivo* studies. Given that the ERβ^DBD-Mut^ utilized in the present report remains capable of interacting with NFκB and EZH2, and potentially many other co-factors, it is quite possible that substantial differences in transcriptional outputs would be observed in response to activation of other signaling pathways.

These findings are of importance to the field of breast cancer for several reasons. First, they further highlight the need to better understand ERβ biology in the context of TNBC from a personalized medicine standpoint in order to precisely identify patients who are most likely to benefit from ERβ-targeted therapies. Multiple SNPs in and around the ERβ gene, and specific point mutations within the coding sequence of ERβ have been identified in the general population (30–36) and in patient tumors as outlined here. However, their relative abundance in TN breast tumors has not been specifically studied. Based on the data presented here, germline or somatic mutations in the DNA binding domain of ERβ would likely abolish its tumor suppressive properties in TNBC patients. Further, assessment of somatic *ESR2* mutations that emerge in response to cancer therapies have not been explored. Indeed, hotspot mutations in the *ESR1* gene (ERα) have been shown to emerge following the development of endocrine therapy resistance in a large fraction of breast cancer patients (37–40). These mutations confer estrogen independent activity for ERα and result in a growth advantage for tumor cells in the setting of drugs designed to suppress estrogen signaling (37, 41, 42). Given the importance of ERβ’s DBD reported here, it is possible that loss-of-function mutations will be found in recurrent and highly aggressive forms of cancer, and/or in patients progressing on ERβ-targeted therapies.

Finally, these findings emphasize the importance of ERβ’s ability to repurpose EZH2 for tumor suppressive activities. Previous studies have demonstrated that in the absence of ERβ expression, EZH2 functions as an oncogene, in part through co-activation of NFκB to drive tumor progression (43). We have previously shown that when ERβ is expressed, it is able to switch these oncogenic properties of EZH2 for tumor suppressive purposes (10). Importantly, our findings suggest that the use of EZH2 inhibitors, which continue to undergo clinical development (44–46), may be contraindicated in tumors expressing WT ERβ. These possibilities require further study and our findings reporter here highlight the multitude of loss- and gain-of-function activities that may exist for ERβ depending on a given mutation and the disease context. Although there remains much to explore, findings from these studies have provided additional clarity regarding the molecular mechanisms by which ERβ elicits tumor suppressive effects in TNBC.

## Data availability statement

The original contributions presented in the study are included in the article/supplementary material. All genes determined to be significantly regulated in the reported cell lines are provided in the supplementary tables. Further inquiries can be directed to the corresponding author.

## Author contributions

KA, MS, and JH: concept and design. KA, ME, XW, MH, ER, and JH: collection and assembly of data. KA, ME, XW, MH, MG, and JH: data analysis and interpretation. KA and JH: manuscript writing. All authors contributed to the article and approved the submitted version.

## Funding

Funding of this research was provided by the National Cancer Institute of the National Institutes of Health under award numbers R01CA249116 (JH) and F31CA228193 (KA), the Mayo Clinic Breast Cancer SPORE (PC50CA116201; JH and MG), the Eisenberg Foundation (JH), the Mayo Foundation (JH), and the Mayo Clinic Graduate School of Biomedical Sciences (KA).

## Conflict of interest

The authors declare that the research was conducted in the absence of any commercial or financial relationships that could be construed as a potential conflict of interest.

## Publisher’s note

All claims expressed in this article are solely those of the authors and do not necessarily represent those of their affiliated organizations, or those of the publisher, the editors and the reviewers. Any product that may be evaluated in this article, or claim that may be made by its manufacturer, is not guaranteed or endorsed by the publisher.

## Supplementary material

SUPPLEMENTARY FIGURE 1 Effects of WT and ERβDBD-Mut on proliferation of MDA-MB-231, Hs578T and MDA-MB-468 cells. Proliferation of MDA-MB-231, Hs578T and, MDA-MB-468 cells in response to vehicle (Veh) or 1nM estradiol (E2) treatment following transient transfection of WT ERβ or ERβDBD-Mut with a YFP expression vector. * Indicates *p* < 0.05 relative between indicated treatments (Student’s *t*-test).

SUPPLEMENTARY FIGURE 2 Cell cycle analysis of WT and ERβDBD-Mut MDA-MB-231 cells in the absence of dox exposure. Real-time assessment of ERβ WT and Mut MDA-MB-231 cell cycle progression in the absence of dox exposure (i.e., absence of ERβ expression) following veh, E2, or E2+ICI treatment. No significant differences were detected (ANOVA).

SUPPLEMENTARY TABLE 1 Sequences of primers used for RT-qPCR.

SUPPLEMENTARY TABLE 2 Antibodies used for western blotting (WB) and immunoprecipitation (IP) including the company of purchase, catalog number, and dilution of primary antibody used in indicated assay.

SUPPLEMENTARY TABLE 3 Sequences of primers used for ChIP-PCR.

SUPPLEMENTARY TABLE 4 Genes significantly regulated following 5 days of 1nM E2 treatment of WT ERβ expressing MDA-MB-231 cells.

SUPPLEMENTARY TABLE 5 Genes significantly regulated following 5 days of 1nM E2 treatment of ERβDBD-Mut expressing MDA-MB-231 cells.

SUPPLEMENTARY TABLE 6 Genes commonly regulated following 5 days of 1nM E2 treatment of both WT ERβ and ERβDBD-Mut expressing MDA-MB-231 cells.
