# Supplementary figures and images for: Disruption of estrogen receptor beta’s DNA binding domain impairs its tumor suppressive effects in triple negative breast cancer
Source: Front Med (Lausanne). 2023 Feb 28;10:1047166. doi: 10.3389/fmed.2023.1047166 (PMC10011152; doi:10.3389/fmed.2023.1047166)

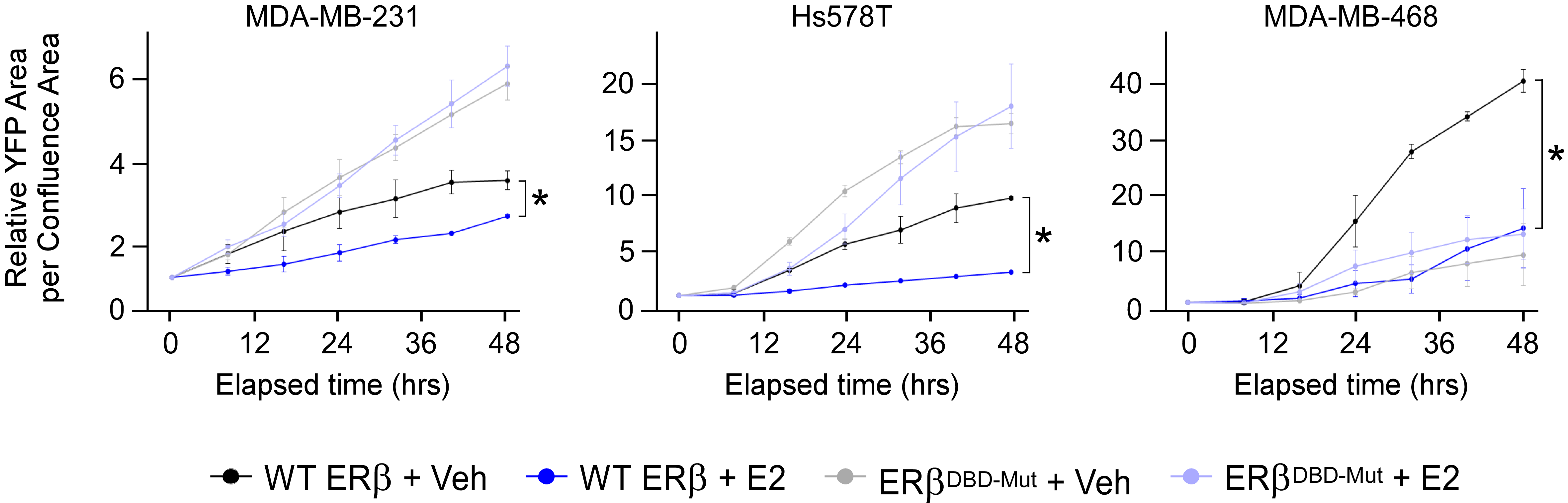

Supplement: SUPPLEMENTARY FIGURE 1 — Effects of WT and ERβDBD-Mut on proliferation of MDA-MB-231, Hs578T and MDA-MB-468 cells. Proliferation of MDA-MB-231, Hs578T and, MDA-MB-468 cells in response to vehicle (Veh) or 1nM estradiol (E2) treatment following transient transfection of WT ERβ or ERβDBD-Mut with a YFP expression vector. * Indicates p < 0.05 relative between indicated treatments (Student’s t-test). [file Image_1.TIF]

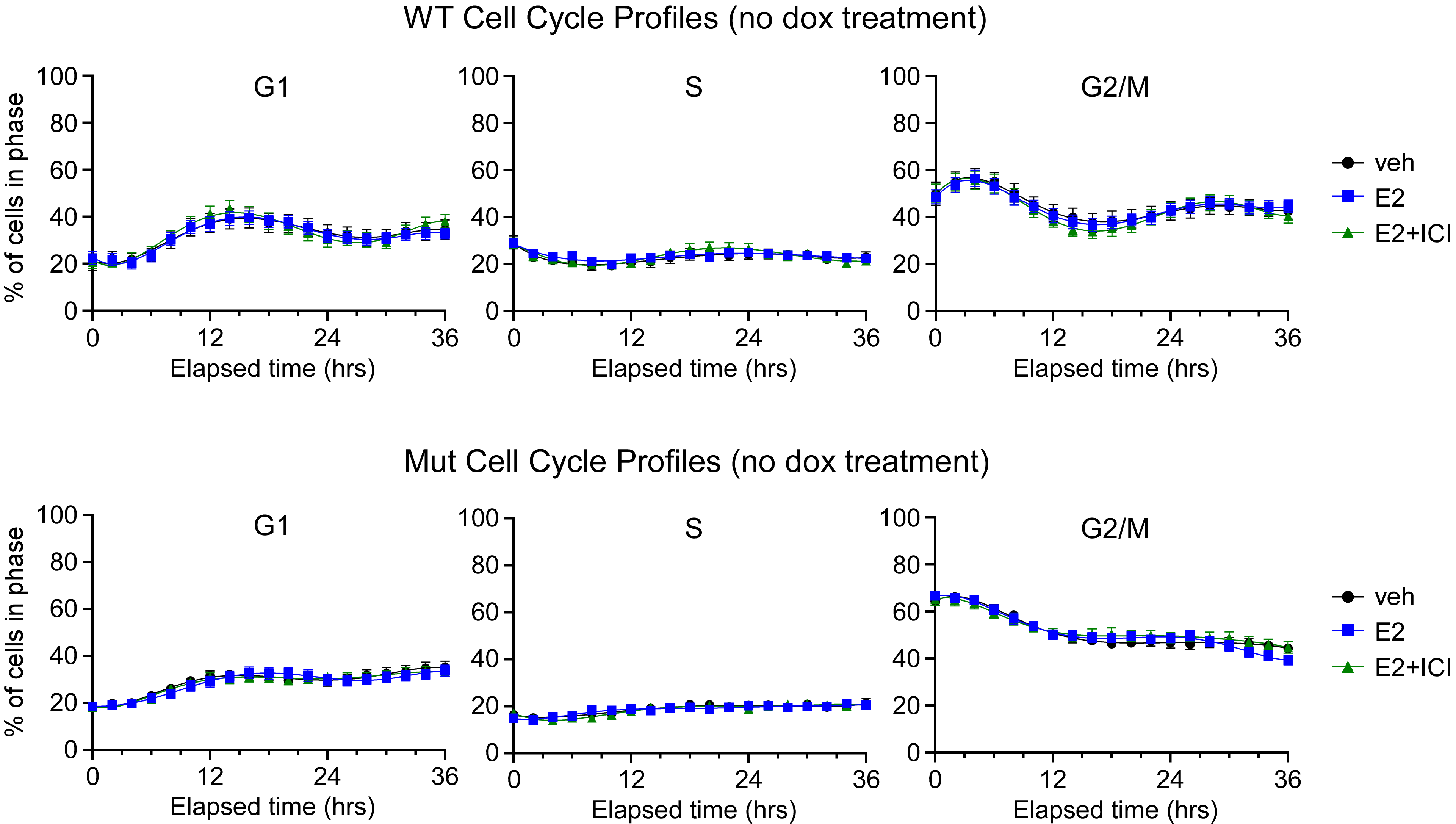

Supplement: SUPPLEMENTARY FIGURE 2 — Cell cycle analysis of WT and ERβDBD-Mut MDA-MB-231 cells in the absence of dox exposure. Real-time assessment of ERβ WT and Mut MDA-MB-231 cell cycle progression in the absence of dox exposure (i.e., absence of ERβ expression) following veh, E2, or E2+ICI treatment. No significant differences were detected (ANOVA). [file Image_2.TIF]
