# Supplementary material for: Disruption of estrogen receptor beta’s DNA binding domain impairs its tumor suppressive effects in triple negative breast cancer
Source: Front Med (Lausanne). 2023 Feb 28;10:1047166. doi: 10.3389/fmed.2023.1047166 (PMC10011152; doi:10.3389/fmed.2023.1047166)
Supplement: SUPPLEMENTARY TABLE 1 — Sequences of primers used for RT-qPCR. [file Table_1.pdf]

SUPPLEMENTAL TABLES

Supplemental Table 1. Sequences of primers used for RT-qPCR.

Gene	Forward Primer (5'-3')	Reverse Primer (5'-3')
ALOX5AP	TGTAGATGCGTACCCCACTT	AGTATGATGCGTTTCCCAAA
CSF2	CCATGATGGCCAGCCACTA	GGTGATAATCTGGGTTGCACA
CST1	AAGAGCCAGGCCAACAGACCGTTT	AGAGCACAACTGTTTCTTCTGCAGT
CST5	AGTACTACAGCCGCCCTCTG	GGTTCGACCGAACTTCACAT
CXCL2	TTGCCACCACCTATTAGCCA	TGTGTGGCAAGGACCTCTAG
ERβ	GATAAAAACCGGCGCAAGAG	TCACCATTCCCACTTCGTAACA
HPRT1	CGTCTTGCTCGAGATGTGATG	GAGCACACAGAGGGCTACAATG
IL11	ATCCCCCCGCCATTATCTC	CCACCCCTGCTCCTGAAATA
IL1A	TGCTGAAGGAGATGCCTGAGA	CCCAGAAGAAGAGGAGGTTGGT
IL1B	CCACTACAGCAAGGGCTTCAG	GAACCAGCATCTTCCTCAGCTT
IL8	GACAAGAGCCAGGAAGAAACCA	GGCCAGCTTGGAAGTCATGT
LCN2	CAAGGAGCTGACTTCGGAAC	TACACTGGTCGATTGGGACA

Supplemental Table 2. Antibodies used for western blotting (WB) and immunoprecipitation (IP) including the company of purchase, catalog number, and dilution of primary antibody used in indicated assay.

Protein	Vendor and Number	Application	Dilution(s)
ERβ	R&D Systems PPZ0506	WB	1:500
EZH2	Cell Signaling 5246	WB	1:1000
FLAG	Sigma F3165	WB, IP	1:1000, 10µL/IP
NFκB (p65)	Cell Signaling 8242	WB	1:1000
β-actin	Sigma A2228	WB	1:10,000

Supplemental Table 3. Sequences of primers used for ChIP-PCR.

Site	Forward Primer (5'-3')	Reverse Primer (5'-3')
ALOX5AP	TGGCCCTCTGACATTGCACTGCC	GGAATCAGGCCATGCAGAGCTGC
CST 1	CCAGAGATTCAGAGACCGTGACC	CTTCCCCAGTGCTCCCAAGCTTG
CST5	CAGCTCCCTGCCCCAGGAATTC	GGATTCCAGAGAGGTGACACCG
CXCL2	AGGCTCCTTCTGTTATTATCTTCATC	CTTGCTAATATGGAACAGGGAATGT
IL11	GGCTCTGAGGCTCTGTGCAGAC	AGAGCTCTTACCTGAGGCGATGAG
IL1B	TTTGAGACAGGGTCTTGCTTTGTTG	GTGGAGGCACACACCTCTAGTCC

Supplemental Table 4. Genes significantly regulated following 5 days of 1nM E2 treatment of WT ERβ expressing MDA-MB-231 cells.

Gene	logFC	logCPM	PValue	FDR
F13A1	9.078871209	4.86423098	0.00E+00	0.00E+00

CST5	9.067382589	4.963977153	0.00E+00	0.00E+00
CXCL14	8.448357823	7.035281536	0.00E+00	0.00E+00
CD300LF	8.400976353	-0.751746467	6.47E-34	7.65E-33
CST2	8.380197392	6.375926366	0.00E+00	0.00E+00
SYT8	7.423114271	-1.615492284	9.75E-19	6.05E-18
LOXL4	7.415968031	11.5068863	0.00E+00	0.00E+00
CST1	7.322542613	10.25608819	0.00E+00	0.00E+00
SERPINA9	7.306550565	5.336188071	0.00E+00	0.00E+00
ARHGAP36	7.300585094	0.007908911	2.54E-59	5.75E-58
CADM1	7.245826786	3.053727279	2.01E-313	5.11E-311
PRSS58	7.241076965	-1.770033158	8.08E-17	4.54E-16
KRT13	7.117063284	6.566489747	0.00E+00	0.00E+00
MGAT3	7.09891991	2.908921626	2.00E-270	4.18E-268
TMOD1	7.08294228	5.340348238	0.00E+00	0.00E+00
PRB4	7.076224229	-1.901131772	2.91E-14	1.40E-13
ACSM1	6.969712983	-1.992336749	7.81E-14	3.63E-13
CYTH4	6.948277338	4.301953264	0.00E+00	0.00E+00
CPZ	6.838485201	2.25879575	2.25E-198	2.92E-196
SLC2A5	6.816846637	4.091677753	0.00E+00	0.00E+00
CCN5	6.75167616	3.475652223	2.08E-318	6.08E-316
TFF1	6.646238461	-0.606270676	9.76E-31	1.02E-29
RGS7BP	6.587822123	0.577143604	4.41E-83	1.55E-81
TNS4	6.563547301	5.463803194	0.00E+00	0.00E+00
CST4	6.501202668	9.014697715	0.00E+00	0.00E+00
CACNA1B	6.421228234	1.997673245	1.81E-181	2.05E-179
GASK1B	6.320599303	5.021490905	0.00E+00	0.00E+00
MAB21L4	6.163196845	1.443543013	1.17E-117	6.79E-116
ATP10B	6.139805219	0.147063695	1.05E-47	1.80E-46
DPYSL3	6.034288232	1.7522773	1.89E-125	1.25E-123
TUBA3E	6.031685095	2.772115717	1.30E-249	2.39E-247
TMPRSS3	6.00712079	2.553583052	1.99E-233	3.22E-231
ABCB1	5.905607132	1.356954498	3.44E-121	2.13E-119
IGFBP5	5.872616029	2.3528883	2.45E-205	3.34E-203
B3GNT6	5.850530029	1.446689187	8.10E-135	6.09E-133
CHIA	5.719893013	-1.424754204	3.15E-19	2.02E-18
FGFBP1	5.685348733	-0.743167401	1.27E-30	1.31E-29
ACKR3	5.616182392	3.183553991	4.78E-267	9.54E-265
INHBB	5.590211904	5.855631676	0.00E+00	0.00E+00
LUM	5.57045187	-0.846852082	6.01E-29	5.79E-28
GJA5	5.424671679	1.165029317	2.46E-81	8.37E-80
GCNT3	5.374305362	2.30746382	3.04E-171	3.30E-169
ALPG	5.307381322	3.624096716	1.38E-305	3.33E-303
FGG	5.304348423	-0.617929877	8.11E-33	9.22E-32
ALPP	5.297696386	7.002581964	0.00E+00	0.00E+00
RET	5.288536228	0.005303801	1.83E-39	2.49E-38
B4GALNT2	5.278482951	3.019766698	3.07E-266	6.04E-264
OLFML3	5.265599506	3.358813662	8.24E-245	1.42E-242

HAVCR2	5.256366642	5.534070481	0.00E+00	0.00E+00
ELF3	5.255442491	6.571534814	0.00E+00	0.00E+00
SERPINA6	5.239261108	1.320859036	6.95E-99	3.14E-97
GJA1	5.197535812	6.571876521	0.00E+00	0.00E+00
AFF2	5.13325705	4.243032872	0.00E+00	0.00E+00
RBP3	5.071544881	-1.285938536	1.14E-20	7.86E-20
CFH	5.064483023	0.033464972	6.48E-36	8.07E-35
COMP	5.060854199	-1.968974505	1.13E-12	4.80E-12
S100A7	5.008335564	-1.344390785	2.78E-18	1.68E-17
MUC16	4.992674206	0.632121034	9.00E-70	2.57E-68
PLEKHS1	4.973843118	2.633646726	2.52E-209	3.51E-207
DHRS9	4.960220491	-1.378125224	1.48E-18	9.11E-18
DNAJC12	4.837713639	0.191992093	4.59E-54	9.34E-53
FRK	4.820486683	1.347248193	3.47E-72	1.02E-70
KRT23	4.813461935	-1.507000194	6.97E-16	3.71E-15
LAMC2	4.783819905	9.511913209	0.00E+00	0.00E+00
NAT8	4.776274471	0.131289604	1.23E-43	1.89E-42
SOD3	4.760375594	0.275102856	1.11E-54	2.29E-53
SLCO2B1	4.758986625	-0.057077306	3.75E-44	5.81E-43
KLRC4	4.72176985	-0.289291278	1.74E-38	2.33E-37
TFF2	4.711285639	2.243186832	2.58E-157	2.46E-155
COL21A1	4.690155461	0.989512612	6.42E-56	1.36E-54
FMOD	4.666288181	5.227428768	0.00E+00	0.00E+00
RETREG1	4.62090557	3.260447538	2.94E-241	4.94E-239
SEMA3B	4.599468037	8.01826836	0.00E+00	0.00E+00
DAPP1	4.59609903	1.134868063	5.85E-97	2.56E-95
FRAS1	4.553599307	6.649791151	0.00E+00	0.00E+00
VASN	4.526493283	7.103018321	0.00E+00	0.00E+00
MYH15	4.499120098	2.181580441	5.57E-149	4.79E-147
SYT1	4.478542822	3.021034811	3.89E-221	5.88E-219
CALHM5	4.468081071	0.007411626	3.51E-40	4.89E-39
PLA2G4D	4.456579218	3.902589823	1.04E-319	3.12E-317
PDZK1	4.383734846	4.971015891	0.00E+00	0.00E+00
RXFP2	4.377292848	-0.825092173	1.12E-25	9.57E-25
IL1R2	4.350251436	1.904121343	1.60E-137	1.25E-135
RAB37	4.344355907	4.208708574	4.73E-215	6.85E-213
CACNB4	4.344209886	1.706436228	4.41E-114	2.45E-112
SERPINA12	4.322722813	-0.450808578	1.59E-30	1.64E-29
CRISP3	4.320837222	-1.473724405	3.22E-16	1.75E-15
PLET1	4.313877737	1.406795418	6.33E-102	2.98E-100
CALB2	4.274703907	8.19646034	0.00E+00	0.00E+00
FAXDC2	4.259624257	5.572681763	0.00E+00	0.00E+00
TH	4.244391389	-0.519958072	1.52E-30	1.56E-29
CDH1	4.23901861	5.136669996	1.26E-313	3.28E-311
IGF2BP1	4.22510767	1.821751446	2.77E-120	1.69E-118
NXPH3	4.210156303	3.224513409	5.42E-236	8.99E-234
ITGB8	4.200035033	3.806112227	6.66E-277	1.48E-274

SPTA1	4.188951921	-0.401316292	1.98E-30	2.02E-29
TNFSF14	4.186155296	1.75328814	7.77E-101	3.60E-99
CEMIP	4.182888581	8.99451081	0.00E+00	0.00E+00
NAP1L3	4.168332571	0.939238436	1.73E-77	5.58E-76
MMP12	4.154668437	4.214490259	0.00E+00	3.15E-306
KCNK15	4.154084569	2.279105126	1.23E-162	1.24E-160
KLRC2	4.151172194	4.120108224	0.00E+00	3.61E-307
C1orf116	4.119528495	5.046682136	0.00E+00	0.00E+00
PADI4	4.110196706	2.162148836	2.95E-116	1.69E-114
SLC6A11	4.072459948	-1.67183161	1.72E-12	7.23E-12
CLEC4M	4.068578279	3.500520324	2.19E-184	2.53E-182
LYZ	4.044881422	0.116544062	1.65E-35	2.03E-34
NCAM2	4.039476015	3.909520927	4.21E-232	6.74E-230
NBPF6	3.980778154	1.565302794	4.51E-66	1.19E-64
CD300C	3.950003321	3.243079333	5.48E-184	6.28E-182
KLRC3	3.942761385	2.810882881	4.15E-153	3.78E-151
NBPF4	3.929753699	1.105137795	4.68E-60	1.09E-58
OR2L13	3.923056289	-1.214123251	4.50E-18	2.70E-17
CRISPLD2	3.918292347	6.976626599	0.00E+00	0.00E+00
CSRNP3	3.908631744	1.382565744	5.01E-98	2.22E-96
UBD	3.908064053	-0.116771087	7.30E-37	9.32E-36
OR10H1	3.899919148	-1.812589411	9.91E-12	3.93E-11
SPATA46	3.898677462	0.25643508	6.63E-41	9.43E-40
MARCO	3.888621141	-1.242258629	3.22E-17	1.85E-16
CALCR	3.886326128	-1.506784919	1.36E-14	6.68E-14
ITGAL	3.878207282	0.6097788	4.69E-56	9.97E-55
PRSS23	3.871246186	10.78542138	0.00E+00	0.00E+00
SFTPD	3.8685164	1.264029594	1.11E-74	3.36E-73
S100A9	3.857147746	-1.270321285	3.27E-16	1.77E-15
GPR78	3.823676623	-0.304966916	5.52E-31	5.81E-30
PTPRQ	3.82077086	-1.876221942	5.40E-11	2.02E-10
OTOF	3.818963161	0.18747794	5.33E-46	8.65E-45
RERGL	3.81734586	-2.284407856	3.62E-08	1.09E-07
HECW1	3.811193367	3.43287961	4.97E-207	6.84E-205
LEXM	3.809043122	0.480737722	5.03E-55	1.05E-53
BEST1	3.790781359	2.380796702	5.17E-137	3.93E-135
DAPK2	3.772817301	4.861586376	5.19E-318	1.49E-315
SYT16	3.749981522	-0.370858104	6.04E-27	5.40E-26
LSMEM2	3.737428204	2.416143699	4.04E-136	3.05E-134
PCDH18	3.733388841	1.95868901	6.43E-82	2.21E-80
CYP26B1	3.706422072	8.161788639	0.00E+00	0.00E+00
SYBU	3.691724248	2.879525246	5.71E-172	6.23E-170
ATP4A	3.689497209	-0.014491778	4.54E-39	6.13E-38
TRPM8	3.677331182	-1.671483797	5.38E-12	2.19E-11
ABCG2	3.659924045	3.343131962	4.15E-158	4.02E-156
SOX6	3.645550327	-0.126696486	1.27E-20	8.68E-20
ADAM29	3.620867106	0.243952647	4.22E-43	6.38E-42

SEMA3A	3.619812118	2.453487292	3.11E-120	1.88E-118
WDFY4	3.618470173	0.563725438	4.99E-31	5.26E-30
GRIK2	3.594103035	2.572724794	3.99E-120	2.40E-118
CYP19A1	3.584262622	-1.490882466	3.50E-13	1.55E-12
PCDH20	3.563976212	-1.507266516	6.61E-13	2.87E-12
SLCO2A1	3.536434122	1.504622209	1.63E-75	5.12E-74
LGR5	3.536261033	-0.813793024	9.66E-21	6.66E-20
FRRS1	3.513608159	0.765989128	1.37E-37	1.78E-36
IVL	3.469141956	-1.833855272	1.27E-10	4.65E-10
AGT	3.458464014	-0.74282667	1.87E-20	1.27E-19
TTC22	3.452889684	1.796394944	4.74E-105	2.31E-103
GREB1	3.44197149	4.528692363	7.56E-268	1.53E-265
ADGRF4	3.434150742	-1.613089432	5.87E-12	2.38E-11
TNIP3	3.424820127	1.313019239	1.64E-59	3.74E-58
DIO2	3.404864849	2.817674667	7.69E-114	4.22E-112
SPATA31D4	3.40486002	2.007704515	1.31E-99	5.96E-98
SPATA31D3	3.399030291	1.910339646	2.83E-104	1.36E-102
C10orf90	3.383850753	0.947328765	1.64E-61	3.98E-60
RASGRP1	3.362126141	2.67253908	3.55E-116	2.02E-114
ANGPTL2	3.348669744	5.493645624	0.00E+00	0.00E+00
FAM83A	3.347177204	6.088318057	0.00E+00	0.00E+00
LRGUK	3.330568584	-1.132632963	3.92E-16	2.12E-15
PAG1	3.316202761	2.576361154	2.77E-120	1.69E-118
SDK1	3.313897918	4.695586134	2.67E-241	4.54E-239
NAALADL2	3.310950929	0.106031287	4.93E-38	6.50E-37
IGSF1	3.290627152	-1.968930179	4.06E-09	1.32E-08
CPM	3.286707315	5.252369641	5.25E-317	1.42E-314
SERPINA1	3.281729714	8.570091624	0.00E+00	0.00E+00
CLIC5	3.28080883	3.830928774	1.14E-197	1.45E-195
SDR16C5	3.278545364	-0.370789043	1.39E-27	1.27E-26
PDGFB	3.256172638	4.693117766	2.46E-268	5.06E-266
CDC42BPG	3.236923858	3.473781042	1.32E-169	1.36E-167
DACT2	3.232882867	-1.21284113	2.39E-14	1.15E-13
FAAH2	3.226754306	-2.016492092	1.38E-08	4.31E-08
BMP2	3.223754449	0.688402249	4.08E-49	7.36E-48
LILRB1	3.22278602	-2.017302162	1.60E-08	4.95E-08
TPD52L1	3.222274922	3.95156968	1.79E-200	2.37E-198
HTR2C	3.214587816	-1.226928077	4.00E-14	1.90E-13
CP	3.21330701	-0.511661567	8.07E-21	5.58E-20
EPHA4	3.209521556	2.609201721	2.76E-134	2.03E-132
CCDC80	3.151710832	7.970737937	0.00E+00	0.00E+00
AFF3	3.126683832	2.370476269	7.72E-112	4.14E-110
C3	3.117849951	10.27520686	1.05E-303	2.48E-301
C5AR2	3.114812297	-1.855381613	1.86E-09	6.24E-09
RHOJ	3.112783577	1.410686667	6.89E-50	1.28E-48
RNF223	3.110542123	1.809064616	1.31E-50	2.47E-49
GPER1	3.09926902	2.392436836	6.18E-96	2.65E-94

MALL	3.089249756	4.760806418	1.59E-196	1.98E-194
DPP4	3.08336244	4.592036923	4.16E-202	5.61E-200
PAPSS2	3.070409635	10.24638679	0.00E+00	0.00E+00
TP63	3.065337365	-0.173227973	3.19E-26	2.78E-25
TENT5A	3.059864615	3.963417766	6.89E-123	4.39E-121
FCGR1A	3.051271114	-0.489499272	1.07E-16	5.95E-16
C6orf58	3.051260026	-2.143695632	2.14E-07	6.02E-07
SMAD9	3.050467226	4.794878212	2.37E-156	2.23E-154
PCDHGA3	3.044993843	-0.403099638	1.60E-24	1.30E-23
PDZK1IP1	3.044934531	4.51332878	1.21E-230	1.91E-228
CNNM1	3.044593537	-1.361368133	2.69E-12	1.11E-11
FBXL7	3.03941643	2.222429324	1.53E-96	6.60E-95
ACKR2	3.033881899	-0.866412056	4.09E-17	2.34E-16
COL17A1	3.021678625	8.563777874	0.00E+00	0.00E+00
SCN8A	3.018515983	-0.870857404	9.90E-13	4.24E-12
OPTC	3.008771929	-1.243374847	2.55E-12	1.06E-11
PLCB1	3.003824183	-0.79083452	4.36E-16	2.34E-15
LRRTM3	2.996627356	-1.944432332	2.49E-08	7.60E-08
GM2A	2.992360621	7.990993706	0.00E+00	0.00E+00
CTSO	2.980698541	3.080414491	1.66E-140	1.32E-138
BIRC3	2.978795501	8.449222953	4.10E-194	4.95E-192
SPP2	2.976694536	-1.576420985	1.17E-09	3.98E-09
CHAT	2.970212737	-1.423148107	1.39E-11	5.46E-11
LGALS9	2.969976087	2.615861504	5.38E-110	2.81E-108
SLAMF7	2.968480327	-0.466868496	1.54E-22	1.16E-21
PTH1R	2.961129786	1.818644279	5.18E-90	2.03E-88
CPN2	2.942204299	0.921552282	5.68E-54	1.15E-52
ISM1	2.941277993	0.892476365	2.10E-38	2.80E-37
FGD3	2.931368187	0.625354926	7.69E-35	9.30E-34
FABP3	2.928060339	1.83635353	2.58E-83	9.12E-82
ANOS1	2.921667756	7.601691201	8.19E-112	4.37E-110
NOL4L	2.907073057	5.448460388	9.31E-273	2.00E-270
GSN	2.901499311	8.761961401	1.31E-294	3.00E-292
KLRK1	2.900942612	-1.081746708	1.18E-13	5.41E-13
KLRK1	2.900942612	-1.081746708	1.18E-13	5.41E-13
PROM2	2.89024528	1.209781472	1.38E-50	2.58E-49
IL2RG	2.868329853	2.392973396	9.85E-95	4.13E-93
DTNA	2.864945745	-1.096134769	2.60E-09	8.65E-09
KDR	2.859855623	2.587502083	2.27E-122	1.44E-120
SEMA5A	2.84692263	5.333931552	6.17E-134	4.52E-132
LRG1	2.843181473	2.759612059	2.00E-112	1.08E-110
RUNDC3B	2.834644915	-0.741085175	7.32E-18	4.35E-17
ARMH4	2.829510443	3.618234873	1.02E-151	9.10E-150
SCUBE1	2.827168045	-0.502355029	8.73E-16	4.63E-15
DSEL	2.826126231	4.594654905	4.05E-198	5.22E-196
CD22	2.824895761	4.348758245	5.12E-199	6.72E-197
CTNNA2	2.814419694	0.663312631	1.06E-30	1.10E-29

HAS3	2.807273156	3.743867264	9.82E-148	8.29E-146
PDK4	2.804285048	1.893452229	5.55E-54	1.13E-52
IGFBP4	2.802048825	11.66839337	1.06E-262	2.05E-260
CA9	2.792638217	1.503207563	4.49E-46	7.32E-45
SLC6A9	2.789301026	6.541726935	8.19E-201	1.09E-198
CDH2	2.77293295	5.076806869	3.25E-170	3.39E-168
RUBCNL	2.772746772	1.558134016	1.62E-63	4.04E-62
HKDC1	2.763014553	5.008618626	2.36E-247	4.22E-245
CD83	2.760508807	5.796807641	5.74E-271	1.22E-268
SAMD9	2.753358729	3.450392218	4.00E-112	2.16E-110
OTUB2	2.750772203	6.130073793	4.90E-234	8.02E-232
ADAMTSL5	2.746050772	5.426599284	1.09E-167	1.11E-165
BANK1	2.740259504	2.143552535	1.19E-86	4.45E-85
RGL1	2.722087862	4.011946353	9.16E-171	9.78E-169
CISH	2.720968044	2.787813396	3.16E-88	1.22E-86
GRIN2C	2.719966346	1.132848224	1.67E-42	2.48E-41
SUSD2	2.694633198	1.110817559	2.90E-48	5.03E-47
ROR2	2.692549866	0.594798494	9.44E-28	8.72E-27
EPB41L1	2.672413014	6.720946868	6.72E-320	2.06E-317
FMO4	2.671804444	-0.959097803	8.50E-14	3.93E-13
TMEM266	2.651124523	0.315510193	2.56E-33	2.97E-32
SLC4A10	2.649911164	-0.450026077	4.51E-20	3.00E-19
CPE	2.64516274	3.12469446	4.12E-97	1.81E-95
PPP4R4	2.633291717	2.055854743	1.03E-69	2.93E-68
RGS9	2.627909805	3.261927888	2.76E-143	2.25E-141
ISM2	2.623569792	-1.091933461	1.80E-12	7.53E-12
SPTBN5	2.611256571	3.273878492	1.52E-110	8.01E-109
TGM2	2.607185069	12.32396775	1.44E-316	3.82E-314
CPA4	2.603537849	6.725750959	1.85E-317	5.11E-315
BSN	2.589334278	1.165064092	1.61E-50	3.02E-49
NCMAP	2.58666786	-1.709604022	1.35E-08	4.21E-08
CLEC4O	2.584983354	-1.710218307	2.62E-08	7.97E-08
CD52	2.584953203	-1.120293215	1.91E-11	7.41E-11
FPR2	2.580742656	0.136772665	1.30E-24	1.07E-23
FFAR4	2.579305911	-2.040849372	9.87E-07	2.60E-06
PIFO	2.576008881	-2.041550172	1.12E-06	2.93E-06
ZNF521	2.574936462	-1.578363883	2.28E-08	6.99E-08
ADAM12	2.574082132	3.474456749	6.94E-138	5.46E-136
KLF8	2.571042777	-0.236029496	6.76E-18	4.03E-17
AQP3	2.564634439	3.320724552	1.79E-128	1.22E-126
ADGRD1	2.556587093	4.712013763	2.33E-193	2.79E-191
MARCKSL1	2.54421312	6.084650954	3.81E-245	6.64E-243
TNFRSF11A	2.541953673	2.187032319	6.39E-81	2.15E-79
FRMPD3	2.53640695	2.728954344	6.70E-86	2.46E-84
CT62	2.527747052	6.384336469	1.75E-225	2.71E-223
TGFA	2.526205506	9.185151327	4.05E-124	2.61E-122
ARRB1	2.525370507	6.47104934	5.48E-249	9.93E-247

PRR16	2.517389121	3.018008773	4.58E-102	2.17E-100
KCNN3	2.516466488	1.484594363	2.84E-60	6.66E-59
FAM222A	2.514724699	2.822701172	6.44E-84	2.31E-82
SAMD9L	2.506292457	2.574886912	1.79E-66	4.78E-65
SERPINB6	2.496890055	9.019790403	1.01E-257	1.91E-255
ZNF219	2.475506159	2.839591394	1.31E-48	2.33E-47
LIPH	2.475108111	4.146787549	6.07E-152	5.42E-150
SERPINI1	2.458576441	-0.663128872	5.80E-14	2.72E-13
KIAA1324L	2.454972018	-0.317877304	1.78E-15	9.24E-15
CHI3L2	2.453118241	0.793063635	1.84E-35	2.25E-34
PCDHB10	2.451026119	-1.043327203	8.19E-12	3.27E-11
PLA2G4E	2.446595316	-1.671976181	1.13E-07	3.26E-07
OASL	2.443665556	5.26297073	2.06E-148	1.76E-146
CNTN5	2.442051862	-1.330217861	3.64E-09	1.19E-08
MAOA	2.441972748	7.610874353	2.33E-170	2.45E-168
SLC2A9	2.44101591	-0.440519136	1.15E-15	6.03E-15
THBS2	2.437911659	8.232826738	8.63E-253	1.60E-250
KIAA0513	2.433156618	6.038690932	3.57E-215	5.22E-213
MAG	2.431761695	-1.96935849	3.17E-06	7.88E-06
IRX3	2.430470918	6.658608926	1.00E-132	7.21E-131
CCDC85A	2.422320506	2.081791476	2.83E-55	5.91E-54
NLRP10	2.420000022	0.362639535	8.15E-30	8.13E-29
PORCN	2.418647425	6.26806915	7.87E-221	1.18E-218
PLXNA4	2.411351452	0.851454038	8.38E-39	1.13E-37
GPR132	2.405840056	2.641565651	1.80E-66	4.80E-65
LANCL3	2.396729431	1.354801983	1.04E-42	1.56E-41
ITGB2	2.395801548	3.189687895	8.08E-106	3.99E-104
KLHDC9	2.394080742	-0.653503469	2.03E-15	1.05E-14
KLRC1	2.393168831	-0.181128122	2.60E-21	1.85E-20
HMGN5	2.392557335	-1.091639719	5.16E-10	1.81E-09
AWAT1	2.389494415	-1.487516306	1.13E-06	2.95E-06
NPY1R	2.389358125	-0.140123556	5.61E-19	3.54E-18
NMNAT2	2.386518872	4.171846914	4.03E-139	3.19E-137
OSR1	2.380981673	2.380821433	4.39E-86	1.62E-84
IFI27	2.377168962	6.673938679	4.70E-156	4.40E-154
TUBA3D	2.370431739	1.821089536	2.02E-55	4.24E-54
FCGR1B	2.369038846	-1.285872798	1.10E-09	3.75E-09
MXD1	2.368091631	5.860159311	7.42E-129	5.11E-127
CDON	2.356451709	3.956798781	6.46E-109	3.32E-107
IL1R1	2.354819632	5.952047811	2.22E-93	9.08E-92
YPEL4	2.349416955	3.507384076	2.66E-127	1.79E-125
SLC22A2	2.349355783	-1.877170411	2.89E-06	7.21E-06
ARHGEF37	2.344976556	2.168819356	2.29E-67	6.25E-66
MGAM	2.343608722	4.111168567	1.04E-153	9.54E-152
ERV3-1	2.340960198	2.584947992	2.82E-62	6.88E-61
STS	2.338416782	6.864543973	6.86E-118	4.03E-116
NXF3	2.336989343	-0.76233299	1.12E-13	5.12E-13

HSPB8	2.332558762	7.87080332	1.21E-275	2.65E-273
PRDM1	2.330578781	4.01317342	8.16E-144	6.73E-142
CD34	2.326730483	4.674421357	1.67E-125	1.10E-123
IFITM10	2.324976818	3.514154712	6.85E-97	2.98E-95
SMIM10L2A	2.321099322	-1.899215335	1.76E-06	4.50E-06
SLC6A20	2.320397147	-0.911909404	8.62E-10	2.97E-09
PADI2	2.319818164	0.08047114	5.72E-22	4.19E-21
IGFBP6	2.316661564	4.337798597	1.29E-67	3.55E-66
SLCO3A1	2.310496049	5.165938703	6.63E-179	7.36E-177
HMCN1	2.309563353	0.177620482	7.02E-22	5.11E-21
PCDHGB2	2.30946287	2.650086109	3.33E-77	1.07E-75
OVCH2	2.30510664	-0.722087958	2.74E-13	1.22E-12
S1PR1	2.303518373	2.036247252	8.10E-70	2.32E-68
FAM25A	2.297492897	-1.542323191	5.70E-08	1.69E-07
CSF2RB	2.293992686	3.024623517	7.98E-75	2.45E-73
CCDC159	2.293590268	1.284324713	5.19E-40	7.19E-39
PLAC1	2.287357463	1.965150899	4.44E-43	6.69E-42
WNT7B	2.280388357	2.552139065	2.59E-76	8.20E-75
SPOCK1	2.270099355	-1.456913847	3.61E-08	1.08E-07
SLC38A4	2.262223564	1.869430916	1.20E-24	9.83E-24
CTSD	2.262065806	9.123298415	5.09E-122	3.22E-120
PRLR	2.261719744	0.769220784	3.25E-24	2.63E-23
HFE	2.261256331	5.121773732	4.68E-151	4.13E-149
H2AC6	2.259010514	4.181385749	6.57E-85	2.38E-83
MEF2C	2.255530878	0.098776142	5.74E-21	4.00E-20
RFPL4AL1	2.237372959	1.807733387	8.22E-50	1.52E-48
CCDC68	2.235759526	3.368210529	1.56E-91	6.19E-90
SCN2A	2.234079407	-1.390859574	3.70E-08	1.11E-07
KCNJ18	2.23326435	-0.84468884	3.93E-08	1.18E-07
KRT17	2.229782824	3.744537977	9.03E-118	5.24E-116
LOX	2.228527488	7.867529731	8.47E-144	6.94E-142
RFPL4A	2.226480894	2.032761947	4.53E-49	8.15E-48
SYCP2	2.218417809	-0.664703741	7.57E-11	2.80E-10
LAMA5	2.217115576	8.813075946	2.76E-73	8.25E-72
IQCN	2.217108732	-0.199718732	1.02E-11	4.03E-11
PHETA1	2.211801232	5.515471643	7.26E-119	4.31E-117
TSPAN15	2.204782489	4.954877411	6.85E-160	6.73E-158
COL1A2	2.195577731	3.754794916	6.35E-107	3.18E-105
USP41	2.193087368	0.211273756	1.07E-23	8.46E-23
KCNH3	2.187785632	-0.813881763	1.40E-11	5.49E-11
PRELP	2.18714871	0.322495396	2.77E-23	2.15E-22
NT5E	2.18516451	10.03790639	4.71E-197	5.94E-195
SLC1A1	2.182212806	2.025118477	2.83E-58	6.26E-57
FAM71F2	2.18187726	1.04506636	3.64E-20	2.44E-19
BDH2	2.181854891	3.297151476	1.14E-98	5.11E-97
GPC1	2.181527809	6.181809995	2.85E-103	1.37E-101
PCDHB6	2.181284684	-1.243468706	3.73E-08	1.12E-07

RIPOR2	2.178750283	-1.330582998	1.34E-06	3.49E-06
PCDHB14	2.165065539	-1.344653004	2.92E-08	8.84E-08
PRUNE2	2.165042966	-0.771344136	6.26E-12	2.53E-11
OGDHL	2.162200451	1.857129084	3.92E-50	7.29E-49
FUT8	2.158894688	7.195920489	7.22E-158	6.95E-156
FN1	2.157104752	12.71044424	9.17E-221	1.36E-218
VWA3B	2.156565398	-1.440453549	1.47E-07	4.19E-07
SLC22A23	2.145617912	5.512971428	1.07E-133	7.72E-132
THNSL2	2.139135926	-1.198324062	1.88E-08	5.78E-08
CD38	2.138075994	-1.361892344	2.28E-07	6.39E-07
DLL4	2.137906132	2.737953476	1.87E-71	5.46E-70
MINDY1	2.128870794	4.213900102	5.69E-101	2.65E-99
LRRC15	2.127996053	3.930721346	1.53E-121	9.64E-120
FGD6	2.127166659	6.606022645	6.42E-116	3.64E-114
LGALS9B	2.126367677	0.130639555	6.35E-19	3.99E-18
EHF	2.105415158	2.775522602	5.28E-51	1.00E-49
FILIP1L	2.100048743	5.322919166	3.36E-132	2.41E-130
TCIRG1	2.099659297	7.523950913	6.23E-80	2.08E-78
C1QTNF6	2.096007838	4.972635961	1.65E-134	1.23E-132
PRR29	2.085019124	0.940269979	1.84E-31	1.98E-30
AKAP5	2.082990234	0.241561797	6.36E-22	4.64E-21
ARMH1	2.078736466	-0.615529365	1.10E-09	3.77E-09
CYP21A2	2.078113426	-0.481388789	3.43E-10	1.22E-09
SYT5	2.075669494	0.235256948	8.65E-22	6.26E-21
MR1	2.061038296	4.53581474	3.20E-110	1.68E-108
APBB1	2.059731121	4.568640924	2.57E-144	2.13E-142
MYBPC1	2.058072581	-1.944644963	1.60E-05	3.67E-05
NXNL2	2.052475999	1.16400433	2.68E-30	2.73E-29
PHOSPHO1	2.045901115	0.60107524	7.05E-25	5.85E-24
APOL1	2.045772399	5.330226235	2.55E-147	2.14E-145
ASPRV1	2.044805679	-0.323606125	3.85E-14	1.83E-13
SYNJ2	2.044203465	7.151557651	2.31E-154	2.14E-152
B3GNT7	2.042486569	2.30752589	1.49E-64	3.79E-63
ERVV-2	2.042428027	-0.499920138	2.11E-13	9.51E-13
PTGS2	2.042114317	6.876181828	1.29E-65	3.38E-64
SLC25A42	2.039648201	4.075512119	8.98E-106	4.41E-104
DHFR2	2.035039196	1.672752848	1.71E-32	1.92E-31
CHIC1	2.030785878	3.914263986	6.35E-75	1.96E-73
KLF17	2.03041672	2.987722872	4.53E-89	1.76E-87
GLIPR1	2.023625469	6.120864108	2.49E-124	1.62E-122
TSPAN1	2.022795208	1.315667945	2.26E-38	3.01E-37
GPR37L1	2.020545543	-0.562308087	5.79E-12	2.35E-11
HMGCLL1	2.015234561	-0.568425029	1.98E-11	7.66E-11
LILRB5	2.012383496	-1.853992874	2.50E-05	5.62E-05
HAL	2.009705232	-0.720916715	4.14E-11	1.56E-10
TANC1	2.007825537	6.976880326	1.50E-92	6.11E-91
SDC2	2.006716922	4.144708705	2.47E-78	8.06E-77

SYNE3	1.995356064	5.246022825	1.23E-137	9.59E-136
PLXDC1	1.99475293	-0.534598968	1.01E-12	4.33E-12
TRANK1	1.993358767	3.247350642	4.50E-82	1.55E-80
PIK3CG	1.988639574	3.606137244	6.59E-65	1.69E-63
PODXL	1.988267471	10.48810316	2.73E-246	4.82E-244
GCKR	1.985948336	1.771629065	1.67E-42	2.47E-41
FGF11	1.982897331	2.492829178	1.12E-54	2.32E-53
SYTL5	1.977783931	-1.877573805	2.05E-05	4.65E-05
VWA5A	1.976397646	-0.410683637	2.65E-10	9.48E-10
RMDN2	1.973744582	0.566185428	7.64E-21	5.29E-20
FOLR1	1.97163074	0.432936448	3.33E-22	2.46E-21
PLEKHH1	1.967679816	4.405445563	1.67E-114	9.28E-113
PPIL6	1.962344884	0.940877218	1.63E-31	1.76E-30
SCN3A	1.959928057	-1.490458567	1.94E-06	4.93E-06
SLC46A3	1.95941113	5.694220027	3.44E-130	2.42E-128
EPS8	1.95584543	6.204760642	3.49E-65	9.01E-64
PTAFR	1.954904965	1.249862355	5.01E-29	4.84E-28
ITGA1	1.952493207	4.315220559	4.22E-68	1.17E-66
ACER2	1.949801696	2.803777601	9.89E-51	1.87E-49
ATP2B4	1.949142855	8.700926929	2.53E-162	2.53E-160
GFRA1	1.947517334	-0.173575971	1.42E-13	6.46E-13
SORBS2	1.946082225	2.29263039	1.46E-28	1.39E-27
DAB2	1.94388612	6.906312126	1.59E-146	1.32E-144
IFI44	1.942761003	2.267908415	2.86E-53	5.69E-52
PYROXD2	1.942706006	4.462981642	9.43E-121	5.82E-119
PRDM6	1.941786768	1.507270397	1.48E-34	1.78E-33
RBM47	1.939939476	3.872847656	2.54E-81	8.60E-80
SMOX	1.938931851	6.064159252	5.28E-148	4.48E-146
HPSE	1.937907452	4.571381996	4.57E-112	2.46E-110
PIGM	1.934546918	1.3472839	9.95E-31	1.03E-29
LRP1B	1.933915805	-1.345034616	6.08E-07	1.64E-06
ANXA9	1.933008623	2.723490384	4.92E-59	1.10E-57
CA12	1.930956291	3.931575071	2.53E-87	9.59E-86
COL4A4	1.926406888	2.234370238	9.07E-47	1.51E-45
TSPAN13	1.924284937	4.830842386	1.91E-82	6.63E-81
ACSS1	1.924243183	5.050097126	1.88E-129	1.30E-127
LYPD5	1.920733453	1.091238114	2.84E-24	2.30E-23
CLIC2	1.912404306	-0.846165611	2.46E-09	8.19E-09
CYP2J2	1.912348715	-1.810910189	1.74E-05	3.99E-05
PCDHB11	1.910607527	-1.524024563	1.29E-05	2.99E-05
TPH2	1.909591396	-1.523415646	4.60E-06	1.12E-05
FAT4	1.908877649	5.98294462	1.41E-59	3.23E-58
TP53I3	1.908132159	2.780154642	6.32E-65	1.62E-63
C3AR1	1.907037475	-1.101498092	1.43E-04	2.92E-04
PTPRN	1.905606017	-1.710127292	3.18E-05	7.06E-05
PROS1	1.896810602	3.281606372	1.43E-48	2.53E-47
STXBP6	1.893863711	-1.22745083	9.99E-08	2.89E-07

CRPPA	1.891228605	2.883585572	2.09E-49	3.80E-48
HERC1	1.89090261	7.786246094	3.06E-121	1.91E-119
CDHR2	1.887389675	0.680051938	3.34E-16	1.81E-15
CHGA	1.886399201	-1.081929738	7.19E-06	1.72E-05
MMP24	1.88426343	4.192046012	1.38E-107	6.99E-106
HMGN3	1.884014394	2.699136177	4.12E-66	1.09E-64
HOMEZ	1.882314609	0.945346949	1.11E-20	7.60E-20
SPINT1	1.881594559	7.167341029	6.50E-123	4.16E-121
KCNMB4	1.880125707	1.364790738	1.78E-32	1.99E-31
PBLD	1.879871799	1.563777507	7.12E-41	1.01E-39
TFAP2C	1.879134949	5.836120412	7.72E-126	5.13E-124
CSF1	1.877169315	7.564141999	1.49E-155	1.39E-153
ELFN2	1.877019724	5.551365853	6.35E-110	3.30E-108
USP18	1.877008662	2.619462095	1.21E-63	3.02E-62
PTPRS	1.876808737	6.294911698	7.35E-134	5.35E-132
DRGX	1.871036936	2.347164935	3.85E-60	9.00E-59
NIM1K	1.869484039	0.019796782	9.40E-13	4.03E-12
C1orf226	1.869129817	4.580485291	6.41E-114	3.53E-112
HMOX1	1.867729489	7.175328313	3.03E-181	3.39E-179
ENTPD3	1.866177029	0.101369275	7.24E-16	3.85E-15
ARL4C	1.862849802	7.239778117	6.97E-177	7.68E-175
KRT19	1.860856196	9.524117677	3.98E-95	1.68E-93
GREM1	1.859329278	-1.183946117	1.06E-06	2.78E-06
GNRHR	1.856035434	-1.748454163	2.75E-05	6.15E-05
ARHGAP20	1.850474598	-1.750186616	3.03E-05	6.73E-05
DOK7	1.847725942	-0.248078787	2.42E-09	8.07E-09
ANO1	1.847388094	-0.890106886	1.00E-08	3.17E-08
KIAA0825	1.845578573	-1.329162365	2.65E-06	6.65E-06
CFAP300	1.844046851	0.025850249	3.97E-11	1.50E-10
MAP2	1.843930069	-1.256297486	2.92E-06	7.28E-06
P2RX6	1.839053947	-0.606199731	1.64E-10	5.96E-10
ZNF117	1.837000214	1.800861241	3.38E-17	1.94E-16
LGR4	1.836986841	6.666435838	5.99E-33	6.83E-32
TCN1	1.836060168	2.065159641	3.91E-39	5.30E-38
GDAP1	1.834348678	0.841314207	7.66E-24	6.10E-23
CARD6	1.832305556	4.818075748	2.09E-98	9.29E-97
SRPX2	1.830914012	4.126576192	5.62E-102	2.66E-100
ACSL5	1.830322415	6.458915349	7.35E-159	7.17E-157
DES	1.827701232	-1.256685646	4.54E-05	9.88E-05
PTPRH	1.826349141	6.406064948	9.73E-151	8.53E-149
BICC1	1.821913465	5.46737631	1.40E-76	4.45E-75
SYT12	1.821003038	2.172882682	1.18E-32	1.33E-31
MRVI1	1.81638117	-1.506434937	8.14E-06	1.93E-05
ZNF396	1.81540146	0.168674375	9.80E-18	5.80E-17
IRX5	1.815174283	3.88758197	2.20E-74	6.66E-73
SSH2	1.813996645	6.712325162	5.80E-125	3.80E-123
CASQ2	1.812666354	-1.876567187	5.87E-05	1.26E-04

SCNN1A	1.808287255	-0.024964663	7.45E-13	3.22E-12
IDUA	1.804219852	3.259749286	1.78E-47	3.02E-46
IFIT1	1.803367785	4.865185112	5.19E-72	1.53E-70
LHFPL2	1.801090539	6.657570001	6.56E-81	2.20E-79
ZNF583	1.796635945	2.278493379	3.47E-33	4.01E-32
CNTNAP3	1.796580138	3.892291157	6.34E-69	1.78E-67
HAP1	1.794740191	-0.036294293	3.40E-13	1.51E-12
DENND2D	1.793844615	-0.319737851	6.26E-09	2.01E-08
RASGRF1	1.793279949	4.981624428	7.63E-118	4.45E-116
FDXR	1.787779793	4.901513054	6.66E-67	1.80E-65
CRYBG2	1.787147592	4.5396529	8.64E-97	3.75E-95
PCDHB9	1.786487271	-0.650792114	3.57E-08	1.07E-07
SYTL2	1.78226154	1.228588525	1.22E-31	1.32E-30
DHRS2	1.78009317	1.190938224	2.68E-29	2.62E-28
SH3BGRL	1.778408611	4.059091566	2.24E-68	6.24E-67
ERVV-1	1.7764737	-1.899192091	8.89E-05	1.86E-04
MYO18A	1.776427008	7.184207642	1.04E-108	5.32E-107
FAM13B	1.772894023	4.728533428	2.96E-60	6.93E-59
SEC14L2	1.772046136	5.107144489	6.11E-99	2.77E-97
OTOGL	1.771481281	-0.114435609	1.58E-13	7.17E-13
VNN1	1.770166769	1.676884322	5.80E-26	5.01E-25
TNFRSF19	1.768965993	1.040319792	4.74E-19	3.01E-18
PLCD3	1.768612787	6.841734374	4.13E-126	2.76E-124
TTC9	1.767785068	0.677903135	1.26E-19	8.21E-19
GJB5	1.767527102	-1.540839279	2.19E-05	4.96E-05
STC1	1.765654565	8.719767021	1.05E-169	1.09E-167
KCTD11	1.765200829	5.571363123	8.56E-120	5.12E-118
SLC45A1	1.764415488	1.240288209	8.46E-31	8.82E-30
TREH	1.763579384	-0.826602149	5.79E-05	1.24E-04
CDH3	1.761739621	3.530389278	6.08E-72	1.78E-70
HAO1	1.758042828	-1.066053557	1.61E-07	4.57E-07
ZNF883	1.758037416	-1.811288539	6.36E-05	1.36E-04
SPRY3	1.755562845	-0.946518117	7.68E-06	1.83E-05
SUMF1	1.751592537	3.342538493	1.26E-65	3.29E-64
ARSA	1.74842986	3.281108177	1.31E-52	2.56E-51
PPFIA4	1.746031015	4.019108532	3.14E-77	1.01E-75
FHOD3	1.741955307	5.831352859	1.37E-112	7.46E-111
SLC47A1	1.741099969	-1.728007787	9.06E-05	1.90E-04
SPANXN3	1.739804731	-1.019087102	6.15E-07	1.66E-06
MMP19	1.73974876	3.498431892	5.51E-74	1.66E-72
ZBED6CL	1.739320475	3.95081384	7.42E-51	1.40E-49
ZSWIM5	1.738375631	-0.913619809	8.08E-07	2.15E-06
GBA	1.737712852	7.114519273	3.53E-124	2.28E-122
FER1L6	1.735681807	2.197275594	4.34E-31	4.59E-30
GNGT2	1.734723836	0.919859895	2.19E-25	1.85E-24
MARCHF10	1.728585021	1.761131938	3.21E-34	3.82E-33
RIPK3	1.723920962	-1.650753893	4.64E-05	1.01E-04

FOXD4	1.723263694	-1.65097208	4.80E-05	1.04E-04
CEMIP2	1.722967472	7.90807774	2.29E-75	7.12E-74
RARRES2	1.718946303	1.511248891	3.59E-26	3.13E-25
TNFSF10	1.713325952	3.846350965	1.92E-72	5.66E-71
TEF	1.708639042	3.310251006	2.69E-68	7.47E-67
ECM1	1.708091641	6.758635859	5.64E-103	2.70E-101
FHDC1	1.706014506	2.295536453	2.64E-44	4.11E-43
SPON1	1.705527243	0.320599267	2.48E-15	1.27E-14
SMAD7	1.703705853	4.444193992	1.09E-90	4.29E-89
ANKK1	1.70339269	-1.425441805	4.13E-05	9.03E-05
LRP1	1.703246241	7.475647981	1.52E-95	6.49E-94
PSG8	1.702494471	2.346406863	2.60E-40	3.62E-39
MB	1.701987668	-0.579089704	5.97E-10	2.08E-09
TBC1D2	1.701470188	7.303473732	3.30E-150	2.88E-148
FUT3	1.700872819	-0.750158009	7.58E-09	2.42E-08
COLEC12	1.698941935	-0.465608141	1.13E-10	4.14E-10
TBX15	1.697507877	-0.662216805	1.24E-08	3.88E-08
SATB1	1.695446715	1.025914385	4.25E-26	3.70E-25
SRBD1	1.695073281	5.613619035	1.31E-69	3.71E-68
ZNF608	1.686823453	2.780160282	9.50E-43	1.42E-41
CCNA1	1.683835559	5.635360984	2.62E-105	1.28E-103
TRAM1L1	1.68357288	-0.006412154	1.17E-13	5.34E-13
ISG20	1.682352737	4.759226393	3.35E-56	7.12E-55
PLCZ1	1.681771663	-1.441055633	3.44E-05	7.59E-05
RALGAPA2	1.677769052	4.266004779	9.00E-86	3.30E-84
FANK1	1.677180188	2.170159082	5.21E-40	7.20E-39
GREB1L	1.676737182	6.459470944	4.00E-81	1.35E-79
NCF2	1.676655953	5.098090731	1.22E-99	5.54E-98
ADGRF5	1.675471478	8.782653617	3.68E-86	1.36E-84
PCDHB16	1.675356141	0.571760967	5.29E-19	3.35E-18
FBLN1	1.673245353	0.252866113	2.70E-16	1.47E-15
OSTM1	1.673134139	5.594812515	4.57E-57	9.89E-56
LDLRAD4	1.672093555	-0.523554178	2.96E-08	8.98E-08
CYP1B1	1.66890749	2.917021296	2.25E-35	2.75E-34
HIGD1A	1.667345881	7.038194878	1.14E-114	6.39E-113
ABCA5	1.666803151	2.779688745	2.35E-44	3.68E-43
JAK1	1.664623509	9.336495031	1.22E-64	3.11E-63
DOP1A	1.663158148	2.753771651	4.86E-37	6.23E-36
MIEF2	1.663122542	5.600826392	7.94E-94	3.28E-92
PLOD2	1.660364612	8.748471386	2.61E-47	4.43E-46
JUP	1.660319762	6.678408958	7.60E-94	3.15E-92
MRGPRX4	1.659978744	-0.650781555	4.20E-08	1.25E-07
LYRM9	1.659429543	1.14805576	1.94E-27	1.76E-26
CAPN3	1.659308191	0.000679921	5.66E-13	2.47E-12
DBH	1.65661953	0.46748083	3.83E-16	2.07E-15
TIAF1	1.65651546	-1.612554771	9.46E-05	1.98E-04
CCDC71L	1.656001039	5.912666327	7.89E-105	3.82E-103

ST3GAL5	1.654461633	2.259613299	1.81E-35	2.22E-34
BMP6	1.650229301	0.926333379	9.12E-24	7.24E-23
NRXN3	1.646589965	6.555461585	3.99E-69	1.12E-67
CFAP44	1.643077107	1.938394159	1.00E-27	9.22E-27
SDC4	1.642808902	9.205154922	7.12E-157	6.76E-155
MANEAL	1.641445692	5.380144302	5.88E-85	2.14E-83
TNFRSF9	1.6412084	3.469885695	1.15E-55	2.44E-54
ULK1	1.640403074	6.098413247	6.50E-66	1.71E-64
GJB3	1.639103743	5.238470378	5.32E-59	1.19E-57
KCNJ12	1.637691706	-0.400078743	9.38E-08	2.72E-07
UPK2	1.637357157	-0.402410587	1.16E-09	3.95E-09
FAM102A	1.634150946	5.710133503	8.22E-83	2.87E-81
GNPTAB	1.633742016	5.986984414	1.07E-107	5.44E-106
NUP62CL	1.632506289	-1.283252941	1.99E-05	4.51E-05
CLMP	1.62554247	5.986696838	1.82E-92	7.33E-91
PRRG2	1.624826038	1.408584261	1.27E-17	7.48E-17
PCDH7	1.624672194	-1.344605682	1.24E-05	2.89E-05
DCBLD1	1.624424767	5.049921481	2.84E-86	1.05E-84
PIK3IP1	1.621870627	3.671193201	9.99E-52	1.93E-50
CTSZ	1.621509887	8.633491509	2.52E-130	1.79E-128
TUBA8	1.621224736	0.38000579	1.27E-12	5.38E-12
ENTPD8	1.619928658	-0.523739566	4.14E-08	1.24E-07
ABAT	1.619338943	1.355513764	4.15E-29	4.02E-28
DEPP1	1.615979834	3.058356929	2.35E-54	4.84E-53
BICDL1	1.614174446	2.988088581	5.96E-46	9.63E-45
MUC22	1.614070632	-1.811275255	2.22E-04	4.44E-04
UTS2B	1.610857707	0.184257751	3.27E-14	1.56E-13
CASTOR3	1.610460619	4.022716712	4.32E-74	1.30E-72
HSPA2	1.609395494	2.102840357	7.14E-38	9.36E-37
ARHGAP24	1.608170011	-1.812664636	3.59E-04	7.01E-04
LBH	1.605895998	2.390292226	1.89E-40	2.65E-39
DDIT4L	1.604546975	1.362313204	1.87E-15	9.70E-15
TCN2	1.60419604	0.389787149	5.16E-13	2.26E-12
TACR2	1.604139932	0.109971573	2.91E-14	1.40E-13
OAS1	1.603902262	1.274193971	9.81E-25	8.09E-24
RASGRP3	1.603184233	1.375282159	1.03E-19	6.79E-19
VSIG1	1.602678533	-0.331847	2.00E-08	6.16E-08
PEX11B	1.600475516	2.862745093	3.95E-51	7.54E-50
PKNOX2	1.600464113	-1.424464461	5.18E-05	1.12E-04
MUC21	1.599883836	-2.116942265	2.50E-03	4.34E-03
CST3	1.597648039	7.161821217	1.69E-79	5.61E-78
MUC4	1.596954468	1.42725854	5.65E-15	2.84E-14
TMIE	1.595972927	1.325851093	2.62E-21	1.85E-20
TBXA2R	1.595849291	2.721489934	1.50E-37	1.95E-36
MGAT5B	1.594860881	4.937030855	1.81E-57	3.95E-56
METTL25	1.594301258	0.64584157	2.37E-17	1.37E-16
ZFP3	1.594221234	1.02148192	3.02E-13	1.34E-12

CDKL5	1.593014215	0.839248318	5.33E-19	3.37E-18
NPY4R	1.591455827	0.989178439	2.27E-19	1.46E-18
BDKRB2	1.590967121	-1.240523272	5.27E-05	1.14E-04
DIXDC1	1.58930037	4.18218666	4.32E-52	8.38E-51
RBP7	1.588920938	-0.338415627	2.36E-10	8.50E-10
RBM20	1.588906952	-0.301902867	1.25E-08	3.92E-08
SLC9A3R1	1.588832406	7.274384319	7.53E-77	2.41E-75
ADAMTS3	1.587112137	1.270884718	2.58E-23	2.01E-22
PBX1	1.585194204	0.020778297	1.48E-08	4.59E-08
ACTBL2	1.585069246	-0.308723478	8.33E-10	2.87E-09
MT1F	1.585003513	1.728879081	2.50E-24	2.03E-23
NWD1	1.584879478	-0.279132721	2.61E-10	9.36E-10
CD9	1.582102052	8.027077069	8.10E-113	4.42E-111
ITPK1	1.581787481	8.038301266	1.19E-120	7.31E-119
ZNF552	1.580982842	2.976719814	3.23E-54	6.62E-53
LYPD8	1.580503229	2.510261578	1.49E-23	1.17E-22
GIPR	1.578545233	1.407016082	6.96E-24	5.56E-23
TRPV1	1.578026823	3.676456817	4.43E-52	8.59E-51
GBP4	1.576862086	-1.376393407	2.79E-05	6.23E-05
JAZF1	1.575271825	3.896743989	2.47E-46	4.06E-45
STOX2	1.573541443	0.798024284	1.92E-18	1.17E-17
SERINC2	1.569938843	7.660285901	3.28E-84	1.18E-82
SPIRE2	1.569927207	4.83141837	5.29E-70	1.52E-68
SEC14L6	1.568816086	1.757177601	1.41E-25	1.20E-24
FBXL2	1.568124051	2.885497213	3.20E-45	5.10E-44
NBEA	1.565420666	2.624695364	5.77E-40	7.93E-39
ARSD	1.563580343	4.525981867	7.79E-84	2.79E-82
ABCA7	1.562073698	5.553710937	8.45E-60	1.95E-58
TLR1	1.560444152	-0.20924147	1.12E-09	3.81E-09
LTB	1.558629791	2.901661078	8.46E-25	7.00E-24
MANSC1	1.556001439	2.851132252	1.06E-43	1.63E-42
NUAK1	1.553909408	8.203872379	3.37E-91	1.34E-89
NMRK1	1.549915913	2.837467178	5.22E-40	7.21E-39
PIWIL4	1.548838728	0.533129331	1.69E-11	6.59E-11
KIF21A	1.546328212	4.755540777	3.14E-31	3.34E-30
COL4A5	1.545837519	6.376669446	9.12E-76	2.87E-74
CEP126	1.545570502	2.061134324	1.79E-32	2.00E-31
KCP	1.544134338	0.48059906	1.71E-11	6.66E-11
PBXIP1	1.542386726	5.734283953	4.89E-60	1.14E-58
PTPRE	1.540243925	6.084693878	1.96E-90	7.71E-89
FPGT	1.538325945	2.503965127	2.45E-43	3.72E-42
YPEL2	1.536785361	4.730586529	1.28E-43	1.97E-42
SMIM14	1.53439697	4.106093302	2.65E-41	3.80E-40
CRCT1	1.530202146	-1.614077747	4.08E-04	7.90E-04
WDR63	1.528873667	-1.473643304	1.17E-04	2.42E-04
RTL9	1.52799303	-0.058143855	3.64E-07	1.00E-06
CHURC1	1.526858265	4.011406072	2.83E-62	6.91E-61

GRAMD1C	1.526413544	2.549778623	5.34E-21	3.73E-20
DDX60	1.525785005	4.499466458	7.39E-32	8.07E-31
AAMDC	1.525666136	3.970733074	2.30E-42	3.39E-41
SLFN5	1.524441032	6.795365831	3.89E-58	8.58E-57
ERMAP	1.523531767	3.462321065	8.34E-55	1.73E-53
RAB3B	1.520345753	-0.456854222	1.38E-07	3.94E-07
AKR1C3	1.518583634	2.533743068	3.95E-37	5.08E-36
RNF170	1.518141965	3.865553673	1.71E-62	4.22E-61
FMO5	1.517497568	1.832808924	2.13E-13	9.58E-13
GAS6	1.517181824	6.094639575	2.24E-56	4.78E-55
NPY4R2	1.514710387	0.801098894	9.62E-16	5.08E-15
GSTT2	1.51374352	3.662809062	3.23E-61	7.75E-60
CALCOCO1	1.513336314	5.448821333	4.14E-93	1.69E-91
ST8SIA4	1.513150763	-0.792074602	1.31E-06	3.41E-06
TNIK	1.510975829	4.896070809	9.18E-53	1.81E-51
SLC7A9	1.509696806	-1.877182072	7.62E-04	1.43E-03
TEX9	1.507280922	-1.240093542	1.89E-05	4.31E-05
TET2	1.505648843	4.084802962	1.76E-45	2.83E-44
EFCAB5	1.504653786	0.616945834	9.22E-13	3.96E-12
TMBIM4	1.504371515	4.629176363	2.52E-46	4.14E-45
CEND1	1.504219496	-0.634740498	1.55E-06	3.99E-06
CD164	1.503165154	6.113530338	4.51E-47	7.61E-46
H2BC5	1.502812847	4.261363717	2.90E-79	9.56E-78
PERP	1.50177968	6.541136488	1.12E-99	5.10E-98
ZHX1	1.499291304	4.592552381	1.83E-42	2.70E-41
SAMD12	1.498093692	3.685253076	1.23E-37	1.60E-36
FMN1	1.495946232	6.707535167	5.62E-71	1.63E-69
PKD2	1.495537826	4.390370353	1.28E-50	2.41E-49
DYNC1I1	1.492476175	0.116504812	1.58E-09	5.32E-09
RNF152	1.491554929	3.406300863	1.86E-51	3.57E-50
ZNF45	1.491022167	4.810223242	2.09E-81	7.15E-80
PRICKLE2	1.490710023	3.3600865	1.19E-35	1.46E-34
ACTG2	1.490565161	0.836053836	1.85E-17	1.08E-16
ATL1	1.490075407	2.84089281	1.73E-46	2.85E-45
TMEM150C	1.489980568	-1.253636336	5.16E-05	1.12E-04
SDC3	1.489166409	8.33858778	5.62E-111	2.99E-109
SLC35F6	1.488976782	7.104093172	4.46E-83	1.57E-81
PCDHB2	1.487990369	0.499236931	5.06E-13	2.22E-12
SRD5A3	1.487467556	2.678422674	6.35E-37	8.11E-36
TTC30A	1.487184661	1.624761739	5.65E-27	5.06E-26
BMP1	1.486499747	6.257797263	1.76E-60	4.17E-59
MAPRE2	1.486263949	4.968156558	5.29E-68	1.46E-66
CCL28	1.485366593	2.763256497	8.72E-39	1.17E-37
KLLN	1.485254283	-1.051958845	6.82E-05	1.45E-04
SERPING1	1.48497384	2.430818775	3.45E-36	4.32E-35
CNTNAP3B	1.484277715	2.169062676	2.95E-31	3.14E-30
KYNU	1.484253141	6.300419891	6.24E-101	2.90E-99

TSNAXIP1	1.484200182	-0.442183361	2.74E-07	7.62E-07
GSTT2B	1.483233429	4.235726652	9.64E-62	2.34E-60
HEATR4	1.482758613	0.11470845	9.01E-12	3.59E-11
PCDHB15	1.48154944	-1.506476732	1.49E-04	3.04E-04
IL13RA2	1.480049627	-0.264513707	4.38E-08	1.31E-07
SEMA3E	1.477161721	4.110598045	8.81E-47	1.47E-45
ASB2	1.476466449	0.088789636	2.67E-10	9.55E-10
CABLES1	1.474163226	6.660086479	5.30E-91	2.10E-89
FLT1	1.473830832	5.375413621	7.25E-61	1.73E-59
GSDMC	1.472957271	-1.899158566	9.47E-04	1.75E-03
LGALS9C	1.472888509	0.682194681	2.89E-17	1.67E-16
PCDHGB1	1.472481635	1.022647312	4.11E-18	2.48E-17
SSBP3	1.471953879	5.54881524	1.55E-80	5.18E-79
RASSF6	1.470067621	-1.899548464	9.33E-04	1.73E-03
STING1	1.469902848	5.385648412	6.73E-67	1.81E-65
BMP8B	1.466544684	5.821569752	1.89E-83	6.72E-82
KLHDC1	1.464021446	0.34351805	9.45E-13	4.05E-12
PGAP3	1.460623697	3.152744915	6.03E-47	1.01E-45
MPPE1	1.460492491	1.741998084	2.82E-27	2.55E-26
PLGLB2	1.460205521	-0.623643298	3.65E-05	8.03E-05
MEGF10	1.459762993	-0.709766758	6.58E-07	1.77E-06
MYO5A	1.459736751	6.113696932	1.10E-47	1.89E-46
FPR1	1.4593802	0.929906669	3.18E-19	2.04E-18
FAM214A	1.459144636	3.667631911	4.03E-48	6.97E-47
DGAT2	1.459049627	3.48971977	1.24E-48	2.20E-47
SHB	1.45452136	6.320474276	1.24E-94	5.19E-93
NEBL	1.45381045	-1.52345168	2.23E-04	4.46E-04
COL5A1	1.453549039	8.839330751	1.88E-63	4.67E-62
EMC10	1.447912334	5.304536176	4.06E-59	9.14E-58
C6orf15	1.446988014	-1.392648056	4.41E-04	8.52E-04
MACC1	1.446015136	-1.59588886	5.23E-04	1.00E-03
ZFP14	1.445652878	1.611801429	8.91E-24	7.07E-23
TTLL6	1.44487706	0.507836116	5.88E-10	2.05E-09
OGFRL1	1.444827227	8.911599136	9.10E-33	1.03E-31
GPR155	1.443587046	3.689498361	5.19E-53	1.03E-51
ABCD3	1.442439827	6.79893438	1.54E-36	1.96E-35
FBN1	1.440704115	7.005252889	1.43E-56	3.08E-55
NOS1AP	1.44048282	2.545430761	4.80E-28	4.49E-27
SOGA3	1.439405932	2.506160623	3.20E-31	3.40E-30
ADAM22	1.438102568	1.409464537	1.73E-21	1.23E-20
CCDC191	1.433448525	-0.349370246	2.41E-06	6.06E-06
MAN2B2	1.429518291	6.727045578	2.21E-78	7.23E-77
ANGPTL4	1.429205438	2.099176314	1.40E-32	1.58E-31
ZNF91	1.427504536	4.789212626	3.35E-42	4.89E-41
ULBP2	1.427462914	4.861762003	9.39E-75	2.87E-73
BBS1	1.424617631	4.029983982	1.73E-61	4.19E-60
C17orf97	1.423480609	0.540749654	1.34E-13	6.14E-13

BAIAP2L2	1.422374861	0.490424871	4.69E-14	2.21E-13
PCDHB13	1.421121902	-1.240278709	5.81E-05	1.25E-04
PLPP3	1.420480221	3.352450379	2.50E-42	3.67E-41
ZNF488	1.418980094	1.673921081	1.59E-25	1.35E-24
BMPR2	1.415897136	6.843282861	1.29E-25	1.10E-24
EPSTI1	1.414003139	1.462383245	1.23E-20	8.46E-20
ATP8A1	1.413612174	-0.993884219	2.22E-05	5.01E-05
PARP8	1.412986421	2.398577123	4.90E-30	4.93E-29
ATP7A	1.409216076	3.863903122	1.76E-14	8.57E-14
BNC1	1.408511364	-0.375411413	1.16E-05	2.70E-05
SPATA9	1.407624212	-1.770955	1.39E-03	2.50E-03
CLSTN3	1.406775977	5.834261502	1.22E-66	3.27E-65
SPTLC3	1.404974278	-1.55780232	9.15E-04	1.69E-03
TIPARP	1.404825261	6.08943094	9.34E-40	1.28E-38
ALS2CL	1.404219991	6.916320624	8.83E-73	2.61E-71
FEZF1	1.403742873	0.969719484	1.57E-14	7.67E-14
GPRC5D	1.402161135	-0.634728188	2.54E-06	6.37E-06
TMEM102	1.401039987	3.257618808	3.72E-36	4.65E-35
DAAM1	1.400798756	5.147180228	3.95E-30	3.99E-29
C1S	1.394151841	1.819843959	2.60E-29	2.55E-28
CAV1	1.392801768	8.428822771	1.32E-79	4.39E-78
CHRM3	1.392610683	-0.113656251	7.04E-09	2.25E-08
CYP27B1	1.392324162	1.211893261	1.27E-18	7.83E-18
BCHE	1.391725716	3.357193706	4.47E-39	6.04E-38
PRKAA2	1.391555078	-0.010286067	3.87E-06	9.52E-06
PTGFR	1.387828519	-1.375389551	2.72E-04	5.37E-04
FKBP1A	1.386757863	9.540119733	1.33E-87	5.04E-86
SLC6A6	1.38615963	7.240757264	2.71E-87	1.02E-85
GRN	1.385928756	8.928709554	5.35E-68	1.47E-66
CCDC187	1.385849544	-1.375671603	2.42E-04	4.80E-04
CNTNAP3C	1.385107805	1.290979873	2.22E-21	1.58E-20
ARHGAP25	1.381799772	0.795335817	3.17E-15	1.62E-14
RHOBTB3	1.380413156	8.77248968	6.37E-35	7.70E-34
TTC7A	1.379996916	7.157818109	2.47E-76	7.86E-75
VWA7	1.378298325	2.121744968	4.62E-26	4.01E-25
SARDH	1.378074642	3.612211259	8.85E-24	7.03E-23
B3GALT4	1.377951477	1.09908305	9.64E-19	5.99E-18
USP30	1.373648046	2.716097539	1.60E-35	1.97E-34
PRPS2	1.372989707	7.965913306	3.17E-87	1.20E-85
MAB21L3	1.37265587	-1.375774785	8.14E-04	1.52E-03
MATN2	1.37221043	8.323498227	1.03E-110	5.46E-109
HLA-DMA	1.371725829	2.282149427	4.35E-20	2.90E-19
ZMIZ2	1.371173243	6.816314446	2.51E-54	5.16E-53
GPRASP1	1.370452928	0.519343553	2.14E-11	8.27E-11
SPDYA	1.369966181	-1.875608606	3.16E-03	5.41E-03
ETV7	1.369646813	0.839431703	2.16E-16	1.18E-15
IKZF2	1.36959629	3.322233649	2.40E-16	1.31E-15

SPRED1	1.368842989	6.379928957	1.96E-22	1.47E-21
KLF10	1.368754236	7.044400919	1.31E-67	3.59E-66
BAMBI	1.368184578	3.886222428	5.43E-51	1.03E-49
C6orf141	1.367789489	2.755601964	9.45E-33	1.07E-31
ERCC6	1.366890457	5.594893812	3.59E-60	8.40E-59
CPEB4	1.366871865	6.391220098	8.89E-19	5.55E-18
PARP9	1.36634323	2.97170024	3.00E-34	3.58E-33
DEPTOR	1.366237444	-0.771937657	5.07E-06	1.23E-05
TRIM62	1.366229498	3.713195077	4.04E-49	7.28E-48
MFHAS1	1.362828171	5.723875836	1.12E-48	2.00E-47
CATSPER3	1.360848485	-0.498000696	3.20E-07	8.86E-07
TAGLN2	1.359890199	9.091916512	2.43E-96	1.05E-94
SLC26A8	1.359592846	-0.208396474	4.11E-07	1.13E-06
PREPL	1.355932014	4.283698293	5.39E-44	8.35E-43
FAR2	1.353537243	-0.140133008	8.31E-09	2.64E-08
EPHX2	1.353458991	1.59940036	7.89E-24	6.28E-23
FHL2	1.352876618	8.878286979	1.17E-116	6.75E-115
WLS	1.352241535	6.398960286	9.52E-85	3.45E-83
RAET1L	1.352085319	1.110767348	1.15E-16	6.42E-16
MITF	1.349612212	3.260153293	7.37E-32	8.06E-31
CXCL16	1.349207044	4.216689837	2.78E-52	5.42E-51
SP140L	1.346662718	3.666718498	5.65E-47	9.49E-46
PCYOX1	1.34554534	7.103406314	1.26E-74	3.82E-73
PRKACB	1.343879636	4.891239669	5.20E-23	3.99E-22
AP1S2	1.341086543	6.667454641	1.11E-47	1.90E-46
GCH1	1.340153345	5.453826452	2.43E-42	3.59E-41
CARMIL1	1.339784287	4.855738332	1.47E-59	3.34E-58
ZNF404	1.339774832	1.509526737	5.84E-15	2.93E-14
SHC3	1.338858327	3.981608018	1.24E-48	2.19E-47
C4orf3	1.337395827	5.736020021	2.62E-68	7.29E-67
CEACAM1	1.337358547	1.4241036	9.23E-19	5.75E-18
C8orf48	1.333670031	-0.614480884	6.46E-07	1.74E-06
MLLT11	1.332288559	3.995197357	5.98E-49	1.07E-47
AGL	1.331445477	4.730134876	4.32E-18	2.60E-17
NEDD9	1.329397131	7.829745294	2.31E-82	7.99E-81
KDF1	1.328757587	-0.876588969	7.36E-06	1.76E-05
PNPLA3	1.326514656	1.153307686	6.33E-19	3.98E-18
CGNL1	1.326107014	-0.315373095	9.27E-08	2.69E-07
TSPAN10	1.325267668	-0.385779185	1.65E-04	3.35E-04
CHST15	1.32246719	6.458277971	2.95E-83	1.04E-81
ALDOC	1.32128023	4.424821238	2.14E-48	3.73E-47
SKAP2	1.320978169	5.002563834	4.25E-32	4.70E-31
CYP4V2	1.318295224	2.862290094	1.95E-28	1.85E-27
ACOT4	1.316669538	2.536617605	8.57E-22	6.22E-21
FAT1	1.316374821	9.815439593	1.44E-49	2.63E-48
DNMT3L	1.315933675	-1.104559018	5.52E-05	1.19E-04
PSTPIP2	1.315275921	2.590355213	1.66E-32	1.86E-31

TGM1	1.31270632	1.584639304	5.33E-20	3.53E-19
ALDH3A2	1.311579263	3.93621065	1.62E-48	2.85E-47
TSPYL4	1.309744196	4.600542365	1.19E-56	2.57E-55
MT1X	1.309530516	6.025398534	1.03E-60	2.46E-59
SCRN3	1.308357209	3.546662393	1.03E-35	1.28E-34
PIK3R3	1.307678155	2.399330252	1.33E-23	1.04E-22
FABP6	1.307320759	0.562219219	3.05E-09	1.01E-08
NIBAN3	1.307104974	-1.833001913	2.16E-03	3.78E-03
CAP2	1.30289332	5.173405888	4.10E-43	6.20E-42
RTBDN	1.302329504	0.094965848	9.72E-09	3.07E-08
CPNE5	1.30203962	2.558832181	2.00E-29	1.97E-28
PLA2G4C	1.300999791	3.704390956	7.73E-39	1.04E-37
PFKFB4	1.300886529	4.872313937	6.03E-64	1.51E-62
PKDREJ	1.300238682	-0.772925041	9.18E-05	1.92E-04
SMIM10L2B	1.299141189	-0.361271569	1.00E-06	2.63E-06
TBKBP1	1.2983098	4.151471465	6.86E-26	5.91E-25
GDPGP1	1.297801658	-0.476104388	2.74E-06	6.84E-06
EFNB2	1.297348114	3.430278216	2.88E-35	3.50E-34
MYO5B	1.296077402	2.43865026	3.90E-21	2.74E-20
TRIM54	1.290514073	-0.307967983	7.59E-07	2.02E-06
RNASEL	1.29015251	3.027077248	1.67E-31	1.80E-30
KIF12	1.289751161	0.429593984	4.06E-12	1.67E-11
ARHGAP28	1.289471631	-0.748856043	1.04E-05	2.43E-05
LMBRD2	1.288726693	4.523235798	9.45E-12	3.75E-11
MCEMP1	1.287789608	-1.328352613	3.85E-04	7.47E-04
ZNF345	1.287394956	0.677958142	7.72E-12	3.09E-11
PFKFB3	1.285365901	7.364369241	3.54E-87	1.33E-85
CDK14	1.284161696	0.58331345	3.83E-06	9.44E-06
ISG15	1.282163666	7.095516291	2.97E-27	2.68E-26
LMBRD1	1.28065767	4.129548405	1.13E-30	1.17E-29
C4orf33	1.280231936	2.604661158	1.87E-33	2.19E-32
FEZ1	1.279125902	0.233261589	5.23E-10	1.83E-09
MFGE8	1.27809804	7.58716579	7.19E-71	2.07E-69
IFFO1	1.275518906	0.64181838	8.72E-13	3.75E-12
ZNF792	1.273889319	3.956784296	8.92E-38	1.17E-36
TOGARAM2	1.273576051	-1.042317853	9.65E-05	2.01E-04
INAFM2	1.272706005	5.445682169	3.32E-67	9.00E-66
SLC22A1	1.27154063	-0.689866879	8.03E-06	1.91E-05
ZNF185	1.271341548	8.508394543	5.01E-93	2.04E-91
TCAF2	1.270917858	4.783067689	1.04E-59	2.37E-58
TM7SF2	1.270716232	3.764670478	1.16E-43	1.79E-42
ABHD8	1.27048282	4.965130303	1.49E-33	1.74E-32
RIMS4	1.270266285	-1.854572097	5.08E-03	8.42E-03
SHROOM3	1.269644207	7.760482455	5.62E-82	1.94E-80
SERPINB4	1.268977487	-1.284427998	3.39E-04	6.62E-04
SRGAP3	1.268266001	-1.708323256	2.93E-03	5.04E-03
TCTN1	1.267196389	3.51213848	7.98E-42	1.15E-40

TIMP2	1.26717122	9.416785904	2.01E-90	7.87E-89
CATSPERE	1.267148228	-0.878137546	4.18E-05	9.14E-05
CELF6	1.266789664	0.416100843	8.62E-09	2.74E-08
TBC1D8B	1.266248283	2.894009639	6.56E-19	4.12E-18
IL7	1.266039303	-0.80226126	1.25E-05	2.90E-05
ACOT6	1.265561186	-1.051901919	3.73E-04	7.25E-04
PER3	1.264177036	3.367137875	1.04E-36	1.32E-35
TLR3	1.262618258	-0.533345901	1.67E-06	4.30E-06
ATP6V1A	1.261523756	6.595673446	5.76E-32	6.33E-31
IFIH1	1.261374233	3.498055448	2.47E-35	3.01E-34
SLC30A4	1.260644771	3.096756656	1.12E-24	9.21E-24
LFNG	1.260148363	2.805932118	5.23E-33	5.97E-32
LRRN2	1.256093583	-1.944427934	5.39E-03	8.90E-03
NSF	1.255775815	6.542269646	1.53E-49	2.79E-48
LRCOL1	1.254965774	-1.944239557	5.00E-03	8.28E-03
SLC12A5	1.254522991	-0.067355376	1.54E-08	4.78E-08
CLN5	1.254431564	2.776016796	5.68E-31	5.97E-30
TTYH1	1.253848119	-1.093366081	4.89E-04	9.39E-04
LBP	1.253109761	-1.052383846	6.70E-04	1.27E-03
FAM151B	1.251921382	-0.671092656	4.73E-05	1.03E-04
PLGLB1	1.251919661	-1.14361629	4.75E-04	9.14E-04
TCTA	1.251361382	4.564107705	2.04E-57	4.45E-56
RAC2	1.250784499	8.395148995	2.51E-86	9.33E-85
ARHGEF26	1.250634084	5.403077629	2.73E-56	5.81E-55
ADAM9	1.250600049	9.449989027	1.84E-36	2.33E-35
GPR1	1.250210356	4.358101343	4.12E-41	5.90E-40
ITGA11	1.24910922	-1.651080862	2.19E-03	3.84E-03
UMAD1	1.248783238	2.800486152	1.64E-30	1.69E-29
MAP3K8	1.24824066	3.029601166	2.38E-28	2.25E-27
DMXL1	1.248101629	4.380022164	3.26E-24	2.63E-23
WNT4	1.247736447	0.212394478	1.61E-09	5.42E-09
GPR39	1.244610999	0.756223908	1.02E-08	3.22E-08
SH3TC2	1.243304558	3.995484279	9.70E-44	1.50E-42
MAP1A	1.242900521	0.346688008	3.52E-07	9.71E-07
VWF	1.240631793	1.411821777	2.67E-17	1.54E-16
UCN2	1.239910066	-1.10413298	2.95E-04	5.81E-04
C6orf132	1.239897968	8.0574553	3.51E-64	8.82E-63
ANO9	1.239809812	1.44738359	5.83E-12	2.36E-11
PSG9	1.238233653	2.242243891	8.75E-27	7.79E-26
PMAIP1	1.237974315	6.851318594	5.47E-36	6.82E-35
IFI6	1.236480592	2.928037195	9.21E-13	3.95E-12
SFRP1	1.235765174	0.273190995	2.71E-09	8.99E-09
KCTD21	1.235627502	4.591929077	1.04E-49	1.91E-48
TENM3	1.234993698	8.130551098	1.62E-75	5.10E-74
ADGRL2	1.233979399	0.653553015	8.71E-10	3.00E-09
DNAH3	1.233786547	-1.255069584	4.21E-04	8.13E-04
CLTRN	1.233155114	0.046614451	4.87E-09	1.57E-08

COL7A1	1.231261854	5.836169115	2.10E-31	2.24E-30
TCEANC	1.230116796	0.047375012	8.36E-08	2.44E-07
ITM2B	1.229857977	7.732983795	3.46E-63	8.55E-62
LPXN	1.22951489	6.536163669	4.01E-75	1.24E-73
ICAM2	1.228394696	2.966537609	9.32E-22	6.73E-21
DNAJC28	1.227867788	-0.524932263	4.53E-06	1.11E-05
PIGZ	1.227447313	2.152500022	1.30E-25	1.11E-24
TTC30B	1.227260565	1.003368396	2.68E-13	1.20E-12
DHRS3	1.227015789	0.990156834	1.58E-11	6.16E-11
ADAM19	1.226342568	8.560712897	1.41E-85	5.17E-84
SCN4B	1.226155128	1.17984586	6.00E-15	3.01E-14
TMEM80	1.225882926	2.665861792	3.97E-21	2.79E-20
SPRY1	1.224545278	3.418170293	3.81E-33	4.38E-32
CYB561D1	1.224504515	4.56219777	7.27E-47	1.21E-45
STOX1	1.224243086	0.15808842	8.56E-09	2.72E-08
SLC25A45	1.222852119	2.69526221	2.79E-30	2.84E-29
EFCAB7	1.222714406	1.548537358	3.15E-11	1.20E-10
ABCC9	1.222682919	-1.118088179	1.89E-04	3.80E-04
OLR1	1.222519446	6.029808756	1.86E-53	3.73E-52
ARSL	1.222402418	0.94632129	2.54E-12	1.06E-11
ZNF718	1.219217092	3.567731754	2.39E-29	2.35E-28
PLK2	1.218415041	8.413970888	1.56E-77	5.08E-76
PPP1R1C	1.216626202	2.911046303	2.02E-35	2.48E-34
DCBLD2	1.215618111	7.948878219	8.51E-21	5.88E-20
PCTP	1.215023424	4.613737859	1.24E-53	2.50E-52
PDE1C	1.213687967	4.822087672	7.16E-34	8.46E-33
TBC1D19	1.213304749	1.878953234	4.87E-17	2.77E-16
IGFN1	1.212978945	-1.96762672	1.17E-02	1.84E-02
TRPM2	1.212972483	1.888142258	9.63E-18	5.70E-17
MST1R	1.211701076	4.885915068	1.56E-48	2.74E-47
CPQ	1.21129103	2.478545449	1.96E-23	1.53E-22
SPECC1	1.211151209	6.85014031	5.38E-70	1.55E-68
ZCWPW2	1.210374684	-1.488674422	1.56E-03	2.80E-03
CASTOR2	1.210086131	5.887156189	4.10E-59	9.21E-58
PSORS1C1	1.209175713	3.708877211	2.03E-36	2.56E-35
TIMP3	1.208393586	5.989964884	1.30E-65	3.39E-64
CCDC74B	1.207281751	-0.098670318	5.19E-08	1.54E-07
PAFAH2	1.206648799	3.208778654	1.79E-25	1.52E-24
BTBD9	1.206180265	5.378824611	8.98E-49	1.60E-47
MYOZ2	1.206110092	-0.263533223	9.99E-07	2.62E-06
ARRDC3	1.205847055	4.636887679	8.91E-25	7.36E-24
B3GNT3	1.204294799	4.328903667	1.13E-45	1.81E-44
C22orf24	1.20309626	-0.770348244	2.03E-05	4.60E-05
ZFHX2	1.202985699	2.134592906	4.38E-22	3.24E-21
CGN	1.201170948	2.396172512	2.46E-27	2.23E-26
CDNF	1.199305508	-1.228087939	7.69E-04	1.44E-03
WDR93	1.198942197	-1.228264469	7.53E-04	1.41E-03

NUDT18	1.198842247	1.720864233	8.20E-15	4.08E-14
SIN3B	1.194423114	7.479762369	5.65E-51	1.07E-49
ATP9A	1.193761761	7.019302274	1.03E-55	2.18E-54
GALNT5	1.193142432	5.455297595	1.23E-42	1.83E-41
SLC25A20	1.191284897	2.533478013	3.96E-30	4.00E-29
SPRY4	1.190999783	7.061823098	2.20E-75	6.86E-74
TRPS1	1.190857035	3.40920361	2.87E-26	2.51E-25
SEMA3C	1.190491611	4.568534257	6.46E-13	2.81E-12
VIPR1	1.18999059	-0.249097153	6.95E-07	1.86E-06
EHD1	1.189385608	8.717319505	1.02E-31	1.10E-30
AL353753.1	1.189174171	0.615168123	1.12E-06	2.93E-06
BAZ2B	1.188774252	4.228113172	1.14E-18	7.01E-18
ZNF397	1.187516478	2.33279886	4.45E-22	3.28E-21
HACD2	1.18677428	3.944555383	3.94E-21	2.77E-20
PIP4K2C	1.18642551	6.338410779	1.36E-67	3.71E-66
LPIN1	1.185818651	6.39627173	1.49E-55	3.14E-54
MMAA	1.183680442	1.05481175	4.18E-14	1.98E-13
TMEM178B	1.181297712	-0.68134912	1.97E-05	4.49E-05
TMEM86A	1.180927583	2.006941354	3.39E-15	1.72E-14
ICA1	1.180476996	1.630162046	5.95E-20	3.93E-19
FAM161B	1.180100292	2.714871631	1.09E-23	8.62E-23
NIPSNAP3B	1.179344949	-0.960352162	7.82E-03	1.26E-02
ZNF432	1.17914238	4.438983288	2.75E-42	4.03E-41
LSP1	1.178903	-1.506053653	2.01E-03	3.55E-03
LRIG1	1.178523625	5.416945364	1.24E-50	2.33E-49
UNC13A	1.176780782	3.147953358	9.35E-29	8.95E-28
C19orf18	1.176087607	-0.055773632	9.63E-08	2.79E-07
ZNF260	1.175206639	3.597507051	6.23E-11	2.32E-10
NRP1	1.174895437	7.750726356	1.27E-69	3.62E-68
FAM83E	1.174849488	1.003366948	8.86E-10	3.05E-09
ZDHHC9	1.174763519	7.331514188	7.07E-71	2.05E-69
MAPK8IP2	1.174539197	-0.970863908	7.36E-04	1.38E-03
CERCAM	1.173688836	7.534921344	4.87E-46	7.93E-45
ADD3	1.173354556	5.282430223	1.19E-21	8.53E-21
AMOTL1	1.172841543	7.793767573	1.60E-69	4.54E-68
MEF2A	1.172725441	5.286531574	1.55E-44	2.44E-43
NEURL3	1.172495526	-0.980649991	1.70E-04	3.45E-04
PLPP6	1.172390016	1.086264953	4.27E-15	2.16E-14
PLAC8	1.172257298	6.819321644	4.37E-67	1.18E-65
RPS6KA2	1.171108351	5.655302314	7.26E-54	1.47E-52
DHDH	1.169976301	-0.980861013	1.33E-04	2.73E-04
LIPA	1.167811079	6.997948935	1.06E-60	2.51E-59
MUC1	1.16600843	1.860716312	2.33E-13	1.05E-12
BTBD11	1.165966383	-1.155909679	5.62E-04	1.07E-03
STYK1	1.165321389	4.441410762	3.04E-31	3.24E-30
NIPAL2	1.165122607	4.003419011	4.46E-37	5.73E-36
SH3BP5	1.164994132	3.441948413	3.45E-35	4.19E-34

MYPN	1.164817574	4.115995996	1.69E-37	2.18E-36
REN	1.164662125	0.327069766	4.99E-09	1.61E-08
QSOX1	1.163983147	10.17194067	3.37E-64	8.50E-63
SORCS2	1.16363413	7.004456324	2.12E-45	3.39E-44
SLC9A9	1.163332597	-0.760115792	6.37E-05	1.36E-04
EXOC3L1	1.162275931	-1.406681676	2.13E-03	3.74E-03
AMOT	1.16197313	-1.577080004	3.80E-03	6.43E-03
SORL1	1.157753453	-1.407034954	1.86E-03	3.29E-03
RIOK3	1.157738719	7.23399542	1.40E-40	1.97E-39
KDM4B	1.15756093	6.588516255	3.13E-44	4.87E-43
GMNC	1.156671809	-1.254594802	1.43E-03	2.57E-03
NRG1	1.156513519	6.543955528	1.36E-53	2.74E-52
CDK18	1.155150131	4.373779191	4.54E-40	6.30E-39
ENPP4	1.154071368	3.627228819	3.64E-18	2.20E-17
SHISA2	1.153895331	5.526550017	2.27E-41	3.28E-40
MAPKBP1	1.151676396	6.943047808	1.84E-60	4.33E-59
HPS3	1.147734588	5.765767867	4.51E-37	5.79E-36
MAML2	1.14734099	4.977680995	2.87E-23	2.22E-22
FRMD5	1.147210635	5.045837244	2.10E-49	3.83E-48
ATXN1	1.146974059	6.854251594	1.79E-65	4.64E-64
SLC25A41	1.14621499	-1.52473333	4.16E-03	6.99E-03
ZMIZ1	1.146043052	6.725991173	3.54E-54	7.23E-53
KCNAB1	1.145973554	0.691952562	2.74E-07	7.61E-07
HIPK2	1.14550798	8.401126464	1.67E-75	5.22E-74
PLXNA3	1.144829501	7.038620158	1.59E-37	2.06E-36
LPAR1	1.144668219	3.828005601	8.56E-29	8.21E-28
RHD	1.14329608	-1.31379191	1.24E-03	2.25E-03
USH2A	1.14236868	-1.423147495	2.69E-03	4.66E-03
SMPDL3B	1.142350245	1.796988765	6.69E-17	3.78E-16
CTSS	1.141809364	4.970509011	8.15E-48	1.40E-46
SELENOT	1.140021921	6.536650894	3.64E-51	6.94E-50
ZNF493	1.139894294	1.704030067	2.23E-17	1.29E-16
ZNF879	1.139843815	0.576127712	1.25E-09	4.26E-09
CACNA2D1	1.139688976	3.881851067	1.19E-14	5.85E-14
FLG	1.138629114	7.083055193	3.62E-45	5.76E-44
FOXC1	1.137951649	5.067867571	4.15E-42	6.04E-41
P4HA1	1.136571449	7.086810264	4.99E-41	7.14E-40
FAM227A	1.136489281	-0.434578686	1.32E-04	2.70E-04
TMEM175	1.136335867	3.303441467	2.50E-25	2.11E-24
NCOA1	1.136093564	5.330548174	1.40E-40	1.97E-39
CCDC115	1.136041024	4.514701303	5.92E-40	8.13E-39
TRPM6	1.135839399	-0.88643082	1.79E-04	3.61E-04
MAML3	1.132029577	5.04670228	1.15E-46	1.91E-45
AOX1	1.131742754	7.307030625	9.74E-38	1.27E-36
ADAMTS12	1.131727637	8.028124754	6.84E-72	2.00E-70
TMLHE	1.13162364	1.247624984	6.61E-15	3.31E-14
AK8	1.131619239	-0.019599073	1.15E-06	3.00E-06

LPIN3	1.131396952	4.572221962	2.38E-28	2.25E-27
DCST2	1.129848263	-0.393027159	9.47E-06	2.23E-05
GALNT1	1.129822287	5.871175822	1.90E-27	1.73E-26
DMD	1.129007283	3.114481918	2.13E-20	1.44E-19
ZNF738	1.128904332	2.939815819	5.97E-25	4.98E-24
TMEM64	1.126855495	5.717304501	5.74E-17	3.25E-16
KITLG	1.126508684	2.841037436	3.89E-15	1.97E-14
LAMC1	1.125145961	8.707564644	3.05E-68	8.45E-67
THSD4	1.124604102	9.197759175	6.25E-79	2.05E-77
ZNF772	1.124101826	4.172268025	4.38E-13	1.93E-12
GCOM1	1.122793398	-0.183751871	3.60E-05	7.92E-05
VANGL2	1.122466081	-1.727957191	8.97E-03	1.43E-02
IL1RAP	1.121447044	5.344741231	1.75E-25	1.49E-24
TIAM1	1.121127191	5.2493869	9.85E-47	1.63E-45
FSIP2	1.120118587	-0.625170485	4.20E-04	8.13E-04
AFDN	1.119471853	7.525197972	5.30E-46	8.61E-45
B4GALT1	1.119402646	8.351955151	2.05E-66	5.46E-65
ADCY5	1.118834448	-1.424287793	6.26E-03	1.02E-02
MAGEC3	1.118228264	0.798799598	1.90E-09	6.37E-09
ADAM10	1.115027894	8.841023525	9.46E-34	1.11E-32
CLTB	1.11479721	7.811376595	9.22E-34	1.09E-32
RUNX1	1.114790153	7.008182968	2.67E-59	6.04E-58
NCKAP1L	1.114223882	-1.48784675	4.83E-03	8.03E-03
KLHL41	1.113517719	-1.14543689	9.42E-04	1.74E-03
SMPD1	1.112295799	6.352582238	8.14E-45	1.29E-43
PPP1R12B	1.112106376	4.1553131	4.95E-43	7.45E-42
MKRN3	1.112086909	1.61614034	2.95E-18	1.78E-17
SMIM1	1.11182976	-0.376951996	1.28E-05	2.98E-05
MMP13	1.108924552	-1.054874173	5.23E-03	8.65E-03
MATK	1.108624716	1.221105725	1.17E-09	3.99E-09
CABLES2	1.108338509	5.733366224	9.42E-50	1.74E-48
C5AR1	1.108148281	-1.103896068	8.27E-04	1.54E-03
PXDN	1.107476533	9.399726159	8.35E-69	2.34E-67
AGBL2	1.107032061	-0.944543212	2.38E-03	4.15E-03
OTUD7B	1.105888944	6.599130232	1.17E-64	3.00E-63
CARF	1.105363945	0.537233972	4.78E-05	1.04E-04
PAPSS1	1.105072581	5.751278893	1.79E-39	2.44E-38
GLRX	1.104973673	5.792798745	1.23E-44	1.93E-43
FOSL2	1.104028513	9.743106302	4.62E-73	1.37E-71
FAM227B	1.103737181	-1.106344295	1.10E-03	2.02E-03
AVIL	1.103443186	0.857720181	1.53E-08	4.74E-08
PLXNB3	1.102888129	4.894254224	4.00E-22	2.96E-21
AC098582.1	1.102762476	5.317624737	7.48E-19	4.68E-18
HERC3	1.102762476	5.317624737	7.48E-19	4.68E-18
FRY	1.102049928	1.084959897	1.55E-12	6.55E-12
IFIT3	1.101906069	5.859552458	6.52E-50	1.21E-48
TMEM59	1.101768357	7.06592771	1.01E-59	2.32E-58

CTRL	1.098356245	0.844673113	1.02E-07	2.95E-07
BNIP3L	1.098180693	6.784968558	6.49E-47	1.09E-45
KLF4	1.097799267	5.14967708	6.68E-44	1.03E-42
RTL8C	1.096096552	7.060614191	4.17E-40	5.79E-39
FAM136A	1.095751538	6.316267012	1.79E-56	3.83E-55
IRF6	1.095004391	1.287299238	3.85E-14	1.83E-13
TLE6	1.093916323	-0.52455221	2.54E-05	5.70E-05
DPF3	1.092854312	2.424427248	2.48E-23	1.93E-22
TM2D2	1.092335873	5.792683767	2.81E-46	4.60E-45
TNS3	1.091958097	7.783342427	6.43E-61	1.54E-59
DTWD2	1.091460569	1.278361261	1.14E-06	2.99E-06
TUBB1	1.09052606	0.342812406	2.84E-08	8.61E-08
RPGRIP1	1.08974979	0.307831752	2.59E-06	6.50E-06
AKAP6	1.089413487	-0.368330586	2.86E-05	6.37E-05
NLRC3	1.088632281	-0.448754123	2.22E-04	4.44E-04
LYPD6	1.087847727	4.813838614	3.01E-38	4.00E-37
FAM210B	1.087522187	4.880448592	4.06E-46	6.63E-45
ZNF827	1.085996481	4.660819258	2.65E-40	3.70E-39
KCNH1	1.084439922	0.606787934	1.13E-09	3.85E-09
TGOLN2	1.083368419	8.205251014	4.21E-67	1.14E-65
SCN2B	1.082930688	-1.507214874	5.66E-03	9.33E-03
MYZAP	1.082865062	0.525294142	1.16E-09	3.94E-09
FNDC4	1.082647377	2.560930689	1.23E-21	8.83E-21
ATP13A2	1.08164241	8.534013359	2.50E-34	2.99E-33
PLEKHG6	1.08151853	0.500661415	1.77E-06	4.54E-06
TLR4	1.080839907	5.493152377	1.39E-24	1.13E-23
DKK3	1.080813322	2.79353905	3.23E-22	2.39E-21
NBDY	1.080708544	4.518134824	5.15E-43	7.75E-42
MAT1A	1.07983595	-0.400679328	2.93E-05	6.51E-05
FOXO4	1.079281929	3.33240719	7.84E-22	5.69E-21
RNF182	1.078450476	4.720743345	3.36E-26	2.93E-25
FGD5	1.078130621	-1.688878016	9.73E-03	1.54E-02
RIMS3	1.076887037	3.752173854	2.84E-31	3.03E-30
GANC	1.076883644	4.329488819	6.75E-39	9.10E-38
TBC1D32	1.074377694	0.405693435	3.37E-08	1.01E-07
STX12	1.073944818	5.55072871	2.05E-43	3.11E-42
NCEH1	1.073787402	7.220650466	3.25E-25	2.73E-24
PRR19	1.073654568	1.519040116	3.41E-15	1.73E-14
MVP	1.072635924	7.816768236	1.61E-36	2.04E-35
NLGN3	1.072070361	2.048030128	5.52E-14	2.60E-13
AP002336.5	1.071595464	7.233821044	1.01E-42	1.51E-41
PPFIA1	1.071595464	7.233821044	1.01E-42	1.51E-41
SLC7A5	1.071025924	11.08026116	1.79E-46	2.94E-45
PAM	1.070817304	8.112217967	2.93E-53	5.81E-52
ALAD	1.070515895	4.348043751	6.70E-42	9.71E-41
TMEM106B	1.070025727	4.490204692	9.72E-19	6.04E-18
MXD4	1.069880182	6.395658009	2.76E-49	5.00E-48

GRK4	1.069586404	1.84979449	1.99E-16	1.09E-15
PTPN6	1.068451647	3.649394868	1.49E-29	1.48E-28
CCDC9B	1.066416754	6.736590488	1.18E-45	1.90E-44
KRCC1	1.066355561	3.542544331	9.74E-28	8.98E-27
GP1BA	1.064282737	0.999191856	1.47E-08	4.56E-08
HPS1	1.063471316	5.711691965	7.38E-48	1.27E-46
IL32	1.062378461	7.524597525	2.55E-47	4.33E-46
CNPY4	1.062086166	2.460908032	1.38E-18	8.49E-18
ZNF431	1.061351182	4.161783801	2.34E-28	2.22E-27
STOML1	1.061246279	4.62284591	3.05E-39	4.13E-38
CRYGS	1.060723264	-0.969397747	8.03E-04	1.50E-03
EHD2	1.060430421	7.880088629	3.47E-41	4.99E-40
OTX1	1.059788079	1.445846174	2.66E-15	1.36E-14
SLC16A8	1.059039196	-0.08552517	3.83E-05	8.40E-05
CHST2	1.058797327	3.611993052	1.15E-24	9.42E-24
MAGEE1	1.058045893	2.732039902	1.73E-20	1.18E-19
REEP6	1.057644455	3.911990587	5.25E-17	2.98E-16
FGD4	1.056656977	2.485920045	1.49E-15	7.81E-15
SLC16A4	1.055793956	-0.112985611	1.46E-06	3.77E-06
ELAVL3	1.055260827	-1.832962174	1.80E-02	2.74E-02
RBPMS	1.055002227	4.806394661	4.39E-39	5.94E-38
STC2	1.053714704	1.126665209	2.06E-11	7.96E-11
GINS4	1.053247985	6.548737986	3.64E-52	7.09E-51
TMOD2	1.052713528	3.99901296	4.38E-13	1.93E-12
NRIP1	1.052656718	6.039242039	6.59E-25	5.48E-24
MIDN	1.052497504	5.962191554	3.43E-25	2.88E-24
PSG6	1.052202512	1.871940527	1.35E-17	7.90E-17
ADPRH	1.051593221	0.889014215	1.08E-07	3.10E-07
CLIC3	1.051113881	4.179018415	5.82E-20	3.85E-19
LRRC56	1.051109897	0.121461059	4.13E-04	7.99E-04
KSR1	1.050692248	4.661751574	3.02E-38	4.01E-37
ZNF681	1.050597409	2.571863506	3.50E-22	2.59E-21
ABHD12	1.050375666	6.500597603	4.96E-42	7.20E-41
MYO1D	1.049847064	2.445125836	3.37E-16	1.82E-15
C4orf54	1.04967932	-1.421979735	7.01E-03	1.14E-02
ZNF112	1.04928539	1.108031759	4.06E-10	1.43E-09
RCOR3	1.048531797	4.513374474	4.21E-33	4.83E-32
PPP1R3G	1.047402844	-0.83432149	3.61E-04	7.03E-04
PVRIG	1.046514265	2.646540729	5.25E-18	3.14E-17
BMP8A	1.046479522	3.014518618	6.22E-23	4.76E-22
TMEM14A	1.046271382	3.879880102	1.94E-30	1.98E-29
C1QTNF2	1.045344277	0.416159676	8.87E-09	2.81E-08
ZNF425	1.044956029	1.476912491	7.35E-15	3.67E-14
MYO6	1.044252755	7.084091592	3.84E-23	2.96E-22
CDKN1A	1.044130612	9.174286752	1.59E-54	3.29E-53
DGKZ	1.043305557	7.233779155	5.41E-28	5.04E-27
ME3	1.043253401	4.978222075	2.99E-36	3.75E-35

LY75	1.04316655	-0.330363895	1.20E-03	2.18E-03
ARL3	1.042590332	5.454369906	2.45E-42	3.61E-41
OS9	1.04052035	6.998093626	6.65E-47	1.11E-45
FBXO16	1.040315107	0.143756098	1.32E-06	3.44E-06
MSLN	1.04010245	1.466755544	1.02E-09	3.49E-09
MEIS2	1.038501951	3.665757419	6.68E-31	6.99E-30
H2BC4	1.03830676	2.110460643	5.60E-19	3.53E-18
DTNB	1.036501861	3.945192721	4.60E-31	4.86E-30
OPHN1	1.035758781	7.613482097	4.75E-31	5.01E-30
STAG3	1.035113054	1.304207872	4.43E-13	1.95E-12
LAMP2	1.034380906	8.044110787	4.28E-43	6.46E-42
GAA	1.033846728	7.231856353	7.91E-37	1.01E-35
CILP2	1.033773793	3.222381386	4.33E-14	2.05E-13
MYLK4	1.033419564	-0.081275272	6.96E-05	1.48E-04
NPL	1.032142686	-0.652037275	1.71E-04	3.46E-04
BAIAP2L1	1.031886384	5.595020148	2.04E-40	2.86E-39
UBE2H	1.031561512	7.855506987	4.20E-65	1.08E-63
CAV2	1.030482993	6.244706935	1.40E-33	1.64E-32
NICN1	1.030389087	2.939888918	7.20E-24	5.74E-23
LMF1	1.030345	3.027942742	1.93E-19	1.25E-18
ZC2HC1A	1.029678621	2.539757144	2.22E-13	9.98E-13
NECTIN3	1.029407757	5.330648854	1.23E-14	6.08E-14
NCDN	1.02749998	5.582513157	1.44E-29	1.42E-28
PLEKHA5	1.026886211	4.821970979	6.50E-26	5.60E-25
ITGAV	1.026833808	7.359057683	2.89E-08	8.76E-08
RECK	1.026663039	4.112677377	1.72E-27	1.58E-26
ZNF429	1.02647204	2.413444329	3.14E-19	2.01E-18
RAG1	1.025725145	0.202299802	2.32E-06	5.86E-06
COL4A3	1.024965775	-0.958886059	4.91E-03	8.16E-03
ALDH3B1	1.024683716	4.42838804	2.87E-21	2.03E-20
KALRN	1.02425855	1.731495064	5.99E-12	2.43E-11
HERC6	1.024239354	3.848488573	1.04E-30	1.08E-29
GTF2IRD2B	1.023808482	3.28507731	1.31E-24	1.07E-23
TMEM182	1.023094667	1.672641454	3.61E-15	1.83E-14
SH3BGR	1.022312849	-0.016949366	1.28E-05	2.98E-05
POLN	1.020065792	-0.992112762	3.05E-03	5.23E-03
RDX	1.020044187	8.252249432	3.06E-42	4.48E-41
KDM4C	1.019143639	2.72128456	9.79E-13	4.19E-12
ZC4H2	1.018977376	2.285377502	3.78E-20	2.53E-19
WNT2B	1.018967913	0.931576782	1.50E-07	4.28E-07
ADAMTS16	1.018435515	5.48394203	1.81E-36	2.29E-35
WDR66	1.017163956	3.851858367	3.01E-33	3.49E-32
KRT15	1.017060764	4.351795886	4.47E-32	4.94E-31
RASSF8	1.016943835	4.696417021	1.64E-11	6.38E-11
SRGN	1.016849364	8.61118145	2.02E-40	2.83E-39
TSPAN6	1.015262119	4.801413124	1.65E-28	1.57E-27
TANGO6	1.015166598	6.044962328	2.61E-43	3.96E-42

TP53INP1	1.015001154	3.745037856	1.52E-14	7.43E-14
WBP1L	1.014623977	6.175157375	1.71E-48	2.99E-47
MXRA8	1.014364607	1.119558015	9.75E-10	3.34E-09
PLEKHH2	1.013614261	2.340144186	7.40E-13	3.20E-12
TMEM178A	1.013492558	-1.789668818	2.01E-02	3.03E-02
PI16	1.012858697	-0.604677803	1.57E-04	3.19E-04
TENM4	1.012639101	0.366461282	1.03E-07	2.98E-07
RINL	1.012232855	0.258992887	1.53E-06	3.94E-06
GNG4	1.011592354	-1.080178836	1.78E-03	3.15E-03
PGK1	1.011422255	10.32329657	1.04E-62	2.57E-61
ARHGAP45	1.010451722	7.088254437	9.99E-18	5.91E-17
ADM	1.010028419	6.412930916	5.23E-46	8.51E-45
TRIM17	1.009992929	-0.634114616	5.98E-04	1.13E-03
IRF9	1.008270519	5.016446024	1.58E-40	2.23E-39
ADAMTSL4	1.006566813	5.363647828	7.10E-25	5.89E-24
UBL3	1.005693769	6.303839676	1.78E-18	1.09E-17
LCP2	1.004807207	-1.00471657	1.35E-03	2.45E-03
NOXA1	1.004271214	2.47041793	1.29E-13	5.90E-13
THSD7B	1.003840774	2.007023435	8.07E-16	4.29E-15
RHOD	1.00347531	5.36101013	3.39E-23	2.62E-22
PADI3	1.002410812	-0.670455888	2.54E-04	5.03E-04
LRRC3	1.002052781	3.024577927	2.85E-22	2.11E-21
TEX2	1.000729648	6.849692041	3.55E-48	6.15E-47
PRR36	-1.000127237	1.361914869	2.84E-11	1.09E-10
SAMD1	-1.000932894	4.088145472	3.60E-26	3.14E-25
RAD54B	-1.001932203	3.358484073	1.52E-19	9.89E-19
GOLGA8F	-1.003135858	-1.707072853	1.26E-02	1.98E-02
MIIP	-1.003423384	4.661049199	1.64E-28	1.56E-27
TGFB1I1	-1.00534925	5.145827364	1.57E-26	1.38E-25
GSDMD	-1.005654755	5.600194613	4.22E-20	2.81E-19
MRPS18B	-1.005655534	6.660594843	1.19E-50	2.24E-49
THEMIS2	-1.00669471	3.51815399	3.50E-25	2.93E-24
KIF23	-1.008991228	6.622396589	5.47E-25	4.56E-24
TIGD3	-1.009347514	0.501754924	1.18E-05	2.76E-05
CSRNP1	-1.009773694	4.566306389	3.82E-33	4.39E-32
ARHGAP9	-1.00993535	-1.223845336	3.96E-03	6.68E-03
NAGS	-1.010262369	4.580057857	2.98E-29	2.91E-28
TNFSF8	-1.010707538	-1.592486797	9.79E-03	1.55E-02
ABLIM1	-1.010910041	5.31883847	1.29E-29	1.28E-28
CUTC	-1.011496163	3.231874234	4.53E-25	3.79E-24
TPRN	-1.011832208	4.41531898	1.50E-19	9.72E-19
PER1	-1.01207916	3.624113288	8.72E-22	6.31E-21
ADM5	-1.012615242	2.14636507	1.43E-14	7.01E-14
TMEM268	-1.013800376	3.948918488	2.83E-28	2.66E-27
HSPA9	-1.014358006	8.30746954	5.25E-46	8.53E-45
HSPBAP1	-1.014413087	2.467516115	1.20E-20	8.20E-20
ZNF695	-1.014857323	0.619444358	1.23E-08	3.84E-08

RFC4	-1.015115695	4.948568662	1.01E-33	1.18E-32
FBXL13	-1.015561664	1.230269075	2.03E-10	7.33E-10
FAM221A	-1.017002639	1.500010801	4.63E-14	2.18E-13
RPS6KL1	-1.017228306	3.738337158	1.23E-26	1.09E-25
METAP1D	-1.01763849	3.155860006	1.01E-28	9.66E-28
KCNK7	-1.017669979	-0.417846921	3.92E-04	7.62E-04
DLK2	-1.018264321	4.113078997	5.48E-27	4.91E-26
DUS4L	-1.018992836	2.229981801	1.08E-16	6.00E-16
CAMK1D	-1.02183878	0.868092463	2.20E-05	4.98E-05
SNRPF	-1.021860551	5.211877672	1.12E-32	1.27E-31
EME1	-1.022495092	3.09548415	7.95E-25	6.58E-24
HCAR1	-1.023350943	-1.253032301	4.95E-03	8.21E-03
ABCB6	-1.023649667	3.206040803	5.34E-24	4.29E-23
NAALAD2	-1.024154749	-1.34149396	4.44E-03	7.43E-03
SLC4A11	-1.025144995	2.331678347	4.21E-20	2.81E-19
IRAK2	-1.025164474	4.123432822	3.82E-29	3.72E-28
EXOSC9	-1.025906132	4.447714433	6.68E-29	6.43E-28
CCDC59	-1.026181923	3.909588282	4.92E-32	5.42E-31
EDIL3	-1.026809805	5.750957904	1.08E-10	3.97E-10
H4C1	-1.027006268	-0.85176791	3.56E-04	6.95E-04
CDHR5	-1.027800625	1.331641878	2.81E-09	9.30E-09
MRPS2	-1.028337877	5.924856997	7.02E-31	7.34E-30
UBE2S	-1.029423231	7.962831691	3.09E-30	3.14E-29
NOD2	-1.030021062	0.254782122	1.06E-07	3.05E-07
KIF14	-1.030206668	5.115636669	1.17E-14	5.76E-14
HAUS7	-1.030317993	5.033716039	2.58E-29	2.53E-28
MTHFD2	-1.031618535	5.998864521	5.71E-26	4.93E-25
ABCC4	-1.031707269	3.252821109	2.65E-10	9.48E-10
ZFYVE28	-1.031804516	3.577890681	1.38E-29	1.37E-28
C1orf54	-1.031926472	-1.074698441	1.06E-02	1.67E-02
NSMCE4A	-1.033803081	3.597489072	1.17E-30	1.21E-29
CLEC11A	-1.034113929	1.917081223	7.00E-11	2.60E-10
CCDC86	-1.035194593	6.166144624	3.76E-33	4.33E-32
SLC25A22	-1.035278908	4.84004125	5.55E-29	5.36E-28
LSM2	-1.035311442	5.470016642	1.88E-29	1.85E-28
VCAN	-1.035808038	1.737639153	4.51E-12	1.85E-11
FAM241A	-1.035927576	2.515172985	6.00E-19	3.78E-18
GOLGA8G	-1.037757555	-1.68712762	9.40E-03	1.49E-02
HACD1	-1.038111668	3.319870083	1.73E-22	1.30E-21
CRB2	-1.038144418	0.293194009	5.11E-05	1.11E-04
MTFR2	-1.038578953	3.006323841	1.45E-24	1.19E-23
TAF5	-1.038859625	3.506696768	8.95E-25	7.39E-24
NTHL1	-1.041237168	3.14901615	1.20E-16	6.65E-16
BAIAP2	-1.041664998	5.08595729	1.87E-20	1.27E-19
IVNS1ABP	-1.042824724	5.427761809	1.76E-27	1.61E-26
MSRB3	-1.043182524	4.871449825	7.39E-29	7.10E-28
TWNK	-1.043604545	5.000379673	1.55E-36	1.97E-35

ARHGEF40	-1.044294077	4.175271055	5.97E-32	6.55E-31
CACYBP	-1.044309613	6.399823947	1.77E-47	3.02E-46
AOC3	-1.044879452	1.450373368	5.45E-14	2.56E-13
RORA	-1.045513052	-0.504867124	2.24E-04	4.47E-04
DBF4	-1.045776711	4.187136877	2.03E-23	1.58E-22
CCDC58	-1.045863124	2.545584608	5.06E-22	3.72E-21
IFITM1	-1.046342308	3.337165421	1.89E-23	1.48E-22
ZFP36L1	-1.046687442	7.673075633	3.29E-48	5.71E-47
SPC24	-1.046775206	4.058613902	3.19E-23	2.46E-22
PRX	-1.047932514	2.500219	8.46E-18	5.02E-17
PAK1IP1	-1.048864952	4.756024691	1.66E-34	1.99E-33
H2AC17	-1.049473821	0.543288585	1.03E-05	2.41E-05
PTGR1	-1.050489409	5.375190268	1.34E-40	1.89E-39
MTMR4	-1.050817913	5.727823964	2.25E-49	4.09E-48
CCNB2	-1.050849319	6.040145151	1.11E-52	2.19E-51
EIF4E3	-1.051168296	0.570382129	1.31E-05	3.05E-05
C7orf57	-1.051430368	0.575881364	2.56E-08	7.82E-08
ARHGEF2	-1.053385415	7.56632695	1.71E-54	3.51E-53
TMEM61	-1.053744311	-0.395083629	2.84E-04	5.60E-04
HBEGF	-1.054882954	5.024222282	5.00E-38	6.59E-37
APOBR	-1.05502443	-1.195526354	1.73E-03	3.09E-03
KIF22	-1.055654267	5.97806204	1.21E-50	2.28E-49
CAMKK1	-1.0563956	2.833177411	5.67E-24	4.54E-23
SPNS2	-1.058948399	1.066948542	1.27E-12	5.36E-12
GMPR	-1.06290712	3.894156244	2.30E-34	2.75E-33
IL7R	-1.064257447	7.420690685	1.20E-23	9.44E-23
CDKN3	-1.064875142	5.954619492	5.96E-53	1.18E-51
TMA16	-1.065946476	3.856206606	9.01E-31	9.40E-30
RELL2	-1.066585094	2.444077982	3.00E-16	1.63E-15
PRRG4	-1.067914456	2.520928989	2.92E-18	1.77E-17
TMC8	-1.068273927	1.516566777	3.44E-10	1.22E-09
SERPINE1	-1.069441637	10.39484556	9.96E-77	3.18E-75
SLFNL1	-1.069466804	2.099459283	1.46E-12	6.18E-12
UBALD2	-1.069669771	4.795818371	1.16E-23	9.13E-23
WDR74	-1.070670966	5.442963526	1.67E-29	1.65E-28
H1-10	-1.071463227	5.51714921	1.57E-21	1.13E-20
SLIT2	-1.072127073	5.278918118	1.04E-35	1.29E-34
NUP37	-1.072849785	4.974875195	8.60E-47	1.43E-45
RGS7	-1.073199121	3.950075446	9.64E-36	1.20E-34
RXRA	-1.0732847	5.805368196	2.41E-44	3.77E-43
TGIF1	-1.0736004	5.525503827	3.55E-42	5.17E-41
EPPK1	-1.075012368	4.403553368	4.23E-10	1.49E-09
DHRS13	-1.075573559	2.822756775	1.66E-23	1.30E-22
RHEBL1	-1.075705765	1.258369288	4.49E-12	1.84E-11
SLC7A11	-1.076316008	7.060072619	2.83E-23	2.19E-22
CTH	-1.076358912	4.019240856	5.79E-33	6.60E-32
BUB1B	-1.076930154	5.649492433	4.32E-40	5.99E-39

MARVELD3	-1.077960997	-1.557467649	5.29E-03	8.75E-03
MAP2K3	-1.079890128	7.424561994	6.90E-49	1.23E-47
CYP11A1	-1.082360133	2.933550839	2.12E-19	1.37E-18
CD55	-1.083895368	6.205159012	6.00E-43	9.02E-42
MFAP2	-1.084266794	4.292632106	1.18E-25	1.01E-24
BFSP1	-1.084313166	0.861542519	7.83E-12	3.13E-11
SUGCT	-1.085677173	0.851978735	8.04E-12	3.21E-11
AGTPBP1	-1.086078543	4.524313505	2.48E-24	2.01E-23
STEAP1	-1.086590386	0.997290722	1.41E-10	5.13E-10
MAD2L1	-1.087662409	5.626926123	1.79E-42	2.65E-41
NHLH1	-1.089676667	-0.745134029	3.44E-04	6.73E-04
NCAPD2	-1.089927784	7.508286154	2.34E-57	5.08E-56
ADCY7	-1.090252519	5.816859016	1.89E-52	3.70E-51
MOXD1	-1.090631365	0.840767002	4.08E-09	1.33E-08
TRPV2	-1.09107607	-0.72691634	1.80E-02	2.74E-02
C4orf36	-1.09141625	-0.326671102	1.08E-04	2.24E-04
MAGOHB	-1.091841537	3.129464926	1.03E-19	6.77E-19
TRIP13	-1.091891163	5.013836158	2.81E-42	4.12E-41
EPHB3	-1.093939334	1.739999839	2.36E-15	1.22E-14
TAF1A	-1.094035619	3.113444142	8.94E-19	5.57E-18
SOCS2	-1.094235139	2.154914839	1.54E-18	9.48E-18
OPN1LW	-1.094491346	-0.194090962	1.17E-05	2.73E-05
KCNK5	-1.094938714	-0.776780474	1.58E-04	3.22E-04
CXCL1	-1.095098358	7.104957008	5.25E-60	1.22E-58
FCMR	-1.095207564	1.686492393	5.79E-15	2.91E-14
ZNF593	-1.095309973	4.239273636	2.83E-15	1.45E-14
GNL3	-1.09554991	6.023523223	1.63E-46	2.69E-45
SLC37A4	-1.095613782	6.454597657	1.22E-60	2.90E-59
OLFML2A	-1.096526626	1.607318997	6.40E-17	3.62E-16
LAYN	-1.096996	6.236347756	1.28E-52	2.51E-51
OIP5	-1.097298356	2.664855126	2.50E-26	2.20E-25
CENPF	-1.098339658	7.030276804	6.89E-25	5.73E-24
SLC1A5	-1.100182536	8.632021793	1.17E-57	2.55E-56
RANGRF	-1.100397716	1.558971647	4.34E-13	1.91E-12
PYY	-1.100404151	-0.942454713	1.89E-04	3.80E-04
STMN3	-1.100907225	2.9858312	2.87E-27	2.59E-26
SINHCAF	-1.100919316	5.258582958	1.12E-19	7.35E-19
WNK4	-1.101668587	4.194660798	9.91E-37	1.26E-35
DNAH14	-1.101847634	2.875908902	3.09E-26	2.70E-25
TEAD4	-1.101895514	3.921850995	2.13E-36	2.69E-35
ZBTB42	-1.102985271	2.025338139	6.20E-21	4.30E-20
CDH5	-1.103872864	-1.70709019	6.90E-03	1.12E-02
ALAS2	-1.103904085	-1.486365194	7.16E-03	1.16E-02
GPR3	-1.105022159	2.965897537	8.80E-23	6.66E-22
RASD1	-1.106720678	2.626609573	3.48E-12	1.43E-11
CHRNA3	-1.107026048	-1.592400225	4.79E-03	7.97E-03
RCSD1	-1.107777798	4.646437672	2.00E-45	3.21E-44

GNAL	-1.108083205	-1.436260971	3.44E-03	5.85E-03
CKS1B	-1.108362951	6.580978344	2.45E-48	4.26E-47
MTFR1	-1.109130588	5.073825458	1.50E-31	1.63E-30
DNAJB13	-1.109531354	-1.538910964	4.66E-03	7.76E-03
RUNX2	-1.109839252	3.719298981	7.07E-19	4.43E-18
SORBS1	-1.110283639	1.143175109	9.49E-08	2.75E-07
PPP1R26	-1.11091152	5.339382174	1.82E-22	1.36E-21
INHBE	-1.111711031	-1.897250325	9.57E-03	1.52E-02
SNCG	-1.112041768	-0.513397013	1.90E-05	4.33E-05
CCL24	-1.112799615	-0.43699008	1.50E-04	3.06E-04
GLIS2	-1.113606965	3.75641184	1.64E-23	1.29E-22
ATP2B1	-1.114693286	6.731812869	4.39E-25	3.68E-24
HES6	-1.115424558	1.815563872	2.95E-13	1.31E-12
TRAF5	-1.116786523	3.415950422	3.99E-27	3.59E-26
HES4	-1.117079785	2.846464834	9.69E-09	3.06E-08
TCOF1	-1.118072705	7.632134607	4.65E-44	7.21E-43
C12orf45	-1.119689605	2.722145265	7.51E-23	5.72E-22
TESMIN	-1.119745375	2.642947779	2.14E-22	1.59E-21
PTCH1	-1.120073787	3.091005245	9.80E-31	1.02E-29
FAT3	-1.120492545	3.751231787	6.54E-27	5.84E-26
NUDT8	-1.120707263	1.809460209	2.22E-18	1.35E-17
CDCA5	-1.121269244	7.090626943	4.71E-55	9.84E-54
BCAT2	-1.12209169	6.05696887	4.77E-42	6.93E-41
TNFRSF10D	-1.123105517	4.548855095	8.34E-39	1.12E-37
THOC6	-1.123203204	4.827322297	6.52E-31	6.83E-30
ADARB1	-1.123488868	5.367423572	5.11E-49	9.18E-48
STEAP3	-1.123659001	5.631707312	7.39E-45	1.17E-43
DBNDD1	-1.124037419	4.834614382	2.92E-24	2.37E-23
PPP1R15A	-1.124219837	8.318363569	3.16E-65	8.16E-64
PSMB9	-1.125305591	3.993978794	2.84E-33	3.30E-32
CKS2	-1.126981866	6.288765982	1.74E-57	3.81E-56
TGFB2	-1.126997339	7.235981707	1.24E-41	1.79E-40
TTK	-1.127862229	4.579916711	1.19E-26	1.05E-25
CCDC85B	-1.128644492	4.109823349	4.04E-08	1.21E-07
PBK	-1.130226907	4.791971416	7.86E-39	1.06E-37
ANP32B	-1.132011354	7.16329297	6.77E-60	1.56E-58
PIMREG	-1.134574946	4.157932073	4.54E-41	6.50E-40
PHLDA2	-1.135908124	4.362354259	1.27E-16	7.03E-16
TJP3	-1.136258149	4.847341655	2.77E-26	2.42E-25
PDIK1L	-1.136784872	2.580865039	1.64E-15	8.57E-15
PPP2R3B	-1.136966168	4.554669497	1.47E-30	1.51E-29
TEX30	-1.137815998	2.294119678	1.09E-18	6.74E-18
NAA80	-1.138913288	2.716537111	1.06E-14	5.22E-14
PRELID3A	-1.140276742	1.893259018	2.46E-21	1.74E-20
TMTC4	-1.140277779	1.352460703	2.24E-14	1.08E-13
NECTIN1	-1.140907542	3.545684058	2.09E-25	1.77E-24
SLC19A2	-1.141107168	3.732017477	4.34E-14	2.06E-13

H2BC17	-1.141112043	0.883078619	5.68E-08	1.68E-07
FMNL2	-1.14130493	5.017760376	2.37E-23	1.85E-22
FRMD4A	-1.141650871	4.270749688	3.86E-41	5.53E-40
PDPN	-1.141895535	0.344977806	3.80E-09	1.24E-08
C6orf52	-1.142346668	2.584370499	8.07E-26	6.95E-25
CKAP2L	-1.142636695	5.51476837	1.37E-34	1.65E-33
ING1	-1.143556207	1.967230269	5.12E-18	3.06E-17
CDC25C	-1.145144899	3.895223516	9.65E-43	1.44E-41
ZNF850	-1.145684184	4.239016414	6.91E-42	1.00E-40
BHLHA15	-1.145798695	0.018790232	2.58E-07	7.20E-07
MISP3	-1.14581783	-0.208447769	3.89E-06	9.56E-06
ABLIM2	-1.147189317	1.042657977	4.13E-09	1.34E-08
GPC2	-1.147898094	-0.819621682	4.87E-04	9.35E-04
CCNB1	-1.148841803	7.294685093	3.78E-73	1.13E-71
ACTA2	-1.149158055	1.743064262	5.35E-18	3.20E-17
TMEM81	-1.149355565	1.278362482	4.11E-11	1.55E-10
GPR85	-1.149375924	1.888180876	3.44E-20	2.31E-19
FGFRL1	-1.149770766	6.237150742	2.64E-41	3.80E-40
GJC1	-1.150529728	-0.77734901	6.30E-05	1.34E-04
CHD5	-1.150671851	-0.297428435	1.83E-05	4.17E-05
DUOXA1	-1.152042341	-0.675317446	2.95E-04	5.80E-04
RAD54L	-1.152937574	4.360204535	1.61E-49	2.95E-48
PLCXD1	-1.153414668	6.04611807	4.71E-59	1.05E-57
PKN3	-1.154550836	5.165967046	1.43E-37	1.85E-36
PTPRN2	-1.154580271	3.567031911	7.91E-30	7.90E-29
ARG2	-1.154849873	3.806067823	6.55E-39	8.84E-38
GKAP1	-1.155427499	1.745444224	6.42E-18	3.83E-17
NPC1	-1.156210967	7.859928662	3.12E-64	7.88E-63
ADAMTS9	-1.156997319	4.370784362	5.58E-46	9.03E-45
CCNO	-1.157533621	3.142528748	4.86E-17	2.77E-16
SMIM3	-1.158900781	2.441546177	5.42E-20	3.59E-19
SMURF2	-1.15976005	7.26435525	1.19E-39	1.63E-38
H1-4	-1.160625799	2.944157973	1.80E-20	1.22E-19
FOXF2	-1.161503646	2.486321775	1.39E-21	9.95E-21
EPHB4	-1.162220426	6.154279458	1.35E-41	1.95E-40
ARID3B	-1.162660186	4.087649954	7.78E-45	1.23E-43
RNF208	-1.163172129	0.310520242	8.11E-09	2.58E-08
IGFBP1	-1.164083115	2.9953376	4.07E-33	4.67E-32
TBC1D8	-1.16548946	2.585596941	1.76E-23	1.38E-22
CORO2B	-1.165949848	3.463941299	1.20E-31	1.30E-30
CAMK1	-1.166422693	3.475962682	2.73E-28	2.58E-27
PYGM	-1.166858097	-0.195696768	5.51E-06	1.33E-05
LRFN4	-1.16888902	5.337026304	1.39E-18	8.54E-18
C15orf39	-1.169379735	5.204943135	2.50E-29	2.46E-28
SSTR2	-1.1700864	-1.013429692	3.95E-04	7.66E-04
TMPRSS5	-1.170319572	-1.453557689	2.25E-03	3.95E-03
SPAG5	-1.170910282	6.754504224	2.38E-71	6.93E-70

KIFC3	-1.171951762	7.366972853	1.38E-49	2.52E-48
LRP8	-1.172035714	5.93007545	1.67E-46	2.75E-45
MYOM1	-1.172935983	-1.610815524	3.60E-03	6.11E-03
FERMT2	-1.17295644	6.11327515	3.04E-36	3.81E-35
PSRC1	-1.17412451	4.696246053	2.95E-48	5.13E-47
PLK1	-1.174334801	6.040857557	6.23E-46	1.01E-44
NAT16	-1.17497846	-1.167721861	3.84E-03	6.49E-03
ALDH6A1	-1.175150021	3.668453544	9.02E-41	1.28E-39
TUBE1	-1.175548591	2.750826578	2.66E-22	1.98E-21
HOXB5	-1.175652169	-1.252521543	6.23E-04	1.18E-03
CFAP45	-1.177897617	1.052145538	2.84E-10	1.02E-09
TAMM41	-1.179382157	3.013332354	2.91E-31	3.10E-30
LMNB1	-1.180344886	5.851631168	1.64E-42	2.44E-41
MEX3B	-1.180504698	0.742668987	1.70E-12	7.16E-12
PLCXD2	-1.181136432	1.267483502	6.56E-10	2.28E-09
PFAS	-1.181451109	5.958061511	3.72E-59	8.39E-58
SLC25A19	-1.182183603	2.146666932	1.38E-16	7.64E-16
SYPL2	-1.182317924	-1.075120056	3.44E-04	6.73E-04
PCK2	-1.183071515	5.975376689	7.48E-54	1.51E-52
CDCA3	-1.185574532	5.111964458	5.05E-48	8.72E-47
PDCD2L	-1.185855236	3.903592919	1.56E-37	2.01E-36
EFHD1	-1.185928173	3.023926302	1.25E-33	1.47E-32
C1QTNF12	-1.187003931	-0.696840855	6.99E-05	1.48E-04
RSL24D1	-1.187253483	5.958273475	5.35E-58	1.18E-56
FAM160A1	-1.187315622	3.598447117	1.95E-38	2.60E-37
KIF13A	-1.18773644	5.611761887	5.26E-34	6.23E-33
ETV4	-1.188029539	5.685915527	2.44E-44	3.82E-43
HAUS4	-1.189438563	4.916923193	1.03E-43	1.59E-42
NOTCH3	-1.190354916	1.660504644	1.86E-16	1.02E-15
C1QL1	-1.190878277	-1.389021248	9.67E-04	1.78E-03
BEND3	-1.191160159	3.192489568	2.57E-26	2.26E-25
HVCN1	-1.191258471	2.353743712	4.55E-22	3.35E-21
THBS1	-1.19200386	12.80359124	1.32E-63	3.30E-62
SLC25A29	-1.193103425	4.548097165	1.75E-37	2.25E-36
LMO4	-1.193119454	3.755935448	2.81E-28	2.64E-27
WT1	-1.194190453	2.706756483	2.15E-23	1.68E-22
CDC25A	-1.194787897	5.082724425	1.44E-48	2.54E-47
HK2	-1.1964837	6.174033166	6.79E-52	1.31E-50
PDE9A	-1.198796387	1.93452477	7.99E-24	6.36E-23
H2BC11	-1.200719763	1.100612095	1.10E-13	5.04E-13
P2RY2	-1.203776102	3.898924682	5.54E-46	8.98E-45
GSDME	-1.20474577	5.504302819	5.74E-57	1.24E-55
PDE7B	-1.205419278	1.477641149	6.53E-15	3.27E-14
TAGLN	-1.205908742	5.889918531	1.08E-57	2.37E-56
TACSTD2	-1.205975949	5.113293361	5.30E-40	7.31E-39
FBL	-1.206385076	7.462604045	8.34E-58	1.83E-56
BTG2	-1.206563061	2.946399572	5.77E-36	7.19E-35

CSGALNACT1	-1.208879559	0.297883631	3.01E-08	9.11E-08
AIG1	-1.209078829	4.958769413	2.86E-49	5.19E-48
SNORC	-1.213121146	-1.420708201	2.36E-03	4.12E-03
RCN1	-1.213768292	7.853508309	1.02E-81	3.48E-80
PINLYP	-1.215041492	0.587931335	4.26E-11	1.61E-10
CMSS1	-1.215168583	4.685900257	1.89E-48	3.30E-47
BLMH	-1.216486231	5.492778053	4.15E-60	9.66E-59
HEY1	-1.217470954	-0.735864908	1.37E-05	3.18E-05
FOXA3	-1.217624282	-1.049190065	3.56E-03	6.05E-03
CDCA4	-1.217903343	5.745814043	9.92E-35	1.20E-33
MND1	-1.217969501	3.305056627	1.31E-40	1.85E-39
ZIC2	-1.218380841	1.660315843	1.83E-19	1.18E-18
WDR62	-1.21887012	6.23255333	8.97E-45	1.42E-43
UBASH3B	-1.219211302	5.748401034	1.30E-37	1.69E-36
TEX14	-1.219949001	-0.636987627	1.24E-05	2.88E-05
DGKE	-1.220137756	0.92856588	4.09E-11	1.55E-10
ADAT3	-1.22019627	-0.325069836	5.79E-05	1.24E-04
CCDC163	-1.220963899	1.289793963	2.59E-16	1.41E-15
HMMR	-1.221198111	1.971907819	7.39E-23	5.62E-22
NUAK2	-1.221682272	5.201016338	6.25E-60	1.45E-58
CDH4	-1.223501665	7.766623767	1.73E-59	3.91E-58
COL4A1	-1.223575659	6.765903189	1.98E-64	5.01E-63
FBLN2	-1.224039907	-1.767369548	3.29E-03	5.62E-03
GJA3	-1.226890552	0.785016783	1.02E-09	3.49E-09
ETS2	-1.230513195	6.340552666	1.65E-59	3.76E-58
TYMSOS	-1.233248493	-0.547088586	1.19E-05	2.77E-05
NME7	-1.233825394	4.643815484	3.27E-42	4.78E-41
GCSH	-1.233959014	3.589662053	4.08E-35	4.95E-34
PLAAT3	-1.23461254	5.080765928	2.25E-46	3.69E-45
EGFR	-1.238674287	7.64696215	2.19E-48	3.82E-47
ADGRE5	-1.23903504	7.609215317	1.41E-60	3.35E-59
KCTD19	-1.241053652	0.15045225	1.60E-06	4.12E-06
RAPGEF3	-1.242979225	1.05435543	1.03E-12	4.41E-12
H3C3	-1.243752873	-0.166093699	1.75E-06	4.48E-06
LOXL1	-1.244039647	2.769212464	3.69E-20	2.47E-19
FLI1	-1.244061592	4.281623755	1.18E-35	1.46E-34
SCARF1	-1.244218068	2.014672871	1.59E-21	1.14E-20
S100A3	-1.244744773	0.392999188	1.09E-06	2.86E-06
ACHE	-1.24976338	-0.030299699	8.99E-08	2.61E-07
CCL26	-1.254003878	1.402813512	4.99E-17	2.84E-16
OCLN	-1.254540594	3.157712841	1.26E-30	1.30E-29
E2F7	-1.254793484	6.132958147	2.87E-24	2.32E-23
PIGL	-1.256044629	2.541150686	1.43E-26	1.26E-25
AOAH	-1.256142513	-1.11484146	1.10E-04	2.28E-04
PRKCA	-1.257046387	5.693856264	6.73E-56	1.43E-54
CACNG6	-1.257466645	0.786984677	7.22E-12	2.90E-11
KIF2C	-1.258163824	6.910532706	1.71E-82	5.96E-81

PFDN2	-1.258801247	6.085032368	4.09E-54	8.35E-53
EVA1A	-1.258907159	4.189426855	9.36E-45	1.48E-43
GDF11	-1.258914111	3.72720266	1.01E-47	1.73E-46
FZD8	-1.259579796	0.767052914	3.06E-10	1.09E-09
PHF10	-1.259620496	5.020956915	7.07E-46	1.14E-44
GNG13	-1.261416821	-1.126813315	3.72E-03	6.30E-03
GUCA1B	-1.261599627	1.681815294	2.98E-21	2.10E-20
IL23A	-1.261747397	1.726211496	1.92E-17	1.12E-16
PLPP2	-1.264227383	5.638295909	8.29E-50	1.53E-48
NECAB2	-1.264761181	0.016640616	6.56E-08	1.93E-07
DLGAP5	-1.26721309	6.742207593	1.83E-48	3.20E-47
CLIP2	-1.267417819	4.845497788	1.07E-33	1.26E-32
CYP24A1	-1.268057203	4.581941514	1.20E-33	1.41E-32
LIMCH1	-1.268088121	7.833582094	1.04E-66	2.78E-65
SLC30A3	-1.268315605	1.476698248	8.62E-13	3.72E-12
HMGB3	-1.270297368	5.169658316	4.33E-59	9.71E-58
AFAP1L1	-1.270900997	3.698312473	1.32E-45	2.12E-44
NPTXR	-1.271417795	3.398422974	4.29E-38	5.68E-37
TRAP1	-1.275843969	6.531472443	4.45E-73	1.32E-71
SOWAHD	-1.275897392	-1.874830807	3.02E-03	5.17E-03
PHF19	-1.277757991	6.529153756	4.97E-66	1.31E-64
NTN3	-1.278823145	-1.340524413	6.93E-03	1.12E-02
EXOSC7	-1.279215508	4.275334751	8.15E-38	1.07E-36
FANCD2	-1.280641238	3.786854808	1.88E-27	1.72E-26
ENO3	-1.280952694	2.336288457	1.20E-31	1.30E-30
TNS1	-1.280958754	1.526195609	2.82E-21	1.99E-20
GPR173	-1.282180837	2.636436185	2.54E-32	2.83E-31
EXTL1	-1.283004356	-0.861641168	3.64E-05	8.01E-05
FAM189A2	-1.285983011	0.323809131	2.68E-09	8.91E-09
GALNT14	-1.28639481	3.208707358	4.00E-35	4.86E-34
DENND11	-1.286692138	4.591418036	1.68E-43	2.55E-42
TMC5	-1.287016894	0.584017601	3.30E-12	1.36E-11
TGFBR3L	-1.28777042	1.972283003	5.07E-10	1.78E-09
CTNNBIP1	-1.287865605	4.063399465	1.19E-48	2.11E-47
TCTE3	-1.287873128	-1.327473145	4.13E-04	8.00E-04
B3GNT5	-1.287927967	3.963996461	6.32E-23	4.83E-22
KCNJ14	-1.291449691	3.627222583	5.79E-27	5.18E-26
OAF	-1.293576341	6.589781998	6.20E-59	1.38E-57
GPR65	-1.295722155	3.934400043	5.92E-28	5.51E-27
AC007405.4	-1.297010264	1.451711942	2.89E-14	1.39E-13
ERICH2	-1.297010264	1.451711942	2.89E-14	1.39E-13
INKA2	-1.2981232	2.463626546	2.00E-33	2.34E-32
TSPAN12	-1.298123214	1.06145077	9.98E-15	4.94E-14
LYL1	-1.298755404	1.24869224	2.38E-14	1.15E-13
BIRC5	-1.299363845	6.525560333	5.87E-62	1.43E-60
CCNA2	-1.299393871	5.998697751	8.75E-60	2.01E-58
LHX3	-1.299616888	-1.001811251	2.47E-04	4.90E-04

PRRT4	-1.301569252	-1.419311116	6.42E-04	1.22E-03
TRPC4	-1.303475085	-1.420875778	3.64E-04	7.08E-04
MAN1C1	-1.305015017	-0.236309764	9.83E-07	2.59E-06
TENT5B	-1.307934228	3.474886261	7.95E-23	6.04E-22
TMEM45B	-1.310216493	-0.521537707	3.19E-06	7.91E-06
PLEKHA6	-1.310331172	4.96744098	6.12E-57	1.32E-55
HMGB2	-1.310616256	6.424882739	2.54E-57	5.50E-56
SH2D5	-1.31145507	4.62819021	4.20E-45	6.68E-44
KCNS1	-1.314387283	1.098808783	7.75E-14	3.61E-13
ADAMTS6	-1.314985256	6.481091902	1.93E-44	3.02E-43
SCUBE2	-1.31511087	-0.920145127	1.64E-05	3.76E-05
FMC1	-1.316335742	0.514409714	5.51E-13	2.41E-12
COL13A1	-1.316534846	6.364265821	4.55E-66	1.20E-64
ASGR1	-1.316601089	0.206177062	1.63E-09	5.50E-09
APOE	-1.317093992	-0.918866026	2.78E-05	6.21E-05
RAB20	-1.319167197	1.391317072	3.48E-17	2.00E-16
CDC42EP5	-1.319524264	2.096800405	3.27E-14	1.56E-13
CEBPD	-1.319628674	2.779572014	3.25E-12	1.34E-11
BUB1	-1.319687535	6.978871561	8.08E-55	1.68E-53
PCCA	-1.321373257	0.762959649	3.25E-11	1.24E-10
UPP1	-1.321856554	7.265444735	9.38E-61	2.24E-59
PIF1	-1.32203222	3.162237007	4.37E-30	4.40E-29
CITED4	-1.323155875	-1.113064241	2.00E-04	4.02E-04
EXOSC8	-1.324103517	5.333374352	5.60E-49	1.00E-47
PRRT1	-1.327361835	0.275682567	7.68E-12	3.08E-11
SHMT2	-1.329099445	7.764644716	2.13E-78	6.98E-77
PRSS3	-1.329937832	2.961699322	2.01E-38	2.68E-37
KIF17	-1.333026927	1.739677491	1.95E-15	1.01E-14
KIF18B	-1.335458721	6.060649623	1.67E-77	5.42E-76
NUF2	-1.33833729	4.694958517	1.66E-48	2.91E-47
BCL2	-1.338421634	2.032801606	2.08E-18	1.26E-17
AP006333.1	-1.339631561	0.335115202	9.36E-12	3.72E-11
TEX19	-1.339756132	-1.629696264	1.93E-03	3.41E-03
RGS3	-1.343541871	5.213878278	1.19E-66	3.19E-65
MRM1	-1.344471603	3.212029084	4.71E-25	3.93E-24
PRDM7	-1.34528549	-1.629403	6.47E-04	1.22E-03
BCL7A	-1.349367424	3.656518295	5.74E-38	7.55E-37
STARD8	-1.350212571	5.124300389	9.51E-47	1.58E-45
PTTG1	-1.351360873	6.403983451	2.80E-75	8.67E-74
KCNS3	-1.351710188	0.117089315	7.14E-10	2.47E-09
EPAS1	-1.354607813	6.667330214	3.38E-87	1.27E-85
ZNF467	-1.355411814	-1.239505275	1.41E-04	2.89E-04
ZNF697	-1.357434546	5.004509502	3.19E-43	4.84E-42
HIC1	-1.358559247	0.741389446	1.11E-09	3.78E-09
IRF5	-1.359158155	3.706542513	1.73E-55	3.63E-54
SCML2	-1.360526969	2.581630046	4.05E-21	2.84E-20
TFAP4	-1.3616374	3.142686401	2.81E-47	4.76E-46

TMEM51	-1.362313201	3.981542663	2.56E-53	5.10E-52
NPM3	-1.362517147	5.266678513	1.26E-54	2.60E-53
TROAP	-1.36261605	4.842680037	2.50E-58	5.52E-57
DUSP2	-1.362904045	0.285202247	5.49E-10	1.92E-09
PAPLN	-1.362952604	1.460399065	6.89E-16	3.67E-15
HMSD	-1.364199753	1.632483054	2.43E-25	2.05E-24
MN1	-1.366152155	-1.556846552	5.28E-04	1.01E-03
PGM5	-1.369278663	-1.089834773	1.37E-05	3.17E-05
TMEM190	-1.371686248	-1.127832065	6.62E-05	1.41E-04
SCO2	-1.37284918	-0.776145804	3.40E-04	6.64E-04
F2RL1	-1.374701915	5.963318722	1.73E-47	2.95E-46
MAMDC2	-1.377559587	5.923411032	1.34E-52	2.63E-51
KRT33B	-1.383532553	0.862551305	3.04E-08	9.20E-08
DNMT3B	-1.38393306	3.396179658	1.25E-46	2.07E-45
SH2B2	-1.385565632	1.2893744	1.01E-15	5.30E-15
SUPT3H	-1.389459791	2.817389935	1.67E-30	1.71E-29
BCAS1	-1.389820136	-0.087173488	3.22E-10	1.15E-09
NEURL1	-1.39065227	0.215608835	9.43E-12	3.75E-11
NLRP12	-1.391114474	-0.412022149	3.73E-08	1.12E-07
F2R	-1.391825184	4.178951173	2.42E-55	5.07E-54
IL12A	-1.392320454	1.662849811	1.32E-16	7.32E-16
PPP1R32	-1.39267901	-0.830225749	1.31E-06	3.41E-06
LYAR	-1.393233269	5.14458397	1.74E-65	4.52E-64
NHSL1	-1.393377708	2.584637059	6.82E-29	6.56E-28
LMX1B	-1.393746599	1.139236586	1.43E-13	6.50E-13
HS3ST3A1	-1.394086367	3.102014903	3.72E-34	4.42E-33
EML1	-1.394170698	0.596136792	7.54E-12	3.02E-11
RAB38	-1.405185481	-1.470532576	1.99E-04	3.99E-04
KCNK12	-1.405969061	-0.46085426	9.21E-08	2.67E-07
CELF4	-1.406539252	-1.592238692	3.89E-04	7.56E-04
SYNPO	-1.406722246	4.430768449	1.53E-32	1.72E-31
C1orf21	-1.407067983	2.978949995	1.14E-42	1.69E-41
C10orf67	-1.408670453	-0.029641987	1.19E-07	3.41E-07
CTNNAL1	-1.412830298	6.980869719	3.84E-85	1.40E-83
LRIG3	-1.413133878	2.169122878	1.51E-15	7.91E-15
SLC43A3	-1.413142732	7.256038604	1.13E-100	5.23E-99
UBE2C	-1.413621278	6.697213921	6.80E-77	2.18E-75
SNCAIP	-1.414860475	0.943927348	2.01E-16	1.10E-15
C1orf115	-1.415193962	-0.637006268	1.60E-06	4.13E-06
VWA5B2	-1.415856399	-0.285895253	6.39E-08	1.88E-07
HAAO	-1.416090054	-0.776426562	6.82E-07	1.83E-06
SCARA3	-1.419532271	2.880681041	5.22E-41	7.45E-40
KCNC4	-1.42181074	2.080449983	5.55E-28	5.16E-27
ALPK2	-1.423123851	4.816181847	1.34E-68	3.73E-67
PACSIN1	-1.4271228	-0.965362373	2.00E-06	5.08E-06
NTN4	-1.429750478	1.736949972	2.29E-14	1.11E-13
ARHGAP11B	-1.432695653	3.268593595	4.32E-36	5.40E-35

CPT1A	-1.434046198	5.903735618	1.44E-79	4.78E-78
RTN4R	-1.435949437	2.785364965	1.42E-22	1.07E-21
TYRO3	-1.436239117	5.250769769	3.27E-69	9.26E-68
ADM2	-1.440699708	2.637379478	1.92E-31	2.06E-30
PPARGC1B	-1.441595617	3.271003444	1.23E-28	1.17E-27
PPIF	-1.442154743	8.048081358	1.94E-105	9.50E-104
MUSK	-1.44433908	-0.62823399	8.52E-05	1.79E-04
TFAP2E	-1.444865029	1.634809932	3.58E-28	3.35E-27
METTL27	-1.446259508	1.718609254	6.85E-15	3.43E-14
LONRF1	-1.447506163	3.685082953	4.12E-32	4.56E-31
ADGRE2	-1.450854309	2.688449998	3.98E-30	4.01E-29
SMAD3	-1.452631365	6.8136684	8.86E-78	2.88E-76
IGDCC4	-1.453405582	4.023285083	5.94E-66	1.56E-64
EFEMP1	-1.45369707	5.636786313	2.60E-62	6.36E-61
TIE1	-1.454000698	-0.647653608	4.93E-07	1.34E-06
TFPI2	-1.458835525	4.758198063	1.54E-66	4.13E-65
AEBP1	-1.461399997	-0.452759382	3.32E-07	9.18E-07
CGB7	-1.463165616	1.245554364	1.87E-16	1.03E-15
IL17RE	-1.46415219	2.106477979	3.25E-20	2.18E-19
NEK2	-1.468467454	4.212370305	6.76E-48	1.17E-46
ITPKA	-1.47128962	2.021351453	2.96E-24	2.40E-23
EVA1C	-1.478283511	1.755892328	7.26E-20	4.79E-19
TULP2	-1.480708065	-1.611380126	2.45E-04	4.86E-04
ATP6V1B1	-1.482208795	-0.093403204	2.39E-10	8.60E-10
LNP1	-1.483087532	-0.807570972	6.76E-07	1.81E-06
CHN1	-1.490423023	3.291593671	2.11E-54	4.34E-53
TMEM221	-1.491424683	-1.10254843	3.71E-06	9.15E-06
CNTNAP1	-1.49327668	4.384282614	2.68E-73	8.02E-72
MDFI	-1.494373736	3.361859224	3.02E-34	3.60E-33
IL17D	-1.49597001	-1.19512525	8.52E-06	2.02E-05
HOXC9	-1.496715969	-1.194275986	2.57E-05	5.77E-05
FGD2	-1.499525147	-1.296011285	4.13E-05	9.02E-05
EIF4EBP1	-1.500152601	6.742717557	1.59E-65	4.12E-64
IFNLR1	-1.500743102	2.632899862	1.11E-36	1.41E-35
HS3ST3B1	-1.501450935	6.017865736	3.15E-95	1.33E-93
ZNF483	-1.502367137	-0.648389136	1.35E-07	3.86E-07
DUSP15	-1.504467024	-1.357033778	1.75E-04	3.54E-04
RASIP1	-1.505639761	0.352201508	5.64E-12	2.29E-11
PDGFA	-1.509082725	3.751249535	1.89E-62	4.63E-61
TNFRSF21	-1.511873406	6.458124081	1.98E-106	9.82E-105
HTR7	-1.512237482	1.956073965	4.58E-34	5.44E-33
KAZALD1	-1.515355607	1.744748006	5.57E-30	5.58E-29
RDH16	-1.515676749	-1.037321005	2.00E-06	5.08E-06
BDH1	-1.517705435	4.955056218	1.46E-65	3.79E-64
CGB3	-1.520911206	-1.522208777	9.18E-05	1.92E-04
AKR1C1	-1.523467046	0.575427886	4.65E-16	2.50E-15
KREMEN2	-1.523527972	1.040655964	5.26E-10	1.84E-09

APOC1	-1.52609851	1.403309085	1.01E-20	6.96E-20
H4C2	-1.527204309	-1.404302541	4.78E-04	9.19E-04
SLC25A33	-1.527411807	3.530435569	7.50E-46	1.21E-44
TNFSF9	-1.527927882	3.054797654	1.81E-49	3.31E-48
HSPA5	-1.530538641	11.65096252	8.55E-141	6.84E-139
NOD1	-1.532155215	2.656202114	3.42E-49	6.17E-48
HSPA4L	-1.532469073	4.065050895	2.87E-45	4.57E-44
ADTRP	-1.532898118	0.369012728	2.74E-13	1.22E-12
MAFA	-1.532987137	-0.73547541	5.36E-08	1.59E-07
FGFR3	-1.536614704	1.879268181	3.42E-20	2.30E-19
CATSPERZ	-1.537075225	1.850838416	3.23E-25	2.72E-24
CENPH	-1.537086161	2.756142134	6.52E-41	9.27E-40
TERT	-1.539208995	0.387962516	7.03E-15	3.51E-14
HAS2	-1.539280217	4.645377188	6.45E-47	1.08E-45
HECW2	-1.541693995	2.450459353	1.32E-18	8.16E-18
CGB8	-1.542155412	-1.575264307	1.01E-04	2.11E-04
ZNF165	-1.542800634	3.810956392	1.27E-68	3.55E-67
RELN	-1.544906234	2.713519033	6.08E-32	6.67E-31
CGB2	-1.547196831	-1.505148517	7.78E-05	1.64E-04
FAM167A	-1.548245856	1.523748437	6.70E-25	5.57E-24
TNFRSF18	-1.551691603	-0.047473214	3.96E-09	1.29E-08
EEPD1	-1.552606508	2.521839577	9.31E-22	6.72E-21
JAG1	-1.552712323	4.509536625	3.82E-54	7.80E-53
COL20A1	-1.552877762	1.78955112	3.59E-17	2.06E-16
ASAP3	-1.555606281	3.943625057	1.73E-64	4.39E-63
LAG3	-1.555870828	-0.797163236	2.93E-07	8.14E-07
CES3	-1.558800287	0.486264539	9.99E-17	5.59E-16
EID3	-1.560026526	-1.154402082	2.83E-06	7.07E-06
PLIN2	-1.560682348	5.391333118	7.96E-99	3.58E-97
ITGB1BP2	-1.561041807	-1.373647703	3.36E-05	7.42E-05
GYPC	-1.564081242	2.012231209	7.68E-28	7.11E-27
IKZF3	-1.565575732	0.333692644	9.32E-13	4.00E-12
STK17B	-1.568868486	4.005111414	5.43E-47	9.13E-46
NRTN	-1.569397174	-0.872085346	2.78E-05	6.21E-05
S100A5	-1.575108497	-0.403163358	4.73E-10	1.66E-09
GJB2	-1.575995241	-0.307656554	4.52E-08	1.35E-07
SPON2	-1.579153397	-0.055038703	1.32E-10	4.81E-10
EVPL	-1.581946548	5.235336652	4.68E-32	5.16E-31
IGFBPL1	-1.582794793	5.467606006	7.55E-102	3.55E-100
TENM2	-1.583249659	7.91790676	1.13E-118	6.67E-117
DDIT3	-1.586121406	5.192317342	3.81E-76	1.21E-74
VASH2	-1.587105385	-0.555505454	1.37E-09	4.65E-09
PDE4D	-1.589527525	2.764364478	8.02E-18	4.77E-17
SP5	-1.589914391	0.556384713	3.95E-14	1.87E-13
CDK15	-1.591369274	0.055710759	1.12E-12	4.78E-12
GSR	-1.591939072	4.724471701	1.94E-64	4.91E-63
ROR1	-1.594798677	3.718810188	2.14E-33	2.49E-32

FAM72A	-1.596493012	5.097766292	8.01E-59	1.78E-57
BBC3	-1.601337581	1.816855503	3.96E-29	3.84E-28
PRG2	-1.603950679	-1.026059179	7.20E-07	1.93E-06
MMP10	-1.610306732	-0.101744686	1.51E-09	5.11E-09
GPR68	-1.618181578	2.435485741	6.64E-42	9.63E-41
ARHGAP11A	-1.62067632	6.053575056	1.22E-116	6.98E-115
NGFR	-1.621689131	-1.592890093	4.34E-05	9.47E-05
SRRM3	-1.622445899	2.196315621	2.84E-39	3.85E-38
DDX39B	-1.622723082	-0.042526329	2.06E-12	8.60E-12
SOCS3	-1.625371521	4.451630598	1.10E-71	3.21E-70
TRPV4	-1.627341409	-0.1572016	9.16E-10	3.14E-09
MIF4GD	-1.627934934	3.766375009	1.27E-56	2.72E-55
ADRB2	-1.628661314	3.441690347	6.47E-32	7.09E-31
CCNB1IP1	-1.629237046	5.200223154	1.52E-93	6.24E-92
CXCL3	-1.629775396	2.836469487	2.06E-30	2.11E-29
FAM72D	-1.631126243	5.013626395	1.42E-58	3.16E-57
PPARG	-1.631264861	3.797628916	7.14E-70	2.05E-68
MSH5	-1.631362685	-1.504995753	3.07E-04	6.03E-04
FAM72B	-1.632601478	4.983243279	1.07E-68	2.99E-67
MACROD1	-1.633511741	4.198451697	3.33E-50	6.20E-49
TMEM158	-1.636110042	3.454291627	1.84E-58	4.09E-57
UGCG	-1.636243448	7.253433236	9.13E-50	1.69E-48
TSPAN33	-1.636599392	1.842881875	2.01E-29	1.98E-28
RND1	-1.636859415	0.623579922	1.56E-18	9.59E-18
CNIH3	-1.637610507	1.801163635	2.01E-27	1.83E-26
ESRP1	-1.64062013	-0.520278825	6.82E-09	2.18E-08
PTCD1	-1.640999999	-1.989567533	6.96E-04	1.31E-03
PRDM11	-1.641239673	2.499012846	9.26E-32	1.01E-30
NPAS2	-1.644336025	5.507001961	3.00E-106	1.48E-104
CRIP2	-1.644488196	1.724932655	2.31E-17	1.34E-16
CDC20	-1.644613978	7.636577907	2.53E-98	1.12E-96
FAM131C	-1.644680742	0.416695895	1.53E-14	7.48E-14
FAM72C	-1.645090362	5.123988717	1.04E-64	2.67E-63
NT5M	-1.64651835	1.137548152	1.82E-18	1.12E-17
CENPV	-1.648055628	4.582987689	3.93E-95	1.66E-93
PWWP2B	-1.651618927	3.037258522	3.21E-26	2.80E-25
MCTP1	-1.652621508	2.540682708	4.57E-28	4.27E-27
FAM81A	-1.657289852	1.370582282	9.51E-18	5.63E-17
IER3	-1.658476701	9.8123514	3.19E-94	1.33E-92
EGR3	-1.659196854	-0.851195799	2.78E-08	8.44E-08
TCF7L1	-1.663225601	0.61990667	1.24E-14	6.10E-14
ADAM11	-1.664064037	2.063764139	5.04E-36	6.29E-35
ATF3	-1.665755059	5.017464194	4.05E-76	1.28E-74
TSPOAP1	-1.666272758	3.356757441	2.50E-61	6.03E-60
RHOV	-1.666527929	0.014162225	1.37E-12	5.77E-12
FIBCD1	-1.670663952	1.034637121	5.81E-15	2.91E-14
HSPA12A	-1.674002949	2.638756392	4.77E-47	8.04E-46

NME1-NME2	-1.67591279	-1.238104991	1.68E-06	4.32E-06
ZNF704	-1.676194552	-1.873567625	3.11E-04	6.10E-04
TMEM121	-1.676664924	-1.237725023	9.12E-07	2.41E-06
ANO2	-1.67858001	1.4938274	7.13E-24	5.69E-23
DOP1B	-1.687539114	5.073027847	3.28E-82	1.13E-80
ARMC12	-1.689327584	0.75521647	3.70E-19	2.36E-18
LGALSL	-1.692085062	3.62586536	1.60E-63	3.98E-62
SMCO2	-1.693225993	-1.40409571	4.68E-05	1.02E-04
THRB	-1.69796832	-1.222872682	7.49E-07	2.00E-06
ZCCHC18	-1.69884674	-1.282143097	9.60E-06	2.26E-05
PKDCC	-1.70030437	0.378128822	2.10E-18	1.27E-17
GPR161	-1.700891119	4.334082959	1.69E-95	7.19E-94
ARL2	-1.701678836	5.272927237	3.53E-71	1.03E-69
WNT10B	-1.703784035	1.317559411	3.63E-22	2.69E-21
CSF2	-1.717847481	6.646798216	2.92E-130	2.06E-128
SLC43A1	-1.722961035	3.821280754	9.72E-85	3.51E-83
CGB5	-1.728183426	-1.389570291	6.37E-06	1.53E-05
SEPTIN4	-1.731740347	-0.989780378	4.15E-08	1.24E-07
PTP4A3	-1.734662146	3.714517703	1.85E-53	3.71E-52
PPP2R2C	-1.736879084	0.425945727	9.28E-18	5.50E-17
SH2D2A	-1.739189047	2.0574261	2.26E-22	1.68E-21
N4BP3	-1.739674825	-0.164419209	6.32E-11	2.35E-10
COL8A1	-1.739740303	8.051595423	1.16E-89	4.52E-88
EGR1	-1.745990653	5.398690354	6.05E-114	3.34E-112
KCNJ4	-1.747363678	0.083027732	3.77E-09	1.23E-08
EPGN	-1.750601845	-0.705777455	9.19E-10	3.16E-09
ALDH1A3	-1.75237298	2.95292896	2.01E-61	4.85E-60
TMEM200B	-1.758194608	4.167042983	5.27E-61	1.26E-59
IL1RAPL1	-1.763262319	3.334418512	9.57E-29	9.16E-28
DLG2	-1.768795436	-0.953374967	7.44E-07	1.99E-06
NPR1	-1.769782684	-0.425637351	5.46E-11	2.04E-10
CMTM8	-1.770446348	1.206089292	1.17E-23	9.24E-23
LRRC73	-1.772732542	-1.296280459	1.95E-06	4.96E-06
HOXB8	-1.775617202	-1.726815911	2.16E-05	4.89E-05
TTC9B	-1.776554137	-1.419729863	4.91E-06	1.19E-05
BEAN1	-1.777233878	-0.228407096	2.21E-12	9.19E-12
MERTK	-1.77937541	1.951037524	7.08E-37	9.04E-36
BEND7	-1.784114693	4.246966456	1.73E-89	6.74E-88
ANKRD33B	-1.785170775	4.619823755	2.28E-92	9.15E-91
SCT	-1.785469475	-1.222672653	3.83E-07	1.05E-06
ADAMTSL1	-1.786591787	3.837747118	1.79E-92	7.22E-91
CDH13	-1.786961936	-0.485202349	7.68E-11	2.84E-10
FNDC5	-1.789414803	-0.338563521	1.68E-09	5.67E-09
PEAR1	-1.794589497	3.419873628	9.34E-54	1.89E-52
CFP	-1.799817786	-0.509747248	2.09E-10	7.56E-10
ODC1	-1.801817278	6.81954727	5.90E-128	4.02E-126
GCNT4	-1.803287413	-0.243161572	7.32E-11	2.71E-10

AASS	-1.805701744	4.026517261	2.74E-75	8.50E-74
DUSP5	-1.807205985	6.17405235	6.04E-141	4.86E-139
DNER	-1.816138516	0.868165372	5.21E-21	3.64E-20
PLXND1	-1.81646352	5.345266922	1.08E-74	3.29E-73
AURKB	-1.816874534	6.098913113	9.74E-130	6.77E-128
IL1A	-1.820093642	6.852722274	3.25E-137	2.50E-135
GADD45G	-1.821136217	-0.69445339	2.36E-10	8.49E-10
IMPA2	-1.822563317	2.810396159	7.23E-55	1.51E-53
FOSL1	-1.823960859	7.233008826	2.85E-120	1.73E-118
LAMA1	-1.824504475	1.327103922	1.06E-28	1.01E-27
GPR162	-1.828887849	-0.194336597	2.00E-13	9.01E-13
CYTIP	-1.832592381	2.479048995	1.07E-49	1.97E-48
CD6	-1.833287231	-1.453037868	1.78E-06	4.55E-06
PALM	-1.835869448	-0.818535107	2.43E-08	7.44E-08
NOTCH1	-1.84369077	5.995181604	4.09E-59	9.19E-58
ARAP3	-1.844486834	5.42427086	5.42E-88	2.08E-86
TMCC3	-1.848067025	2.706680973	6.55E-38	8.59E-37
BEX2	-1.852656325	2.371274169	1.32E-40	1.87E-39
KLK1	-1.854962819	-1.591078001	2.15E-05	4.86E-05
SCARF2	-1.856562898	1.837851643	1.67E-22	1.25E-21
KRTAP2-4	-1.85891696	-0.591152252	5.72E-09	1.84E-08
RTL3	-1.863798526	-1.29644771	1.67E-07	4.74E-07
HOXB7	-1.865965039	-0.582484367	5.89E-11	2.20E-10
TUB	-1.87611715	-0.041689307	5.08E-16	2.72E-15
ERVMER34-1	-1.884036905	0.286645797	3.34E-17	1.92E-16
PARD6A	-1.886947896	0.98961899	1.14E-19	7.43E-19
TOX2	-1.891326129	1.739235791	5.68E-33	6.48E-32
CGA	-1.893453916	-2.038948939	1.66E-04	3.36E-04
CSF3	-1.89473765	3.128936972	2.42E-60	5.69E-59
ADGRA2	-1.895056749	1.099398165	2.26E-28	2.14E-27
NEK3	-1.900245892	2.859739544	2.48E-57	5.38E-56
CLDND2	-1.90224869	-0.818177791	2.83E-10	1.01E-09
LCK	-1.907823792	-0.724830388	3.13E-10	1.11E-09
PPP1R1B	-1.912472757	-1.918859721	6.61E-05	1.41E-04
MCIDAS	-1.913669068	0.262606616	1.08E-15	5.69E-15
FOS	-1.919721946	3.325934823	2.35E-81	8.02E-80
MNX1	-1.921805626	0.604096916	1.19E-17	6.99E-17
GRAMD1B	-1.922906816	5.648973743	1.17E-127	7.96E-126
DLX2	-1.924763021	-1.629296515	2.62E-06	6.56E-06
TRABD2A	-1.928239996	-0.418920885	1.38E-13	6.28E-13
M1AP	-1.929035485	-0.964810472	1.36E-07	3.90E-07
TNFRSF1B	-1.931735151	3.944649618	1.88E-83	6.72E-82
CLCF1	-1.931954533	4.654781323	9.34E-100	4.30E-98
CEBPA	-1.938219649	-0.66430417	1.64E-07	4.67E-07
ROBO4	-1.93893435	6.136912302	5.40E-127	3.63E-125
CAMK2N1	-1.943916162	3.642423544	1.98E-60	4.67E-59
SGK1	-1.944990928	5.77582307	7.45E-115	4.20E-113

CD82	-1.948409171	5.974065729	4.65E-137	3.55E-135
FRZB	-1.966576877	-1.686124418	7.81E-06	1.86E-05
FAM20C	-1.968002951	3.829267753	2.47E-73	7.41E-72
PHGDH	-1.97075093	1.007168702	4.64E-31	4.89E-30
KRT86	-1.97627802	0.008854722	2.11E-17	1.23E-16
NT5DC4	-1.977155825	-1.592160424	3.10E-06	7.71E-06
ADAP2	-1.977743523	2.494833463	3.89E-60	9.08E-59
NYAP1	-1.981452201	-1.087414452	6.98E-09	2.23E-08
GAREM2	-1.987465752	0.976313206	4.10E-33	4.71E-32
SCN5A	-1.992996413	4.094931049	7.64E-88	2.92E-86
PLEKHO1	-2.000791927	4.194837035	1.94E-98	8.68E-97
ANKRD65	-2.007606079	-0.952480046	7.76E-10	2.68E-09
GFPT2	-2.011337092	4.650355356	2.51E-137	1.94E-135
UCP2	-2.017004144	4.683306095	1.62E-107	8.16E-106
EDNRB	-2.018931845	-0.205135414	1.50E-12	6.32E-12
CRLF2	-2.019107068	-0.347456383	4.20E-13	1.85E-12
CRADD	-2.021831885	4.201921878	1.73E-96	7.46E-95
DCLK1	-2.026594394	1.850427106	1.08E-32	1.22E-31
SEMA3F	-2.029218608	1.643061513	1.85E-48	3.24E-47
NFIB	-2.02997444	3.784393592	3.20E-81	1.08E-79
ADAP1	-2.03692053	2.165118435	2.36E-26	2.07E-25
EXPH5	-2.038816691	4.020059531	2.36E-53	4.72E-52
MFSD2A	-2.042122644	4.671957299	9.11E-135	6.82E-133
IL15RA	-2.046310776	4.164794129	2.59E-92	1.04E-90
KIF7	-2.047231914	3.146881461	1.83E-56	3.91E-55
NRROS	-2.051143519	2.758223025	3.39E-62	8.25E-61
NAT8L	-2.051326444	1.150116141	3.38E-29	3.30E-28
ASNS	-2.067347621	7.003645438	3.97E-171	4.27E-169
SERPINB2	-2.067716188	6.3379143	1.90E-184	2.22E-182
PLAU	-2.071708654	6.73926303	2.98E-194	3.63E-192
SOCS1	-2.074804253	-0.131317584	3.81E-17	2.18E-16
SLC1A3	-2.08019635	0.706194784	6.45E-31	6.76E-30
RTN4RL2	-2.084792042	0.698527483	9.52E-19	5.92E-18
GCNA	-2.086588891	1.777878011	2.51E-51	4.81E-50
TCEA3	-2.091510433	3.71689002	1.75E-92	7.07E-91
HHIP	-2.095454439	1.518401326	2.23E-35	2.73E-34
FOSB	-2.095587131	2.508282395	9.73E-64	2.44E-62
CXCL2	-2.096581254	5.664886944	7.57E-182	8.61E-180
RNF43	-2.106422205	2.762921375	2.09E-51	4.01E-50
PCSK9	-2.110799896	3.550366641	2.73E-61	6.58E-60
FUT1	-2.114953907	3.573531933	9.07E-109	4.64E-107
KIF21B	-2.115433213	2.446202042	5.32E-49	9.56E-48
ABCG4	-2.125330146	0.830412365	6.15E-31	6.46E-30
PSAT1	-2.125893613	6.386301039	6.46E-107	3.22E-105
GABRE	-2.126075653	1.569757126	6.39E-48	1.10E-46
AZGP1	-2.141203123	-1.76685819	3.24E-06	8.04E-06
CNKSR3	-2.141271679	3.109905409	3.61E-87	1.35E-85

KLF2	-2.141772088	0.944611278	2.41E-32	2.68E-31
MRC2	-2.150496427	4.728143489	7.38E-111	3.91E-109
CXCL8	-2.151850788	9.503678896	1.79E-109	9.27E-108
IL4I1	-2.153312398	-0.685166848	6.74E-12	2.72E-11
AIF1L	-2.155800976	2.663775379	3.15E-67	8.57E-66
RTN4RL1	-2.166602569	-0.317100898	1.47E-14	7.20E-14
GRAMD2A	-2.173645104	0.639857489	1.99E-32	2.22E-31
NIBAN1	-2.176095531	5.698323306	5.56E-143	4.50E-141
KRTAP2-1	-2.184748556	-1.851388469	2.46E-06	6.18E-06
CLDN2	-2.18989724	-1.280497124	3.03E-09	9.99E-09
PIWIL2	-2.194854684	-1.62852679	9.59E-07	2.53E-06
ARHGEF4	-2.197720946	3.153014298	6.87E-75	2.11E-73
P3H3	-2.198231656	3.032049568	1.63E-73	4.89E-72
TM4SF1	-2.201759004	7.052210234	3.56E-195	4.42E-193
OLFML2B	-2.202362228	-0.989128211	5.48E-11	2.05E-10
LAMP3	-2.203015811	4.911491461	1.46E-131	1.04E-129
NXPH4	-2.20463457	1.353080056	1.96E-40	2.75E-39
MPP4	-2.205079919	1.583567878	1.80E-40	2.53E-39
NFATC4	-2.206839938	-0.647187382	6.99E-14	3.27E-13
TNC	-2.210321531	6.757078145	1.09E-223	1.66E-221
DDIT4	-2.213157854	5.550322167	5.16E-152	4.64E-150
COL6A3	-2.213482699	2.710801786	9.12E-75	2.79E-73
PRKCE	-2.215057158	5.486273867	2.21E-83	7.82E-82
IL10RA	-2.219946439	-0.052342429	7.63E-18	4.53E-17
SPTBN2	-2.231483742	5.170134455	2.52E-104	1.22E-102
ATP6V0D2	-2.23801809	1.441962382	1.17E-46	1.94E-45
FAM149A	-2.244122739	-1.02535567	1.89E-11	7.33E-11
KCNQ4	-2.249758662	2.368617118	1.18E-51	2.27E-50
LCN2	-2.265189734	5.991585749	6.68E-130	4.67E-128
LAMA4	-2.268980684	-0.45222892	4.56E-11	1.71E-10
EN2	-2.269095861	1.125724256	3.43E-44	5.32E-43
KRTAP2-3	-2.281506306	2.683201958	1.94E-63	4.81E-62
NNMT	-2.320844135	6.520260022	3.80E-193	4.51E-191
DUSP9	-2.321067531	-0.054016855	1.04E-18	6.44E-18
DEPDC7	-2.323646747	2.130116266	1.01E-44	1.60E-43
PTPRU	-2.32840436	4.467549345	1.50E-114	8.37E-113
MKX	-2.346088355	0.535406634	8.62E-29	8.27E-28
SLC4A4	-2.346490826	-0.302298654	1.79E-17	1.04E-16
GNB3	-2.350735584	-1.419520309	1.05E-08	3.31E-08
RAB3IL1	-2.358451566	3.686353128	1.68E-94	6.99E-93
GRASP	-2.359903437	-1.852117754	7.06E-07	1.89E-06
SLC22A31	-2.361746876	1.97681563	1.29E-57	2.82E-56
FOXA2	-2.379135134	-0.272186904	1.97E-16	1.08E-15
NRARP	-2.38873608	0.519268511	5.63E-32	6.19E-31
PID1	-2.389116788	1.581965124	9.53E-60	2.19E-58
SAPCD2	-2.397142093	4.957059173	3.56E-119	2.12E-117
ACOXL	-2.398352855	1.647893698	1.69E-62	4.16E-61

TRIB3	-2.398817602	6.307632638	9.88E-153	8.95E-151
ADRA2C	-2.399404159	-0.90598273	1.91E-13	8.65E-13
CLDN3	-2.411836452	-0.026672182	1.07E-12	4.56E-12
ADORA2B	-2.415985378	4.665593723	1.62E-170	1.72E-168
DDN	-2.418903712	0.407640944	1.65E-31	1.78E-30
CLMN	-2.423326066	4.491969797	9.48E-102	4.44E-100
DMBT1	-2.436144467	2.466516905	8.73E-23	6.62E-22
TMEM52	-2.437357762	1.481963603	6.17E-49	1.10E-47
TEX48	-2.447094613	-1.453615486	2.79E-09	9.23E-09
PSCA	-2.454011626	-1.786665957	5.10E-07	1.39E-06
HOXB9	-2.463844983	4.928533334	1.54E-194	1.90E-192
MLXIPL	-2.483731275	-0.110624706	5.74E-21	4.00E-20
FA2H	-2.490634503	-1.419680991	6.00E-10	2.09E-09
CLDN4	-2.494622515	6.259952331	1.18E-189	1.39E-187
GRIN1	-2.497175995	-1.309363972	5.13E-07	1.39E-06
MPZL2	-2.51039013	4.220540052	2.31E-79	7.62E-78
RAP1GAP2	-2.536713105	5.291358678	9.16E-211	1.29E-208
BMPER	-2.538980092	3.017590005	2.36E-95	1.00E-93
HSPA1B	-2.544777693	10.16538228	8.03E-318	2.26E-315
LCN10	-2.55476472	-1.591677121	4.03E-09	1.31E-08
PRTN3	-2.572915815	-1.989255647	7.68E-07	2.05E-06
HPCAL4	-2.580814195	-0.838708714	2.17E-14	1.05E-13
SLC12A7	-2.589071486	5.495982437	4.80E-161	4.75E-159
HTR1D	-2.600422064	0.284751747	9.57E-32	1.04E-30
EREG	-2.627808705	6.124867905	1.40E-84	5.03E-83
CLPSL2	-2.630532782	-1.41937686	5.13E-11	1.92E-10
TLE2	-2.644825358	-1.024558938	1.79E-12	7.50E-12
HSPA1A	-2.646867604	9.954978872	0.00E+00	0.00E+00
MAP3K15	-2.648503291	-0.104620511	1.54E-23	1.21E-22
PDGFRB	-2.64936216	2.752320078	2.05E-107	1.03E-105
TBL1X	-2.672347612	5.630300928	5.67E-215	8.13E-213
RGS2	-2.686272666	0.974393468	2.66E-44	4.14E-43
LRP4	-2.687669491	2.871051263	7.58E-118	4.44E-116
EEF1A2	-2.700877206	4.004754187	1.08E-87	4.11E-86
CRABP2	-2.710810542	1.712831944	1.88E-55	3.95E-54
PRDM16	-2.719826625	-1.469920147	6.04E-11	2.25E-10
LRP3	-2.739745554	2.381921995	6.70E-53	1.32E-51
COLEC10	-2.752565985	-0.015263019	2.62E-26	2.29E-25
MYCL	-2.774342601	1.275530675	4.61E-50	8.58E-49
BMP5	-2.784995317	-1.420192524	1.06E-11	4.17E-11
GAL	-2.789068145	1.846409303	1.81E-67	4.93E-66
CNN1	-2.815140263	0.825702575	9.55E-28	8.81E-27
RBPMS2	-2.83141289	2.230685813	7.11E-98	3.14E-96
SAMD11	-2.838798274	0.989942492	5.47E-52	1.06E-50
KRT81	-2.860204561	3.844017044	9.71E-66	2.55E-64
MMP3	-2.860574811	5.996184336	3.35E-149	2.90E-147
JDP2	-2.862183564	2.484377847	2.12E-121	1.33E-119

TAGLN3	-2.902197965	-0.815680165	2.30E-14	1.11E-13
AREG	-2.914519738	5.005306438	2.36E-211	3.35E-209
CDKN1C	-2.921501656	1.177672712	6.56E-64	1.65E-62
CSPG4	-2.943453978	3.133454842	1.18E-93	4.88E-92
MMP1	-2.977564372	8.238964871	2.03E-299	4.74E-297
RASGRP2	-3.00750621	-1.520812144	2.67E-11	1.02E-10
FHL1	-3.008872056	2.056425322	6.30E-83	2.21E-81
PAPPA	-3.039056174	2.807888754	1.70E-75	5.30E-74
NRP2	-3.047686132	3.372495835	1.03E-162	1.04E-160
GDF15	-3.0550707	-0.456236265	9.38E-14	4.33E-13
APLN	-3.067443211	2.273276169	1.11E-99	5.08E-98
EPB41L4B	-3.123625495	2.980668549	6.66E-92	2.66E-90
FAM9B	-3.148601699	-0.378435634	9.89E-25	8.15E-24
SLC7A2	-3.161006422	4.150098766	2.89E-97	1.27E-95
GEM	-3.161349063	3.507398097	6.82E-81	2.28E-79
RRAD	-3.195554226	4.283895272	2.52E-134	1.86E-132
IL1B	-3.200416739	7.162653569	0.00E+00	0.00E+00
LTF	-3.221048334	-0.553591561	7.65E-14	3.57E-13
HES7	-3.235189946	0.753016782	1.60E-43	2.44E-42
ARC	-3.254157691	2.994171496	3.15E-88	1.22E-86
SBK2	-3.289162233	-2.088747554	2.04E-08	6.26E-08
CHST6	-3.350444816	1.205444485	6.83E-51	1.29E-49
HTRA1	-3.365376391	1.814987277	7.05E-89	2.73E-87
HSPA6	-3.390237269	5.313280257	1.15E-227	1.79E-225
IL24	-3.390915774	5.804775904	4.94E-323	1.55E-320
KLF15	-3.402741126	0.28649925	1.09E-33	1.27E-32
G0S2	-3.455673771	6.310025948	4.74E-261	9.07E-259
KLHDC7B	-3.459546446	-1.964296691	4.13E-09	1.34E-08
BATF3	-3.489185166	2.183597989	9.87E-125	6.44E-123
AKAP12	-3.510621633	4.92307936	8.70E-278	1.96E-275
KRTAP4-8	-3.520463073	-0.962637106	6.87E-19	4.31E-18
IL11	-3.598485632	6.668399461	0.00E+00	0.00E+00
CST7	-3.627385646	2.110350221	3.72E-109	1.92E-107
SPNS3	-3.645414991	-1.356010643	6.04E-15	3.03E-14
PLPP4	-3.7499368	0.480841248	1.18E-53	2.37E-52
LAIR1	-3.771298328	-1.468999016	1.56E-14	7.62E-14
SERTAD4	-3.823017427	1.918108242	1.25E-102	5.97E-101
LARGE2	-3.954357661	0.330924839	2.79E-53	5.55E-52
NUPR1	-3.967564017	-0.733686488	3.98E-27	3.59E-26
KRTAP4-11	-4.877743254	-2.224123898	1.81E-10	6.57E-10

Supplemental Table 5. Genes significantly regulated following 5 days of 1nM E2 treatment of ER$\beta^{DBD\text{-}Mut}$ expressing MDA-MB-231 cells.

Gene	logFC	logCPM	PValue	FDR

KRT6A	7.926587756	-1.417840941	1.56E-08	1.48E-05
DEFB4A	7.48635398	0.611647648	6.89E-10	9.15E-07
DEFB4B	7.48635398	0.611647648	6.89E-10	9.15E-07
HBA1	6.922976482	-0.723436884	1.73E-07	1.09E-04
IVL	6.83691399	-0.722052636	3.53E-17	1.17E-13
HBA2	6.479375819	-1.140621052	6.11E-07	3.12E-04
ALOX15B	6.216805393	2.560359688	6.04E-26	4.01E-22
SLPI	5.898166123	2.622883421	6.78E-10	9.15E-07
PI3	5.390945883	-1.465671114	3.02E-05	9.32E-03
MYOZ2	4.966311818	1.228951165	2.35E-26	3.13E-22
CRYBA2	4.432403807	0.2206735	2.35E-05	7.43E-03
WNT7B	3.726738359	1.958330263	3.53E-07	1.98E-04
LRRC26	3.616124626	0.12381992	8.70E-05	2.27E-02
SLAMF8	3.59482603	2.308887941	1.04E-19	4.60E-16
EDN2	3.441633088	3.638064023	1.15E-14	3.06E-11
IGFBP6	3.430127494	6.849404768	2.03E-04	4.73E-02
DEPP1	3.343756258	5.036951868	4.37E-12	9.67E-09
CXCL14	3.327378313	0.472106604	7.29E-09	8.07E-06
KRT7	3.202493177	8.430485573	6.68E-05	1.93E-02
CSF3	3.190829627	4.061688589	3.85E-06	1.64E-03
CHI3L2	3.008796754	-1.153573578	7.64E-05	2.11E-02
TENM4	2.993518865	-1.7314875	1.08E-05	3.87E-03
ANGPTL4	2.986918433	3.038952509	1.32E-04	3.37E-02
COL6A2	2.887875803	6.850178692	3.08E-04	6.83E-02
PDE6A	2.833925546	-0.048414718	3.08E-11	5.85E-08
RTN4RL1	2.799317667	0.916918977	8.93E-07	4.40E-04
DAAM2	2.788739416	-1.749996441	3.04E-06	1.35E-03
DISP3	2.661166478	-1.278407728	1.68E-06	7.70E-04
RHOB	2.609745945	5.75519619	5.13E-06	2.07E-03
LCN2	2.482679198	3.982492541	6.99E-05	1.98E-02
VSTM2L	2.427735157	6.023599193	4.57E-04	9.48E-02
PGF	2.42543312	2.525460847	2.12E-04	4.87E-02
GPC1	2.385273166	6.382954258	1.36E-04	3.42E-02
BEAN1	2.360839491	-1.054716232	1.21E-06	5.75E-04
C1QTNF1	2.347895861	3.071927842	1.93E-04	4.65E-02
AATK	2.341996876	-0.230405598	3.94E-06	1.64E-03
TRIM29	2.332401578	2.838495008	3.78E-05	1.14E-02
VPS37D	2.297536253	1.166378358	4.66E-04	9.52E-02
COL6A1	2.17062837	7.210819182	1.54E-07	1.09E-04
RETREG1	2.144331895	2.102179215	5.76E-06	2.25E-03
ALPP	2.063094367	2.955913938	9.25E-06	3.42E-03
TGM5	2.030431188	-0.392225031	1.98E-05	6.56E-03
LOXL4	2.020742995	6.593179013	1.24E-08	1.27E-05
NGFR	2.000937797	2.092834671	5.60E-07	2.98E-04
PPP1R3G	2.000391905	0.751465158	8.58E-05	2.27E-02
GRIN2C	1.941571166	-1.107749687	1.83E-04	4.50E-02
ALPG	1.886957575	-0.632804213	1.61E-05	5.63E-03

CYTH4	1.813225982	-1.215943685	4.26E-04	8.98E-02
PPL	1.768850016	1.380763404	2.89E-07	1.74E-04
HMOX1	1.74118709	5.913957941	2.29E-05	7.42E-03
ITGB4	1.695319936	6.722234313	4.08E-04	8.75E-02
C3	1.652223885	5.583087249	7.57E-06	2.88E-03
TSC22D3	1.384974032	4.149161569	8.13E-05	2.20E-02
TFCP2L1	1.246451189	1.990767003	2.76E-04	6.21E-02
SERPINB2	-1.2963964	0.54264222	3.40E-04	7.41E-02
S1PR3	-1.741698145	-0.692740813	1.96E-04	4.65E-02
S1PR3	-1.741698145	-0.692740813	1.96E-04	4.65E-02
ESR2	-1.955459498	8.032360741	3.57E-07	1.98E-04
GCNA	-2.109128048	0.783615059	3.39E-08	3.00E-05
RFPL4AL1	-2.609023331	-0.706895533	1.56E-07	1.09E-04
RFPL4A	-2.782664203	-0.049540165	1.65E-07	1.09E-04
DTNA	-2.876858586	-2.107068282	4.57E-05	1.35E-02
LUM	-3.101158869	-0.541487119	3.73E-09	4.51E-06

Supplemental Table 6. Genes commonly regulated following 5 days of 1nM E2 treatment of both WT ERβ and ERβ$^{DBD\text{-}Mut}$ expressing MDA-MB-231 cells.

Genes up-regulated by E2 in both cell lines

Gene	logFC WT Erβ	logFC ERβ^{Mut}
CXCL14	8.448357823	3.327378313
LOXL4	7.415968031	2.020742995
CYTH4	6.948277338	1.813225982
ALPG	5.307381322	1.886957575
ALPP	5.297696386	2.063094367
RETREG1	4.62090557	2.144331895
IVL	3.469141956	6.83691399
C3	3.117849951	1.652223885
GRIN2C	2.719966346	1.941571166
CHI3L2	2.453118241	3.008796754
IGFBP6	2.316661564	3.430127494
WNT7B	2.280388357	3.726738359
GPC1	2.181527809	2.385273166
HMOX1	1.867729489	1.74118709
DEPP1	1.615979834	3.343756258
ANGPTL4	1.429205438	2.986918433
MYOZ2	1.206110092	4.966311818
PPP1R3G	1.047402844	2.000391905
TENM4	1.012639101	2.993518865

Genes down-regulated by E2 in both cell lines

Gene	logFC WT Erβ	logFC ERβ^{Mut}
SERPINB2	-2.067716188	-1.2963964

GCNA	-2.086588891	-2.109128048
